# Assessment of Racial Disparities in Primary Care Physician Specialty Referrals

**DOI:** 10.1001/jamanetworkopen.2020.29238

**Published:** 2021-01-25

**Authors:** Bruce E. Landon, Jukka-Pekka Onnela, Laurie Meneades, A. James O’Malley, Nancy L. Keating

**Affiliations:** 1 Department of Health Care Policy, Harvard Medical School, Boston, Massachusetts; 2 Division of General Internal Medicine, Department of Medicine, Beth Israel Deaconess Medical Center, Boston, Massachusetts; 3 Department of Biostatistics, Harvard School of Public Health, Boston, Massachusetts; 4 The Dartmouth Institute for Health Policy and Clinical Practice, Geisel School of Medicine at Dartmouth, Hanover, New Hampshire; 5 Department of Biomedical Data Science, Geisel School of Medicine at Dartmouth, Hanover, New Hampshire; 6 Division of General Internal Medicine, Department of Medicine, Brigham and Women’s Hospital, Boston, Massachusetts

## Abstract

**Importance:**

Disparities in quality of care according to patient race and socioeconomic status persist in the US. Differential referral patterns to specialist physicians might be associated with observed disparities.

**Objective:**

To examine whether differences exist between Black and White Medicare beneficiaries in the observed patterns of patient sharing between primary care physicians (PCPs) and physicians in the 6 specialties to which patients were most frequently referred.

**Design, Setting, and Participants:**

This cross-sectional observational study of Black and White Medicare beneficiaries used claims data from 2009 to 2010 on 100% of traditional Medicare beneficiaries who were seen by PCPs and selected high-volume specialists in 12 health care markets with at least 10% of the population being Black. Statistical analyses were conducted from December 20, 2017, to September 30, 2020.

**Exposures:**

Differences in patterns of patient sharing among Black and White patients.

**Main Outcomes and Measures:**

Primary care physician and specialist degree (the number of other PCPs or specialists to whom each physician is connected) and strength (the number of shared patients per connection, overall, for Black patients and White patients and after equalizing the numbers of Black and White patients per PCP), as well as distance between PCP and patient and specialist zip code centroids.

**Results:**

The 12 selected markets ranged in size from Manhattan, New York (187 054 Black or White beneficiaries seen by at least 2 physicians within an episode of care; 9794 total physicians), to Tallahassee, Florida (44 644 Black or White beneficiaries seen by at least 2 physicians within an episode of care; 847 total physicians). The percentage of Black beneficiaries ranged from 11.5% (Huntsville, Alabama) to 46.8% (Chicago, Illinois). The mean PCP-specialist degree (number of specialists with whom a PCP shares patients) was lower for Black patients than for White patients. For instance, the mean PCP-cardiologist degree across all markets for White patients was 17.5 compared with 8.8 for Black patients. After sampling White patients to equalize the numbers of patients seen, the degree differences narrowed but were still not equivalent in many markets (eg, for all specialties in Baton Rouge, Louisiana: 4.5 for Black patients vs 5.7 for White patients). Specialist networks among White patients were much larger than those constructed based just on Black patients (eg, for cardiology across all markets: 135 for Black patients vs 330 for White patients), even after equalizing the numbers of patients seen per PCP (123 for Black patients vs 211 for White patients). The overall test for differences in referral patterns was statistically significant for all 6 specialties examined in 7 of the 12 markets and in 5 specialties for another 3.

**Conclusions and Relevance:**

This study suggests that differences exist in specialist referral patterns by race among Medicare beneficiaries. This is an observational study, and thus some differences might have resulted from patient-initiated visits to specialists.

## Key Points

**Question:**

Does patient sharing among primary care physicians and specialists differ based on the patient’s race?

**Findings:**

In this cross-sectional study of Medicare beneficiaries, primary care physicians shared Black patients with fewer specialists relative to White patients, even after sampling White patients to equalize the number of patients seen.

**Meaning:**

This study suggests that differences exist in specialist referral patterns by race among Medicare beneficiaries.

## Teaser

This cross-sectional study examines whether differences exist between Black and White Medicare beneficiaries in the observed patterns of patient sharing between primary care physicians and physicians in the 6 specialties to which patients were most frequently referred.

## Introduction

For several decades, significant problems with the quality of care and disparities in quality according to patient race and socioeconomic status have been documented in the US health care system.^1,2,3,4,5,6,7,8,9,10,11,12,13^ Despite quality improvement efforts, whether general or aimed at specific underserved populations, little progress has been made in attenuating disparities. Most recently, the 2018 National Healthcare Quality and Disparities Report concluded that “Overall, some disparities were getting smaller from 2000 through 2016-2017, but disparities persist, especially for poor and uninsured populations in all priority areas.”^13^^(p1)^

Although there are many factors associated with disparities in care and outcomes, including adequacy of insurance coverage,^14^ neighborhood issues related to food or housing scarcity or insecurity,^15,16^ and lack of resources,^17^ at least some causes of disparities are mediated through health care professionals.^18,19^ Fundamentally, this can happen through 2 mechanisms. First, as initially shown by Bach and colleagues^20^ in a seminal 2004 article on primary care physicians (PCPs), and shown in several analyses of hospitals,^21,22,23,24,25^ underserved populations might obtain their care from physicians or hospitals that provide lower-quality care, as judged by accepted measures of quality. For instance, primary care visits by Black patients were concentrated with a smaller group of physicians who saw relatively few White patients.^20^ These physicians were less likely to be board certified and reported more difficulty obtaining access to high-quality specialists. Moreover, neighborhoods that are poor or underserved might have more difficulty attracting the highest-quality physicians, and hospitals in these neighborhoods might have relatively fewer resources because of the relatively low payment rates for Medicaid and other safety-net programs.

The second mechanism is associated with differences in the way that patients are treated by physicians when they see the same physicians who treat patients of different races and socioeconomic status. Although overt discrimination might be rare, implicit bias may lead physicians to provide different treatment to patients from underserved groups.^26^ This may manifest in differential recognition of diseases or symptoms owing to imperfect information transmittal (eg, statistical discrimination^27^). It also might manifest in referral decisions; referring physicians might assume that patients from a particular underserved group prefer not to travel far to see a specialist or prefer to see specialists of a specific type. Alternatively, physicians may perceive that specialists to whom they refer might be more or less willing to see underserved patients. Primary care physicians therefore might make different referral recommendations for different groups of patients. Whether such differential referral behavior exists, however, has not been formally studied, to our knowledge.

In this study, we use comprehensive administrative data from the Medicare program from markets with relatively large Black populations to explore whether differential patterns of specialty care can be observed empirically. Using methods from network science, we constructed patient-sharing networks of physicians by race for White and Black patients between PCPs and selected high-volume specialties in 12 health care markets with at least 10% of the population being Black. Patient-sharing networks are defined based on shared care for a common patient between any 2 physicians. We then tested whether differences in the observed patterns of patient sharing were statistically different.

## Methods

### Data Source and Study Markets

We used data on 100% of traditional Medicare beneficiaries from 51 hospital referral regions (HRRs) for the years 2009 to 2010 to examine patient-sharing networks among physicians. Sharing of patients based on administrative data can identify information-sharing ties among pairs of physicians, which we then used to infer information-sharing relationships among physicians. We identified the HRRs for which the percentage of Black patients as identified in the Beneficiary Summary File was 10% or more. From these 17 markets, we then restricted our analyses to the 12 markets with the greatest total number of physicians, all of which had at least 750 physicians (Table 1; eFigure 1 in the Supplement). The study was approved by the institutional review board at Harvard Medical School and was funded by the National Cancer Institute. Because the study involved millions of patients, the need for informed consent was waived, as is usual in these large retrospective database studies. Our study follows the Strengthening the Reporting of Observational Studies in Epidemiology (STROBE) reporting guideline for cross-sectional studies.^28^

**Table 1.** Description of Included Markets

Market	Patients, No.	Black patients, No. (%)	Edges, No.^a^	Physicians, No.	PCPs, No. (%)	Cardiology, No. (%)	Pulmonary, No. (%)	Gastroenterology, No. (%)	Orthopedics, No. (%)	General Surgery, No. (%)	Neurology, No. (%)
Chicago, Illinois	91 612	42 899 (46.8)	388 893	4222	1683 (39.9)	218 (5.2)	102 (2.4)	119 (2.8)	167 (4.0)	140 (3.3)	148 (3.5)
Memphis, Tennessee	105 954	33 013 (31.2)	233 593	1997	690 (34.6)	94 (4.7)	42 (2.1)	55 (2.8)	102 (5.1)	99 (5.0)	46 (2.3)
Baton Rouge, Louisiana	42 867	13 276 (31)	100 291	1062	356 (33.5)	49 (4.6)	21 (2.0)	38 (3.6)	64 (6.0)	51 (4.8)	26 (2.4)
Norfolk, Virginia	81 586	21 596 (26.5)	241 700	1721	648 (37.7)	76 (4.4)	39 (2.3)	47 (2.7)	70 (4.1)	69 (4.0)	49 (2.8)
Lafayette, Louisiana	45 012	10 876 (24.2)	66 815	768	281 (36.6)	31 (4.0)	12 (1.6)	15 (2.0)	39 (5.1)	49 (6.4)	13 (1.7)
Tallahassee, Florida	44 644	10 654 (23.9)	51 568	847	319 (37.7)	31 (3.7)	15 (1.8)	24 (2.8)	48 (5.7)	36 (4.3)	20 (2.4)
Manhattan, New York	187 054	33 909 (18.1)	854 752	9794	3170 (32.4)	645 (6.6)	220 (2.2)	372 (3.8)	356 (3.6)	399 (4.1)	369 (3.8)
Jacksonville, Florida	114 258	18 087 (15.8)	334 233	2383	848 (35.6)	125 (5.2)	60 (2.5)	90 (3.8)	89 (3.7)	97 (4.1)	75 (3.1)
Miami, Florida	95 071	14 495 (15.2)	478 154	4472	1659 (37.1)	273 (6.1)	99 (2.2)	124 (2.8)	170 (3.8)	172 (3.8)	162 (3.6)
San Bernardino, California	53 364	6452 (12.1)	135 532	2022	872 (43.1)	92 (4.5)	30 (1.5)	42 (2.1)	88 (4.4)	82 (4.1)	41 (2.0)
Buffalo, New York	58 400	7050 (12.1)	167 261	1982	767 (38.7)	89 (4.5)	30 (1.5)	40 (2.0)	84 (4.2)	89 (4.5)	67 (3.4)
Huntsville, Alabama	47 328	5441 (11.5)	82 174	827	339 (41.0)	39 (4.7)	12 (1.5)	23 (2.8)	48 (5.8)	30 (3.6)	18 (2.2)

Abbreviation: PCP, primary care physician.

^a^ Edges represent connections between any 2 physicians based on shared patients.

### Identifying the Sharing of Patients and Constructing Physician Networks

We defined encounters with physicians based on paid claims in the carrier file. We excluded claims for nondirect patient care specialties or specialties in which individual physicians are not typically selected by patients (eg, anesthesia or radiology). We identified all evaluation and management services and included procedures with a relative value unit of at least 2.0 to capture surgical procedures that often are reimbursed via bundled fees that include preprocedure and postprocedure assessments. We excluded claims for laboratory and other services not requiring a physician visit; we also excluded claims generated from physicians who saw fewer than 30 Medicare beneficiaries during any year or who practiced outside of the included HRRs. We obtained information on specialty and practice location directly from the claims.

We identified shared patient care between physicians in the context of care within a defined clinical episode.^29^ Episode-based network construction offers an improvement compared with existing methods because it results in networks composed of physician-physician ties with a greater likelihood of corresponding to real connections among the physicians; the resulting networks are also less dense than networks constructed from all instances of patient sharing.^30^ We identified discrete episodes of care using Symmetry Episode Treatment Groups, version 8.3 (Optum). Each episode of care groups clinically related services delivered to a patient with a specific condition over a defined period of time into 1 of approximately 600 different episode types, which reflect treatment for both chronic diseases (eg, diabetes) and acute conditions (eg, pneumonia or ankle fracture). A total of 92.2% of patient visits were assigned to episodes, and 46.5% of episodes had more than 1 visit associated with them. Each episode begins after a “clean” period of varying length during which no related encounters occurred.

We then constructed networks using 3 different approaches: 1 based only on encounters for White patients, 1 based only on encounters for Black patients, and 1 based on encounters for Black and White patients. We studied visits by Black and White beneficiaries only because sample sizes for other racial groups were not large enough to support our planned analyses.

All networks were constructed using encounters during the 2-year study period. To construct episode-based bipartite networks for each HRR separately, we considered a sequence of physician-episode pairs, in which each episode consists of 1 patient and 1 or more physicians who provided care to the patient during the given medical episode.^31^ After constructing bipartite graphs for each of these groups in each of the 12 markets, we constructed corresponding unipartite networks of physicians by projecting the underlying bipartite networks consisting of physician-episode ties. Each unipartite network was obtained from the corresponding bipartite network using the common projection technique; the bipartite adjacency matrix is first multiplied by its transpose, and the diagonal elements of the resulting matrix are then set to 0.

### Specialty-Specific Networks and Measures

We focused our analyses on shared care between physicians identified as PCPs (defined as general internal medicine, family medicine, general practice, or geriatrics) and physicians from the 6 specialties that we identified as the most frequently visited specialties (cardiology, pulmonary disease, gastroenterology, orthopedic surgery, general surgery, and neurology). We did not study nephrology visits because of the superimposed care of patients with end-stage kidney disease for whom nephrologists are often dictated by the dialysis facility. For each included market, we created the 3 types of networks already described (using care of White patients only, Black patients only, or both) that were limited to PCPs and each of the 6 specialties.

To visualize the resulting networks, we limited our analyses to PCPs who had seen at least 5 White patients and at least 5 Black patients during the 2-year period. In each market and for each specialty, we randomly sampled 3 sets of 15 PCPs and then graphed their connections with the specialty of interest for the Black network, the White network, and the combined network. For ease of interpretation, we fixed the physicians in Cartesian coordinates according to the zip code of their primary practice. Because physicians tended to congregate in specific zip codes, we added a small displacement to the coordinates for each PCP so that the nodes would not be overlapping.

### Statistical Analysis

Statistical analyses were conducted from December 20, 2017, to September 30, 2020. We first described the Black, White, and combined PCP-specialist networks in each of the 12 HRRs. For each PCP-specialty combination, we included the numbers of PCPs and specialists as well as the number of connections (edges) observed in each HRR for each type of PCP-specialist network. We calculated the mean number of connections of PCPs with physicians in each specialty (degree). We also calculated the mean spatial distance between the zip code centroids of the PCP and specialist as well as between the specialist and the beneficiary residence.

To quantify whether there were differences in the patterns of patient sharing between PCPs and each of the specialties in each of the markets, we first examined the proportion of an individual PCP’s patients that were Black or White and then compared this proportion with the proportion for each specialist within a specialty with whom that particular PCP shared patients. For example, if a PCP treated 50 Black patients and 50 White patients but 7 of the 10 patients shared with specialist A were Black, then the overall proportion of Black patients is 0.5, but the specialist A proportion of Black patients is 0.7. Using the overall proportion, we computed an expected number of Black patients referred by the PCP to each specialist; for specialist A in the example, the expectation is 5. We then calculated the square of the actual and expected counts, divided by the expected count, and then summed the resulting quantities across the set of qualifying specialists for each clinician. Let *y_ij_* and 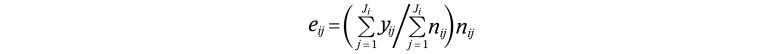
denote the actual and expected counts (under the null hypothesis of nonselective referral) of Black patients referred by PCP *i* to specialist *j* ϵ {1, …, *J_i_*} for the *J_i_* specialists to whom PCP *i* refers patients, where *n_ij_* is the total number of patients that PCP *i* shares with specialist *j*. The referral bias statistics for PCP *i* is then given by
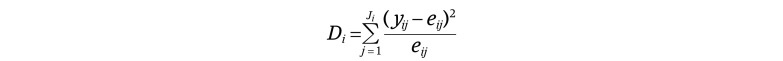
and, under the null hypothesis of nonselective referrals, has a χ^2^ distribution with *J_i_* − 1 *df*. A *P* value based on the Fisher exact test was evaluated, and PCPs were ranked by the χ^2^ statistic. Primary care physicians with the largest values of the test statistic are those for whom the evidence is greatest that their referrals are selective with respect to patient race. This approach allowed the pattern of referrals to be analyzed in depth for a sample of clinicians with varying degrees of observed referral bias. Finally, because power is limited for any individual physician, we summed the referral bias statistics across physicians to obtain an overall summary statistic for each market and specialty. The statistical significance of the resulting summary statistic was evaluated by applying a Cochran-Mantel-Haenszel test for stratified contingency tables (the PCPs are the strata).

All nonnetwork statistical analyses were performed with SAS, version 9.2 (SAS Institute Inc).^32^**All network-related analyses were implemented with Python, version 3.7.3 (Python Software Foundation) or R, version 3.5.1 (R Foundation for Statistical Computing).

## Results

The 12 selected markets ranged in size from Manhattan, New York (187 054 Black or White beneficiaries seen by at least 2 physicians within an episode of care; 9794 total physicians), to Tallahassee, Florida (44 644 beneficiaries seen by at least 2 physicians within an episode of care; 847 total physicians) (Table 1). The number of PCPs ranged from 281 in Lafayette, Louisiana, to 3170 in Manhattan, New York. The 12 markets were distributed throughout the US, although concentrated mostly in the southeast (eFigure 1 in the Supplement). The percentage of Black Medicare beneficiaries was highest in Chicago, Illinois (46.8% Black) and lowest in Huntsville, Alabama (11.5% Black) (Table 1).

**Figure 1.**
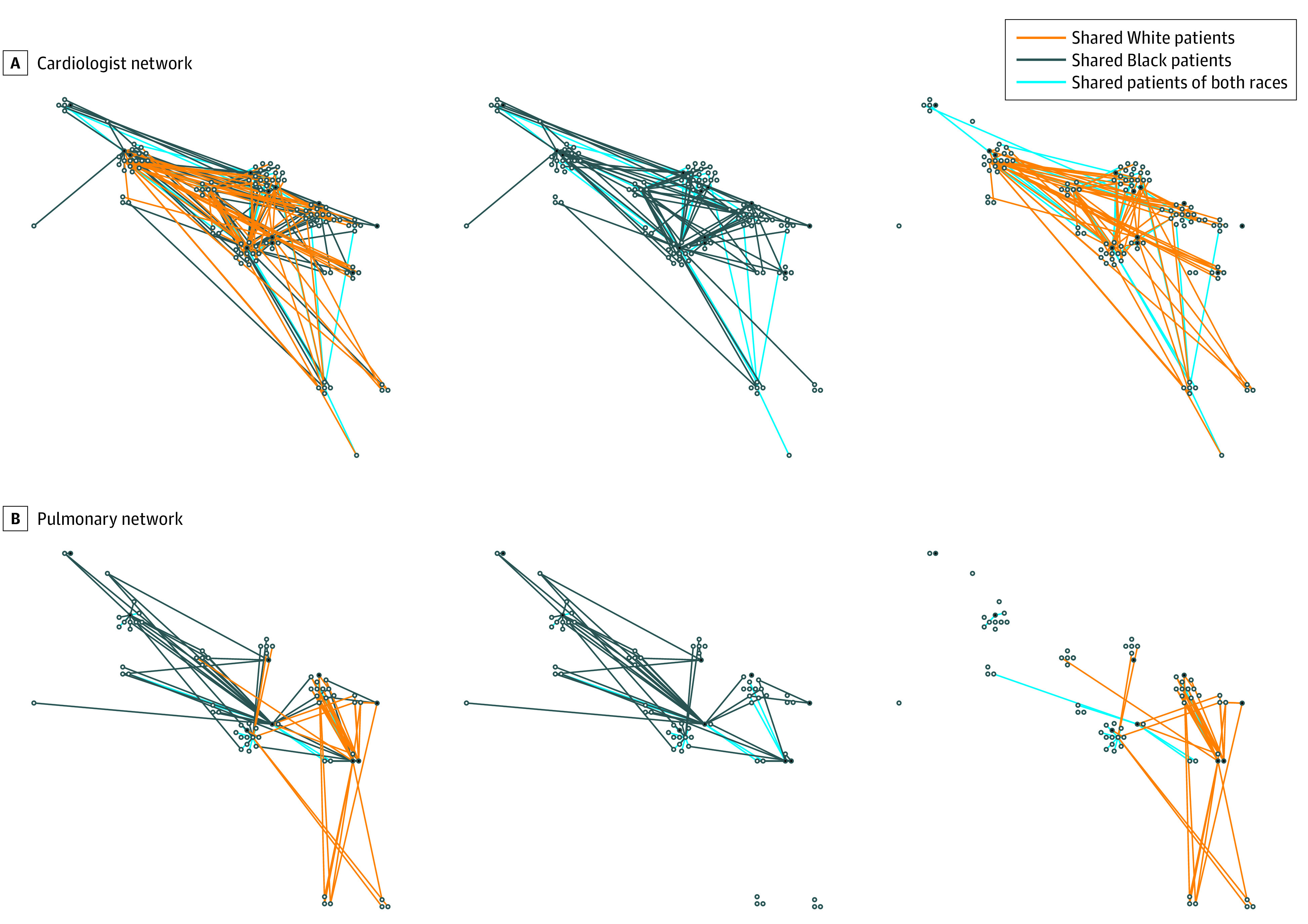
Graphical Representations of the Networks Formed by 15 Sampled Primary Care Physicians and Associated Specialists A, Chicago, Illinois, cardiologist network. B, Chicago, Illinois, pulmonologist network. Black lines indicate shared Black patients only. Orange lines indicate shared White patients only. Blue lines indicate shared patients of both races.

On average, PCPs shared patients with 6.3 to 14.3 specialist physicians in each of the 6 specialties (PCP degree; Table 2). Graphical representations of the networks formed by 15 sampled PCPs and associated specialists in cardiology and pulmonology are shown in Figure 1, in which thicker edges depict a higher number of shared patients (shared White patients are depicted by orange lines, Black patients by black lines, and both by blue lines [only in the combined graphs]). The full collection of 3 random samples of 15 PCPs for each specialty in each market is included in eFigure 2 in the Supplement. Physicians in Jacksonville, Florida, and Norfolk, Virginia, generally shared patients with a larger number of specialists per specialty (14.3 and 13.7, respectively), while those in San Bernardino, California, and Tallahassee, Florida, shared patients with a smaller number of specialists per specialty (5.6 and 6.3, respectively). Across all markets, PCPs shared patients with more cardiologists (20.0) and fewer general surgeons (6.8). Across all specialties, with the exception of Chicago, Illionois, the mean PCP-specialist degree (ie, the mean degree of PCPs when considering referrals only to that specialty) was lower for Black patients than for White patients. For instance, the mean PCP-cardiologist degree across all markets for White patients was 17.5 compared with 8.8 for Black patients, equating to 87% of the overall degree for White patients and 45% of the overall degree for Black patients (Table 2). This finding was mirrored when assessing specialist degree—the number of other physicians with whom specialists share care (specialists share care with far more PCPs than vice versa; Table 2). For instance, the mean degree of specialist in Memphis, Tennessee, was 245.3 for White patients and 159.2 for Black patients, representing 77% of the overall degree for White patients and 48% of the overall degree for Black patients.

**Table 2.** Network Measures by Market and Specialty Combined and After Stratifying by Patient Race

Market	Specialty	PCP degree: all^a^	Black, No. (%)^b^	White, No. (%)^b^	Specialist degree^a^	Black, No. (%)^a^	White, No. (%)^a^	PCP strength^c^	Black, No. (%)^a^	White, No. (%)^a^
Chicago, Illinois	All	7.3	5.4 (75)	5.1 (72)	180.0	108.6 (59)	103.0 (58)	2.0	1.7 (86)	1.8 (93)
Memphis, Tennessee	All	13.5	7.5 (56)	11.2 (83)	317.1	159.2 (48)	245.3 (77)	3.5	2.4 (70)	3.1 (88)
Baton Rouge, Louisiana	All	10.7	6.1 (58)	9.0 (84)	229.3	117.6 (50)	185.3 (80)	3.2	2.1 (67)	2.7 (86)
Norfolk, Virginia	All	13.7	6.8 (50)	11.8 (86)	355.3	166.2 (45)	295.7 (83)	3.2	2.0 (64)	2.8 (88)
Lafayette, Louisiana	All	8.3	4.1 (51)	7.3 (89)	216.4	100.6 (46)	193.4 (89)	4.4	2.5 (58)	3.7 (86)
Tallahassee, Florida	All	6.3	3.6 (59)	5.8 (91)	156.2	77.9 (49)	137.8 (88)	5.3	2.6 (51)	4.6 (86)
Manhattan, New York	All	9.4	3.4 (38)	8.8 (94)	189.7	44.9 (23)	164.7 (87)	2.0	1.5 (77)	2.0 (97)
Jacksonville, Florida	All	14.3	5.9 (41)	12.8 (89)	319.7	110.3 (34)	281.9 (88)	3.4	1.9 (57)	3.2 (94)
Miami, Florida	All	10.5	4.4 (43)	9.4 (90)	258.0	72.5 (27)	223.3 (87)	2.1	1.5 (71)	2.0 (95)
San Bernardino, California	All	5.6	2.5 (47)	5.2 (92)	157.4	43.8 (27)	142.5 (91)	2.7	1.5 (56)	2.6 (95)
Buffalo, New York	All	8.7	3.1 (38)	8.0 (93)	207.5	45.7 (21)	188.2 (91)	2.4	1.5 (62)	2.3 (97)
Huntsville, Alabama	All	10.4	3.5 (36)	9.8 (95)	268.8	80.3 (28)	252.9 (94)	3.1	1.5 (50)	2.9 (95)
All	Cardiology	20.0	8.8 (45)	17.5 (87)	329.9	134.8 (40)	281.3 (85)	4.1	2.3 (58)	3.7 (91)
All	Pulmonary	8.1	4.4 (56)	7.2 (89)	325.4	142.3 (44)	277.3 (85)	4.1	2.3 (57)	3.7 (91)
All	Gastroenterology	7.9	4.0 (51)	7.0 (89)	239.5	95.8 (39)	201.9 (84)	3.1	1.9 (65)	2.8 (90)
All	Orthopedics	8.7	3.3 (39)	7.8 (91)	137.5	38.5 (28)	119.5 (87)	2.1	1.4 (68)	2.0 (96)
All	General surgery	6.8	3.7 (55)	5.7 (85)	141.7	57.0 (41)	114.3 (80)	2.6	1.7 (69)	2.3 (91)
All	Neurology	7.9	3.9 (51)	6.9 (87)	253.7	95.4 (37)	212.6 (84)	2.7	1.8 (67)	2.5 (91)

Abbreviation: PCP, primary care physician.

^a^ Degree is the number of ties between PCPs and specialists. All represents the mean across physicians in each of the 6 specialties across all included markets.

^b^ Black and White refer to the percentage of the overall measure present in the networks constructed using Black or White patients only.

^c^ Strength is the number of shared patients.

One explanation for the observed differences is that, on average, PCPs have more White patients than Black patients. Thus, we also recalculated degree and strength after randomly sampling among the White patients so that there were equal numbers of White and Black patients per PCP (eTable 2 in the Supplement). In this case, the PCP degree differences narrowed but were still not equivalent (eg, for all specialties in Baton Rouge, Louisiana: 4.5 for Black patients vs 5.7 for White patients) (eTable 1 in the Supplement). In addition, after randomly sampling among the White patients so that there were equal numbers of White and Black patients per PCP, there was more heterogeneity across markets; for instance, PCP degree for Black patients in San Bernardino, California (2.5), was no longer lower than for White patients (2.6). When examining specialist degree, however, the results were similar to the overall results, with specialist networks among White patients being much larger than those constructed based just on Black patients (eg, for cardiology across all markets: 135 for Black patients vs 330 for White patients), even after equalizing the numbers of patients seen per PCP (123 for Black patients vs 211 for White patients).

Another explanation for the differences in referral patterns may be associated with geographical location within a market. We examined the difference traveled in 2 different ways. We examined the distance between the patient’s zip code of residence and the specialist’s zip code as well as the distance between the PCP’s office and the specialist’s office zip codes. Across the 6 specialties, Black patients generally traveled a shorter distance than White patients to their physicians (generally 80%-90% of the overall distance traveled; for instance, for cardiology across all markets, Black patients generally traveled 22.1 vs 251 km [13.8 vs 15.7 miles] for White patients, equal to 89% of the mean distance traveled for Black patients and 102% of the mean distance traveled for White patients), although this finding was reversed for general surgeons, for whom Black patients traveled a slightly greater distance than White patients (22.2 vs 21.4 km [13.9 vs 13.4 miles] across all markets; Table 3). There was heterogeneity across the markets, however; Black and White patients traveled the same distance in San Bernadino, California, and Miami, Florida, but Black patients traveled just over half the distance as White patients in Buffalo, New York. Distance between the PCP’s office and the specialist’s office followed a similar pattern, and these findings were consistent across all of the specialties.

**Table 3.** Distance Measures by Market and Specialty Combined and After Stratifying by Patient Race

Market	Type of specialist	PCP to specialist distance, km (miles)^a^	Black, No. (%)^b^	White, No. (%)^b^	Patient to specialist distance, km (miles)^a^	Black, No. (%)^b^	White, No. (%)^b^
Chicago, Illinois	All	4.6 (2.9)	3.1 (106)	2.7 (93)	15.2 (9.5)	9.2 (97)	9.7 (102)
Memphis, Tennessee	All	14.4 (9.0)	8.8 (98)	9.1 (101)	26.9 (16.8)	16.1 (96)	17.2 (102)
Baton Rouge, Louisiana	All	11.7 (7.3)	7.1 (98)	7.3 (101)	21.8 (13.6)	12.8 (94)	13.9 (103)
Norfolk, Virginia	All	15.5 (9.7)	8.8 (91)	10.0 (103)	23.5 (14.7)	14.0 (95)	14.9 (102)
Lafayette, Louisiana	All	12.2 (7.6)	7.3 (94)	7.7 (102)	20.2 (12.6)	11.0 (87)	13.1 (104)
Tallahassee, Florida	All	9.1 (5.7)	5.5 (98)	5.7 (101)	28. 3 (17.7)	18.3 (103)	17.5 (99)
Manhattan, New York	All	(3.0)	2.4 (79)	3.1 (104)	12.5 (7.8)	8.1 (104)	7.6 (98)
Jacksonville, Florida	All	16.6 (10.4)	8.9 (85)	10.7 (103)	29.3 (18.3)	16.5 (90)	18.7 (102)
Miami, Florida	All	11.5 (7.2)	7.2 (100)	7.2 (100)	26.4 (16.5)	16.5 (101)	16.5 (100)
San Bernardino, California	All	11.8 (7.4)	7.7 (105)	7.4 (99)	33.9 (21.2)	21.3 (100)	21.2 (100)
Buffalo, New York	All	13.0 (8.1)	5.5 (69)	8.4 (104)	22.2 (13.9)	7.2 (53)	14.7 (106)
Huntsville, Alabama	All	11.4 (7.1)	5.3 (76)	7.3 (103)	25.3 (15.8)	12.5 (81)	16.2 (102)
All	Cardiology	12.6 (7.9)	7.0 (91)	8.0 (101)	24.6 (15.4)	13.8 (89)	15.7 (102)
All	Pulmonary	10.1 (6.3)	5.7 (92)	6.4 (102)	24.3 (15.2)	13.9 (91)	15.5 (103)
All	Gastroenterology	10.6 (6.6)	6.2 (94)	6.6 (101)	22.7 (14.2)	13.8 (94)	14.3 (102)
All	Orthopedics	12.6 (7.9)	7.2 (91)	8.0 (101)	23.8 (14.9)	13.2 (89)	15.2 (101)
All	General surgery	10.4 (6.5)	5.9 (92)	6.6 (101)	21.8 (13.6)	13.9 (104)	13.4 (98)
All	Neurology	12.0 (7.5)	6.7 (89)	7.7 (102)	25.4 (15.9)	13.2 (83)	16.5 (104)

Abbreviation: PCP, primary care physician.

^a^ Distance is measured from zip code centroids.

^b^ Black and White refer to the percentage of the overall measure present in the networks constructed using Black or White patients only.

**Figure 2.**
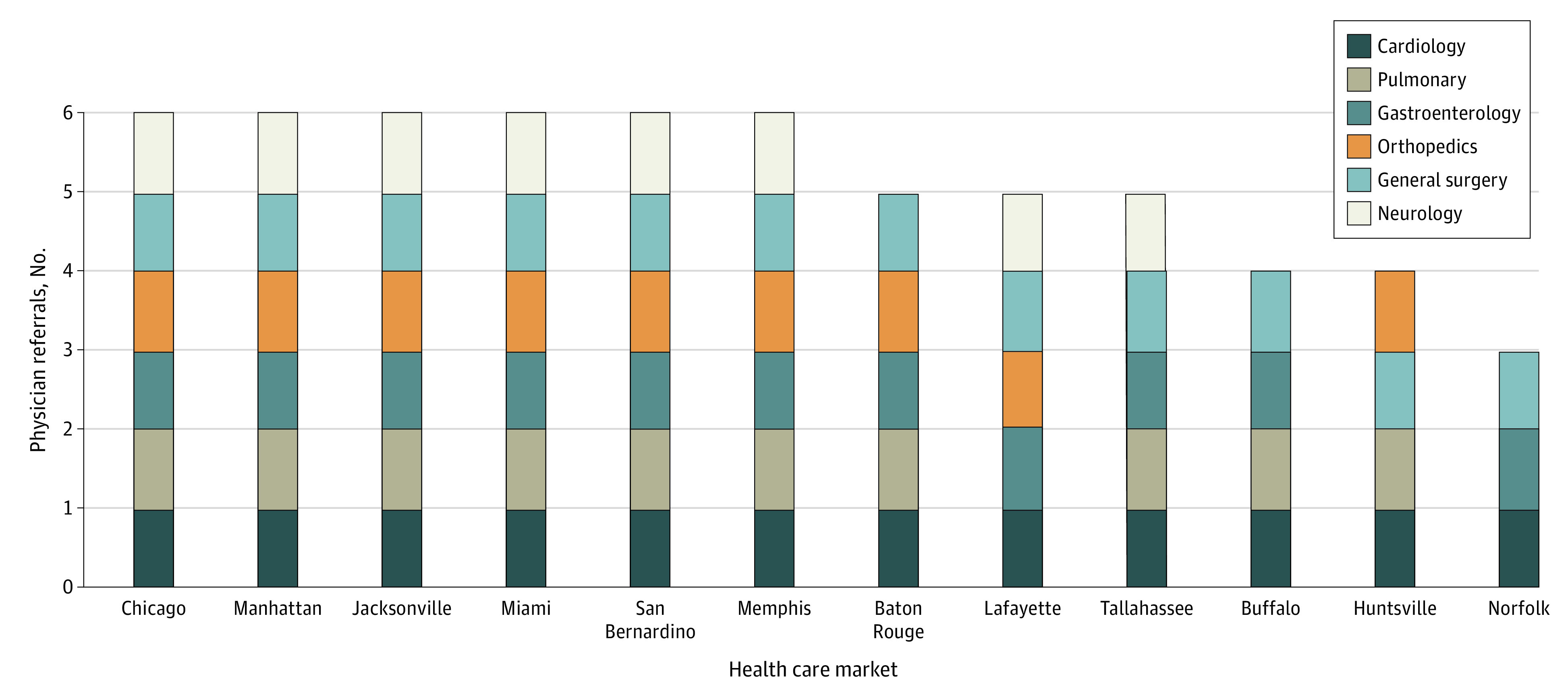
Overall Test for Referral Bias by Specialty and Market Results of the referral bias test by market and specialty. The different colored sections of each bar indicate that the test was statistically significant at *P* < .05.

Finally, the overall test for differences in referral patterns, which aggregates individual physician-level referral decisions across all individual physicians, was statistically significant for all 6 specialties examined in 7 of the 12 markets that we examined and in 5 specialties for another 3 markets (Figure 2). The referral bias test was least often statistically significant for pulmonary and neurology, the 2 specialties with the fewest numbers of specialists available. It was statistically significant in all markets for general surgery and cardiology.

## Discussion

In this study of physician referral patterns, we used techniques from the fields of social network analysis and network science to examine whether referrals to 6 common specialties differed by patient race. In examining referral choices across these specialties, we found differential sorting of patients to physicians by race across all of the specialties and within all of the markets we examined. These findings provide additional evidence that physicians may make different treatment decisions for their Black and White patients.

There are a number of reasons why physicians might make different referral decisions for different types of patients. First, even within the context of care delivered by a specialty, the particular conditions requiring referrals might differ systematically by race within an individual physician’s practice. For instance, within the broad field of cardiology, if the mix of cases related to electrophysiology, ischemic heart disease, or congestive heart failure differed by race, it might have led to a PCP choosing different cardiologists for referral.^33,34,35,36^ Second, patients of different races may have different preferences related to geographical location or institutional affiliation that might have been associated with the choice of specialty physician, although, in general, we found that the distance from a patient’s zip code to the specialist or from a PCP’s zip code to the specialist was similar for Black and White patients. Black patients may rely more on public transportation, which might limit the locations where they could travel. Third, because Medicare does not require approval from a PCP to see a specialist, at least some of the “referrals” that we identified might have been self-directed by patients (eg, those who are selecting physicians on their own) rather than by the physician.

Finally, differential referral behavior may be associated with differential decision-making by physicians, which may be explicit or implicit. Because all physicians are considered “in network” for Medicare, it is unlikely that network affiliations were associated with physician choices, although network inclusion might be associated with referral decisions for patients with commercial insurance or Medicaid, and these patterns might spill over to affect decisions for Medicare beneficiaries.^37,38^ Or, it might be that some specialists are less willing to provide care for some types of patients. This could be because of bias on the part of individual physicians, or it could reflect general differences in insurance coverage (eg, dual Medicare and Medicaid status) or other factors. We found that the networks of specialist physicians for Black patients was significantly smaller than the networks for White patients, even after adjusting for the number of Medicare beneficiaries seen by physicians. This finding suggests that referrals for Black patients come from fewer PCPs in general for any particular specialist physician.

Few studies have documented differences in patient management by race, to our knowledge. Those that have done so have used vignettes or other study methods that might not be reflective of real-world decision-making. For instance, Schulman and colleagues^18^ described an experiment in 1999 in which physicians were shown videos of actors posing as patients and reciting scripted interviews. They found that physicians’ recommendations for managing chest pain were associated with the race and sex of the actors. A similar study in 2007 found that physicians who take implicit association tests have implicit biases in favor White patients over Black patients. Moreover, physicians were more likely to negatively stereotype Black patients.^39^ Our study extends this work by examining actual patient-sharing practices among physicians. Our findings raise concerns about racial bias.

### Limitations

Our study has several limitations. First, we used claims data and were unable to fully characterize the nature and reasons for referrals to specialists.^40^ Second, as already noted, patients are not required to obtain a referral to a specialist in Medicare, so many of these specialist choices might have been made without the input of a PCP. However, one of the purposes of having a PCP is to obtain such recommendations, even when official referrals are not required. Third, the Black and White patients that we studied might have had different preferences or been concentrated in specific neighborhoods, both of which might be associated with choice of specialist. We did, however, examine distance traveled, which did show that Black patients, on average, traveled shorter distances to specialist physicians. Fourth, we did not include nurse practitioners, who often work as primary care clinicians, in our study. Thus, our results apply only to physician PCPs. Fifth, we focused on Medicare beneficiaries who were Black or White; sample sizes were too small to study Hispanic patients. Sixth, our study is observational, so causality cannot be inferred. Thus, this study should be considered hypothesis generating.

## Conclusions

In this study of patient sharing among PCPs and specialist physicians treating Black and White patients, we found evidence of differential choice of specialist by race. The reasons for these differences are likely to be multifactorial, and additional work is needed to understand the mechanisms of these associations. Nevertheless, our study provides evidence of differences in treatment patterns by race among patients insured by traditional Medicare, even after a patient has selected a PCP.

## Notes

Supplement. **eTable 1.** Network Measures by Market and Specialty Combined After Sampling White Patients **eTable 2.** Distance Measures by Market and Specialty Combined After Sampling White Patients **eFigure 1.** Distribution of States With Included Markets **eFigure 2.** Physician Networks Visualized by Patients’ Race
