# Supplementary material for: Assessment of Racial Disparities in Primary Care Physician Specialty Referrals
Source: JAMA Netw Open. 2021 Jan 25;4(1):e2029238. doi: 10.1001/jamanetworkopen.2020.29238 (PMC7835717; doi:10.1001/jamanetworkopen.2020.29238)
Supplement: Supplement. — eTable 1. Network Measures by Market and Specialty Combined After Sampling White Patients eTable 2. Distance Measures by Market and Specialty Combined After Sampling White Patients eFigure 1. Distribution of States With Included Markets eFigure 2. Physician Networks Visualized by Patients’ Race [file jamanetwopen-e2029238-s001.pdf]

Supplementary Online Content

Landon BE, Onnela JP, Meneades L, O'Malley AJ, Keating NL. Assessment of racial disparities in primary care physician specialty referrals. *JAMA Netw Open.* 2020;3(12):e2029238. doi:10.1001/jamanetworkopen.2020.29238

eTable 1. Network Measures by Market and Specialty Combined After Sampling White Patients
eTable 2. Distance Measures by Market and Specialty Combined After Sampling White Patients
eFigure 1. Distribution of States With Included Markets
eFigure 2. Physician Networks Visualized by Patients' Race

This supplementary material has been provided by the authors to give readers additional information about their work.

© 2021 Landon BE et al. *JAMA Network Open.*

eTable 1. Network Measures by Market and Specialty Combined After Sampling White Patients

Market	SPEC	PCP Degree All	Black *	White*	Specialist Degree All	Black*	White*	PCP Strength	Black*	White*
Chicago IL	ALL	5.6	66%	73%	145.6	59%	60%	1.6	86%	88%
Memphis TN	ALL	9.6	67%	74%	270.1	52%	74%	2.9	78%	80%
Baton Rouge LA	ALL	8.0	67%	76%	200.0	54%	78%	2.6	76%	78%
Norfolk VA	ALL	9.5	67%	73%	301.6	51%	78%	2.6	77%	79%
Lafayette LA	ALL	5.7	68%	77%	184.5	52%	84%	3.1	72%	78%
Tallahassee FL	ALL	4.8	75%	80%	133.6	56%	83%	3.6	70%	72%
Manhattan NY	ALL	4.6	67%	75%	145.4	28%	84%	1.5	90%	88%
Jacksonville FL	ALL	8.2	67%	73%	253.9	41%	83%	2.3	78%	82%
Miami FL	ALL	6.7	66%	72%	204.1	33%	82%	1.7	85%	88%
San Bernardino CA	ALL	3.4	76%	74%	117.2	36%	83%	1.8	82%	85%
Buffalo NY	ALL	4.6	67%	75%	138.7	30%	84%	1.7	86%	85%
Huntsville AL	ALL	4.9	72%	76%	189.2	40%	87%	1.8	81%	82%
ALL	CARDIO	12.4	64%	73%	261.0	47%	81%	2.7	77%	79%
ALL	PULMN	5.6	73%	78%	262.2	51%	81%	2.9	74%	79%
ALL	GASTRO	5.3	70%	76%	195.1	45%	80%	2.3	80%	80%
ALL	ORTHO	4.8	63%	76%	102.7	34%	83%	1.6	85%	89%
ALL	GENSURG	4.5	73%	71%	115.6	46%	76%	2.0	83%	82%
ALL	NEURO	5.1	70%	74%	205.4	43%	80%	2.1	81%	82%

* Black and White refer to the percentage of the overall measure present in the networks constructed using Black or White patients only. Degree is the number of ties between PCPs and specialists. Strength is the number of shared patients. All represents the average across physicians in each of the six specialties.

eTable 2. Distance Measures by Market and Specialty Combined After Sampling White Patients

Market	SPEC	PCP to Specialist Distance	Black*	White*	Patient to Specialist Distance	Black*	White*
Chicago IL	ALL	2.4	107%	94%	10.6	97%	103%
Memphis TN	ALL	8.8	98%	101%	17.4	95%	104%
Baton Rouge LA	ALL	6.7	100%	100%	13.7	98%	102%
Norfolk VA	ALL	9.2	99%	101%	15.0	95%	105%
Lafayette LA	ALL	7.8	96%	103%	12.7	90%	107%
Tallahassee FL	ALL	6.1	95%	105%	18.8	98%	101%
Manhattan NY	ALL	2.4	97%	102%	8.1	102%	98%
Jacksonville FL	ALL	9.7	98%	102%	17.8	96%	103%
Miami FL	ALL	7.1	102%	98%	17.4	100%	99%
San Bernardino CA	ALL	7.7	101%	99%	21.9	97%	102%
Buffalo NY	ALL	6.0	95%	105%	12.9	59%	135%
Huntsville AL	ALL	5.7	96%	103%	14.7	88%	110%
ALL	CARDIO	7.3	99%	101%	15.6	92%	106%
ALL	PULMN	5.8	100%	100%	15.4	91%	107%
ALL	GASTRO	6.3	98%	102%	15.2	92%	107%
ALL	ORTHO	7.5	97%	102%	14.4	95%	104%
ALL	GENSURG	6.2	99%	100%	15.0	99%	100%
ALL	NEURO	6.8	99%	101%	15.0	89%	109%

* Black and White refer to the percentage of the overall measure present in the networks constructed using Black or White patients only. All represents the average across physicians in each of the six specialties.

© 2021 Landon BE et al. *JAMA Network Open.*

eFigure 1. Distribution of States With Included Markets

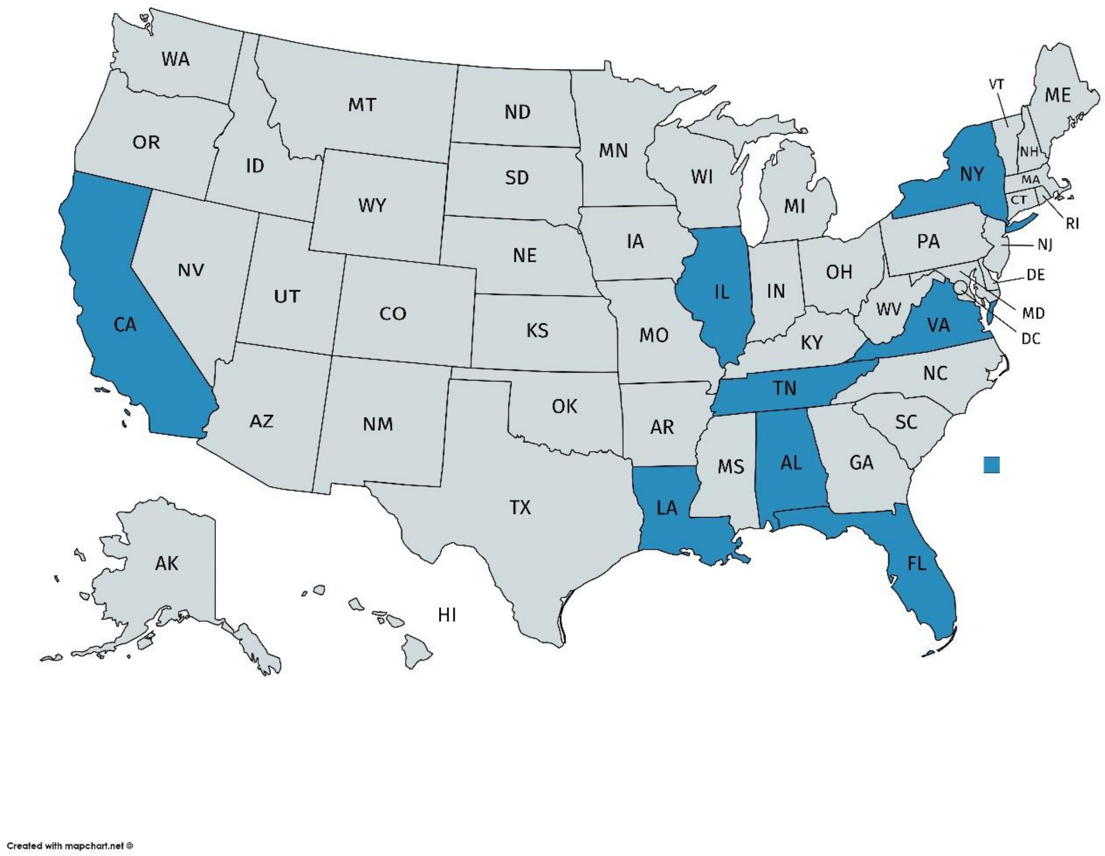

© 2021 Landon BE et al. *JAMA Network Open*.

eFigure 2. Physician Networks Visualized by Patients' Race

Hospital Referral Regions (1-12) are Buffalo, Chicago, Huntsville, Jacksonville, Lafayette, Lynchburg, Manhattan, Memphis, Miami, Norfolk, San Bernardino, and Tallahassee. Specialties (1-6) are cardiology, gastroenterology, general surgery, neurology, orthopedic surgery, and pulmonary disease.

© 2021 Landon BE et al. *JAMA Network Open.*

HRR: 1; Specialty: 1; Sample: 1

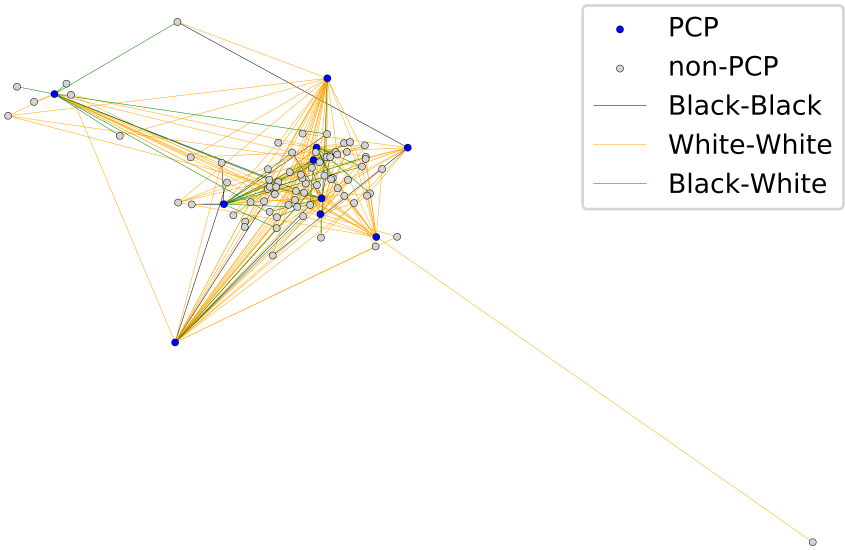

© 2021 Landon BE et al. JAMA Network Open.

HRR: 1; Specialty: 1; Sample: 2

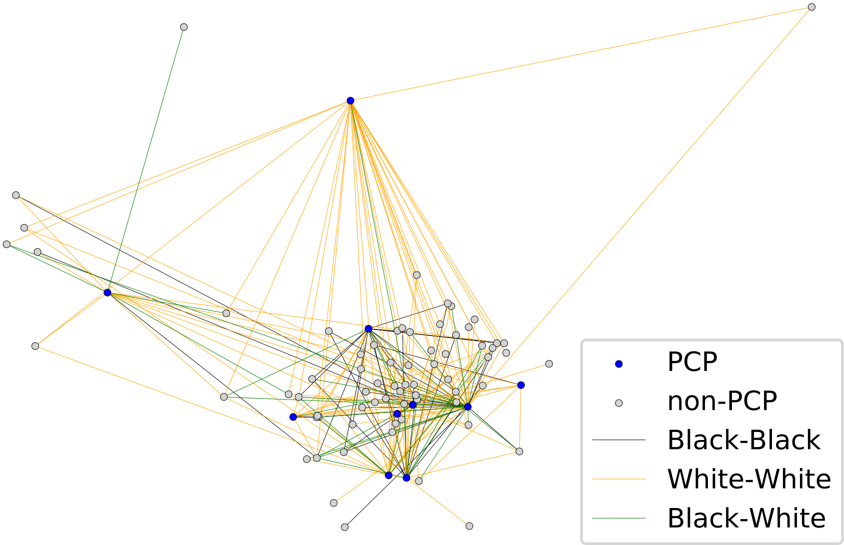

© 2021 Landon BE et al. JAMA Network Open.

HRR: 1; Specialty: 1; Sample: 3

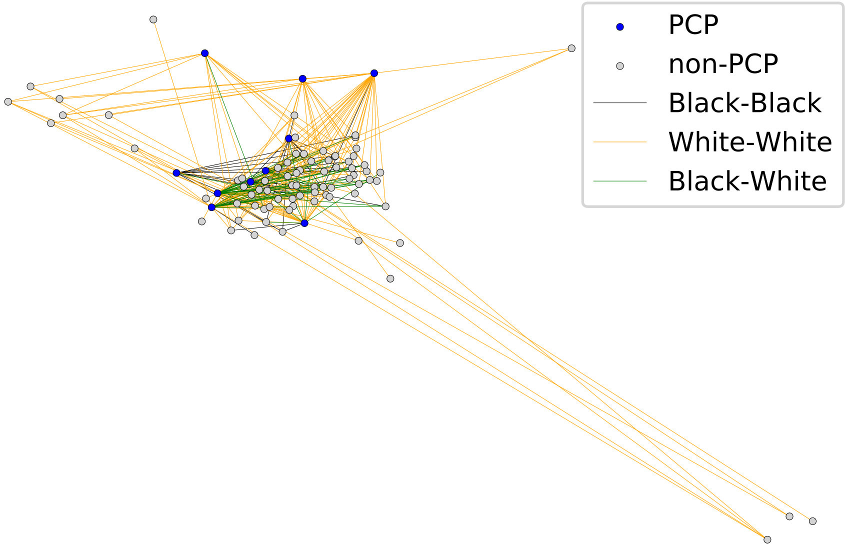

© 2021 Landon BE et al. JAMA Network Open.

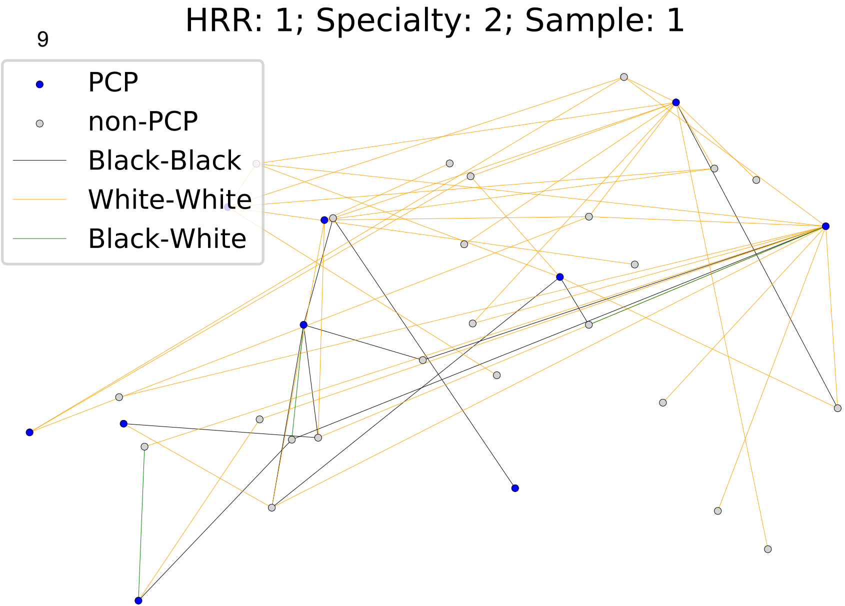

© 2021 Landon BE et al. JAMA Network Open.

HRR: 1; Specialty: 2; Sample: 2

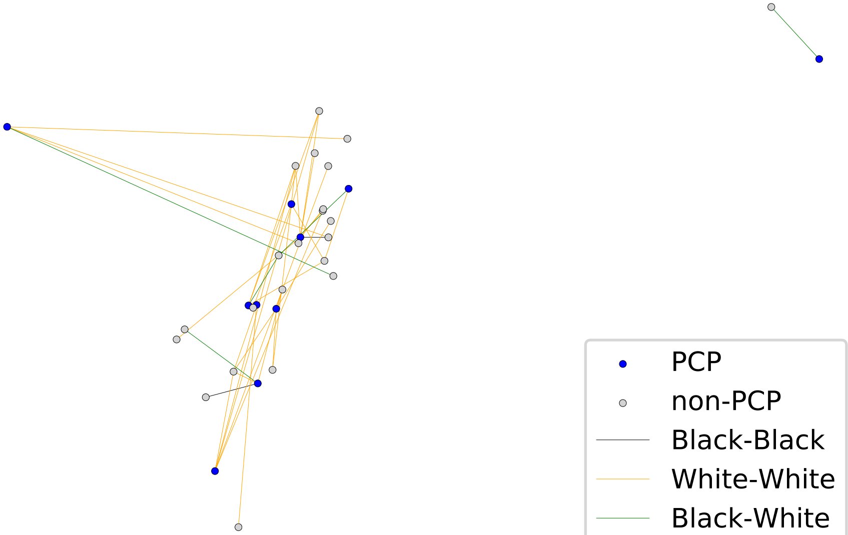

© 2021 Landon BE et al. JAMA Network Open.

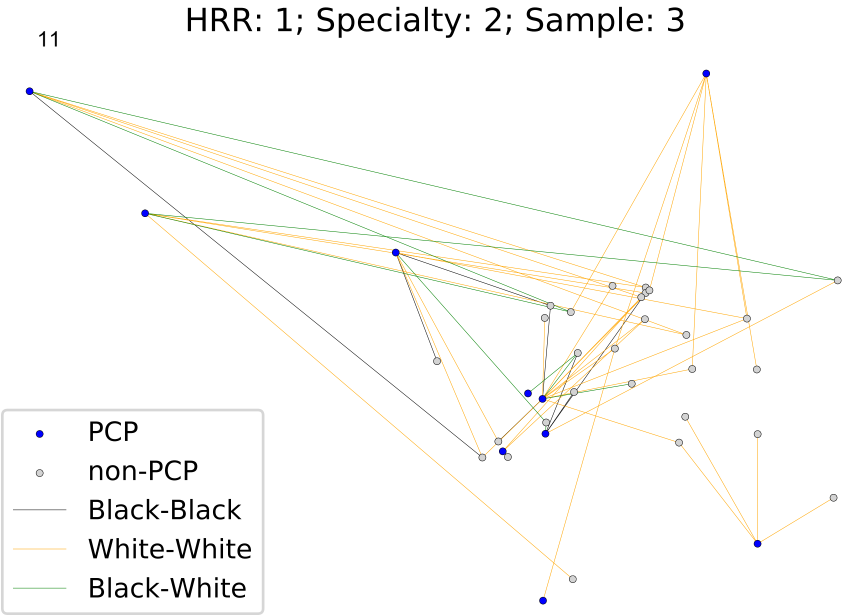

© 2021 Landon BE et al. JAMA Network Open.

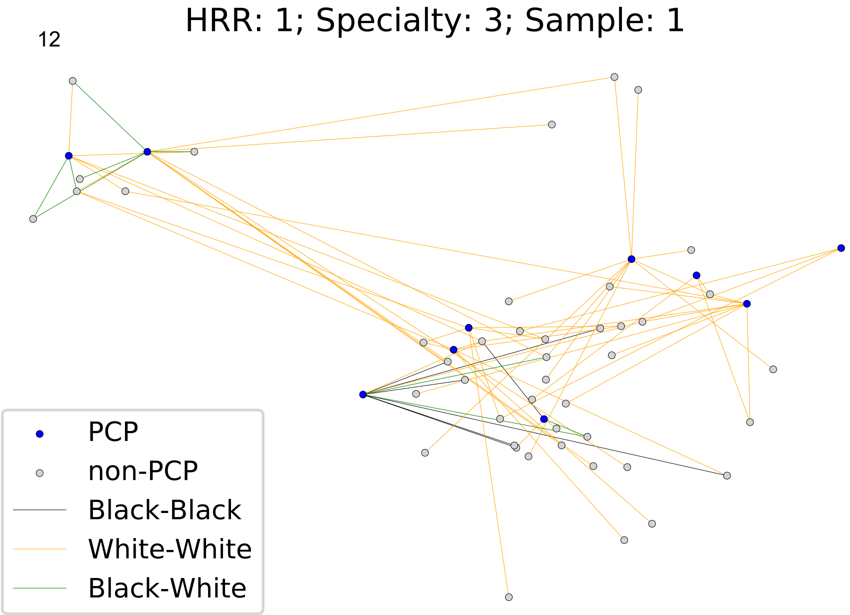

© 2021 Landon BE et al. JAMA Network Open.

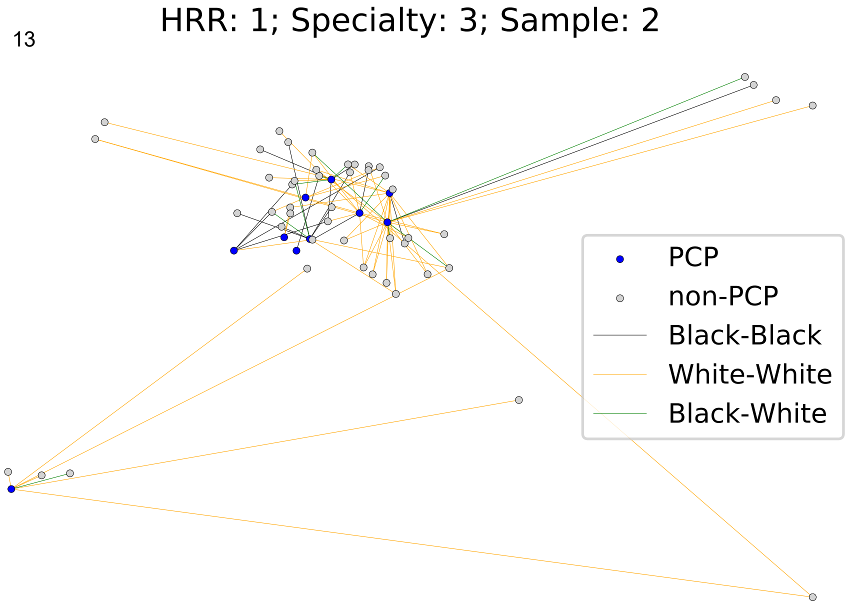

© 2021 Landon BE et al. JAMA Network Open.

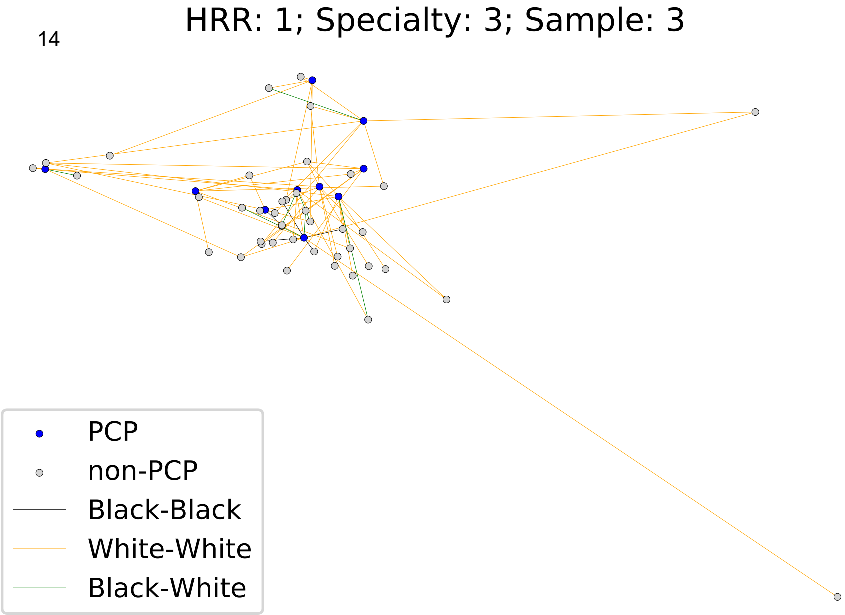

© 2021 Landon BE et al. JAMA Network Open.

HRR: 1; Specialty: 4; Sample: 1

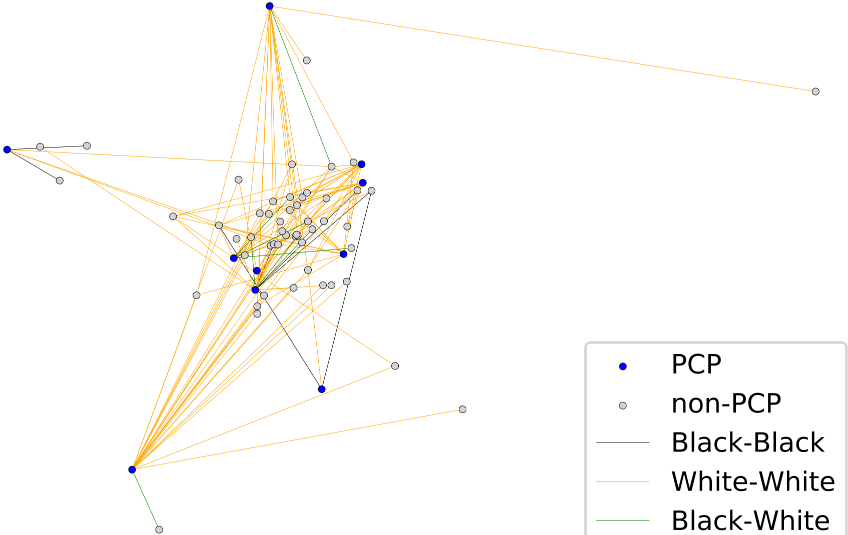

© 2021 Landon BE et al. JAMA Network Open.

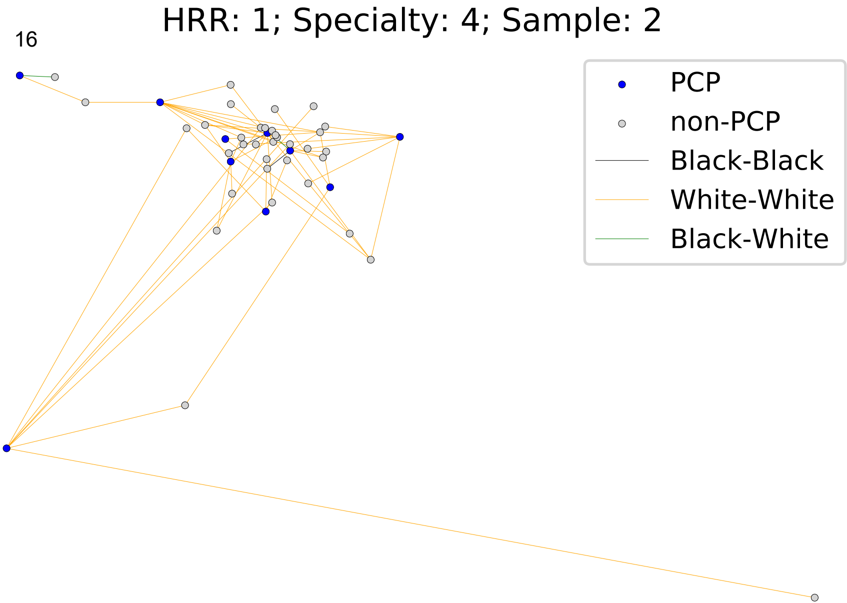

© 2021 Landon BE et al. JAMA Network Open.

HRR: 1; Specialty: 4; Sample: 3

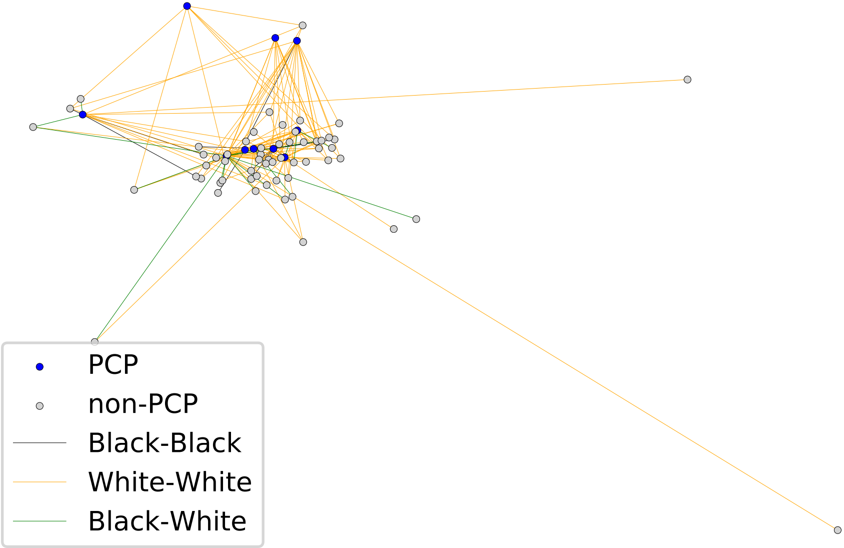

© 2021 Landon BE et al. JAMA Network Open.

HRR: 1; Specialty: 5; Sample: 1

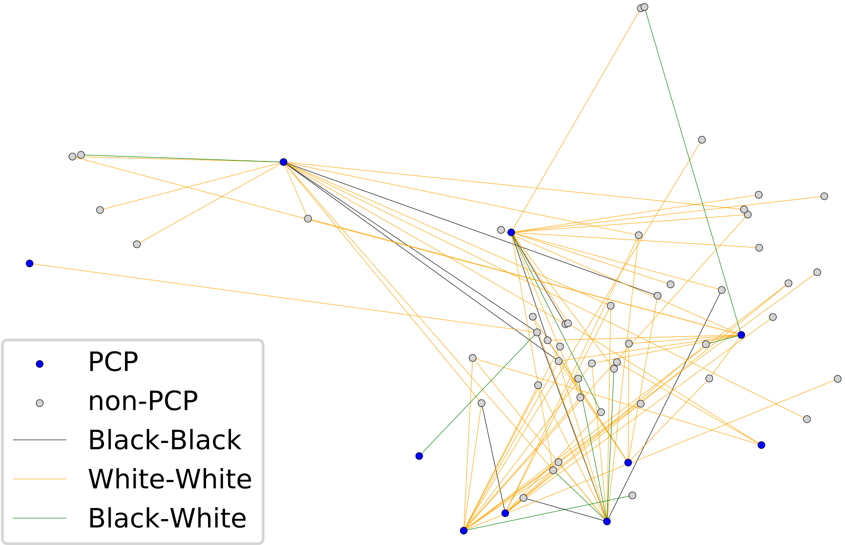

© 2021 Landon BE et al. JAMA Network Open.

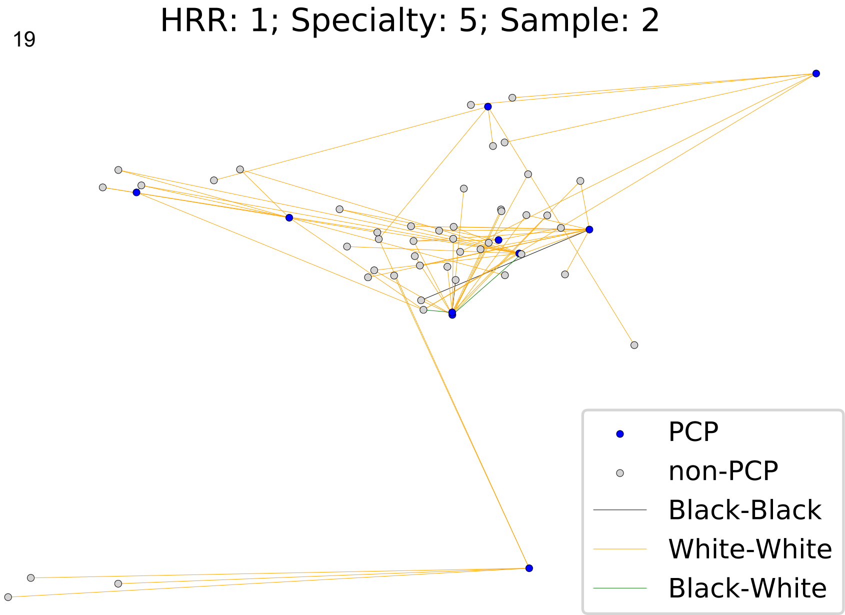

© 2021 Landon BE et al. JAMA Network Open.

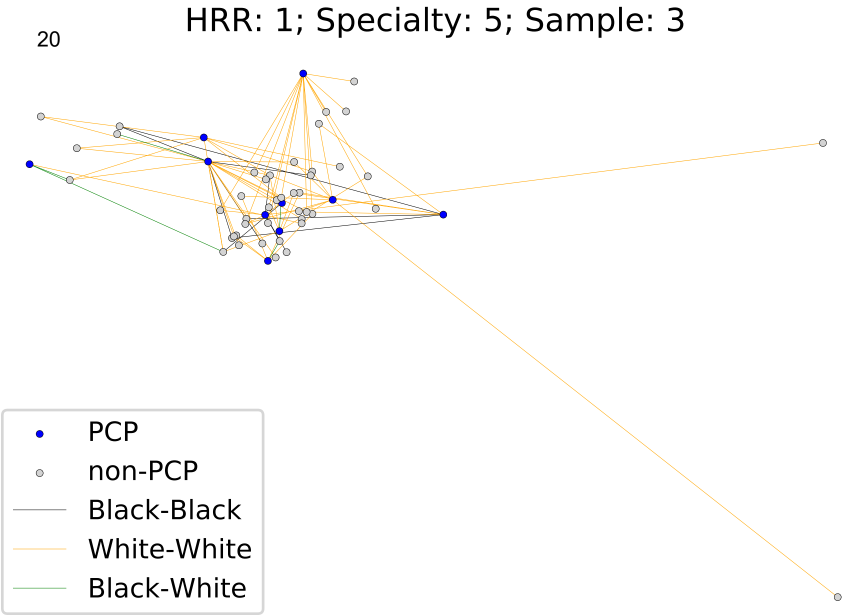

© 2021 Landon BE et al. JAMA Network Open.

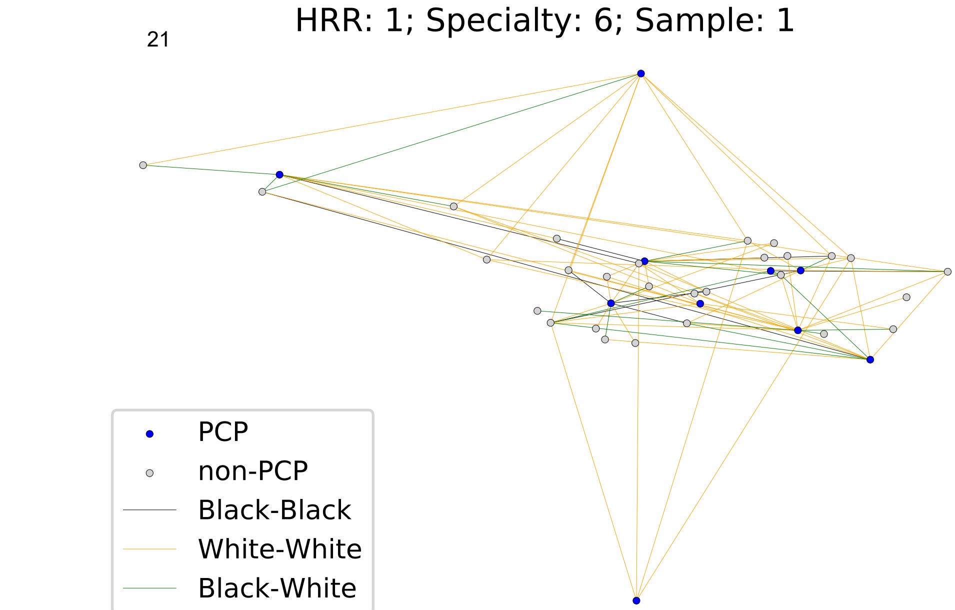

© 2021 Landon BE et al. JAMA Network Open.

HRR: 1; Specialty: 6; Sample: 2

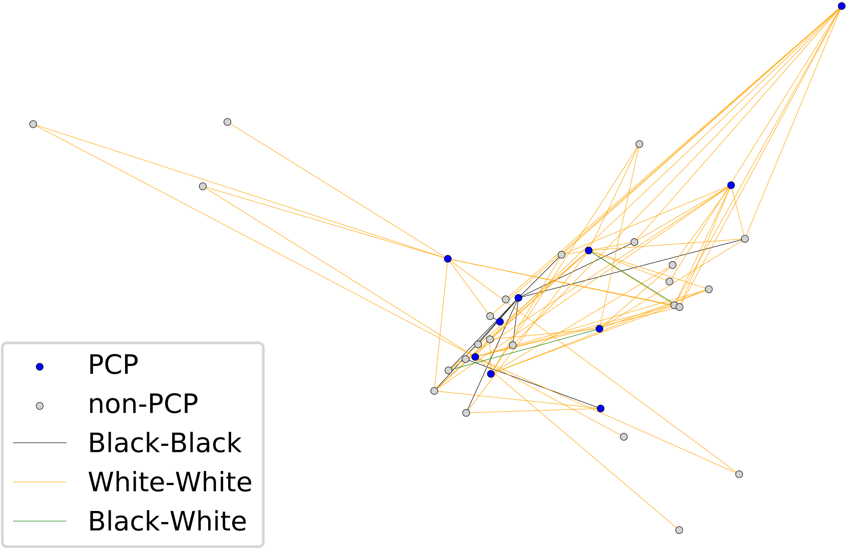

© 2021 Landon BE et al. JAMA Network Open.

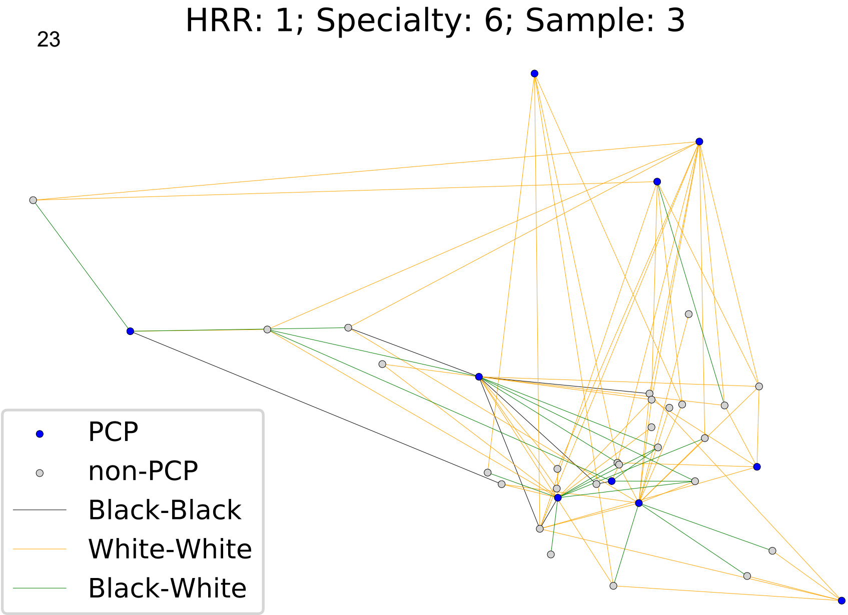

© 2021 Landon BE et al. JAMA Network Open.

HRR: 2; Specialty: 1; Sample: 1

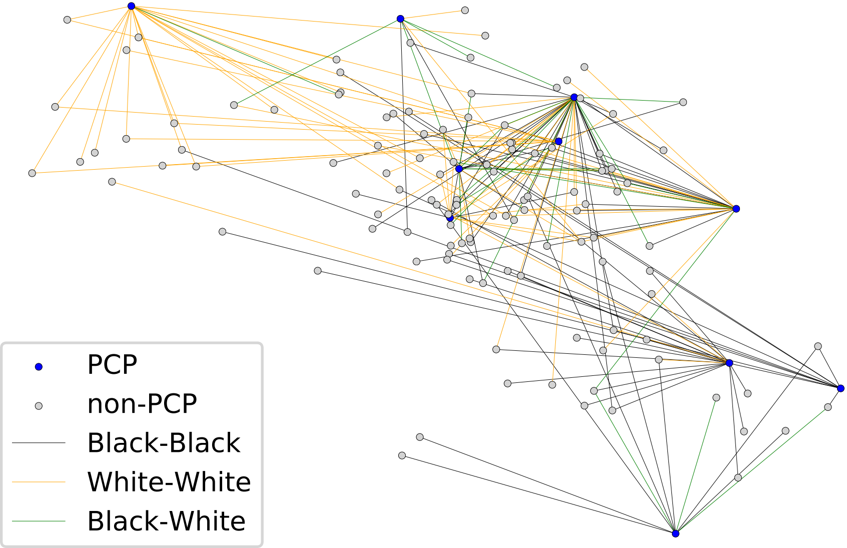

© 2021 Landon BE et al. JAMA Network Open.

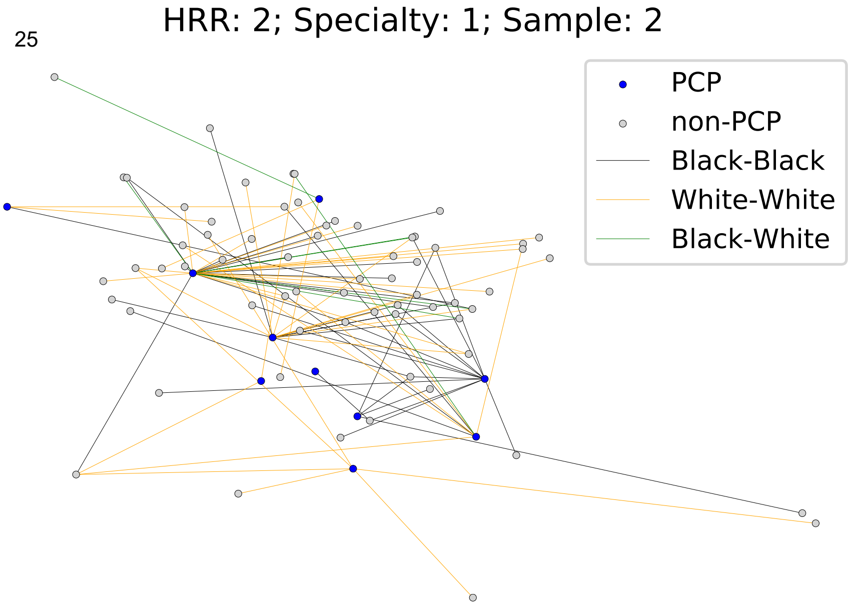

© 2021 Landon BE et al. JAMA Network Open.

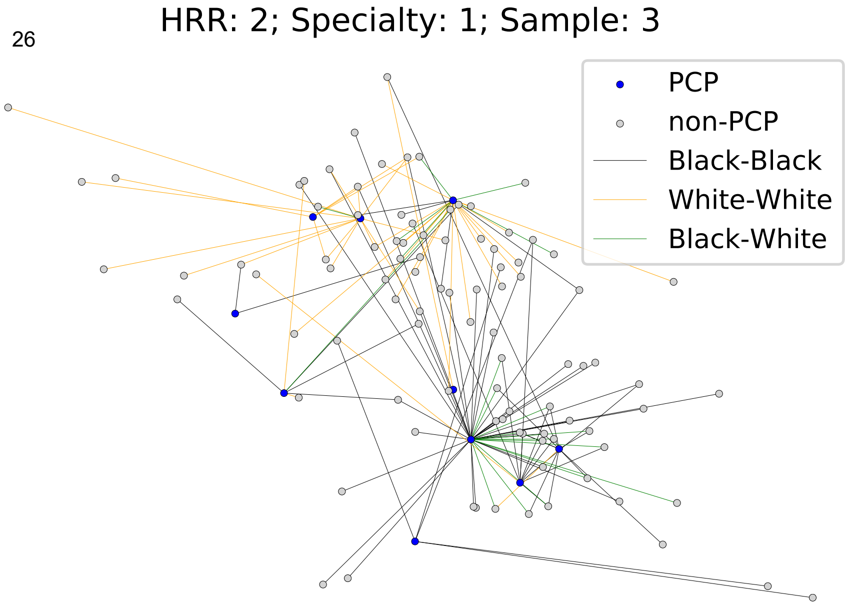

© 2021 Landon BE et al. JAMA Network Open.

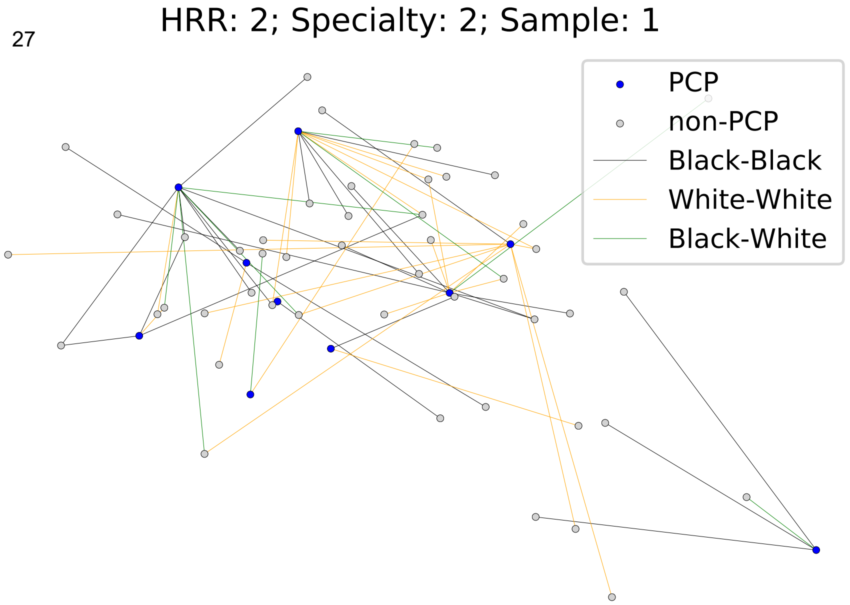

© 2021 Landon BE et al. JAMA Network Open.

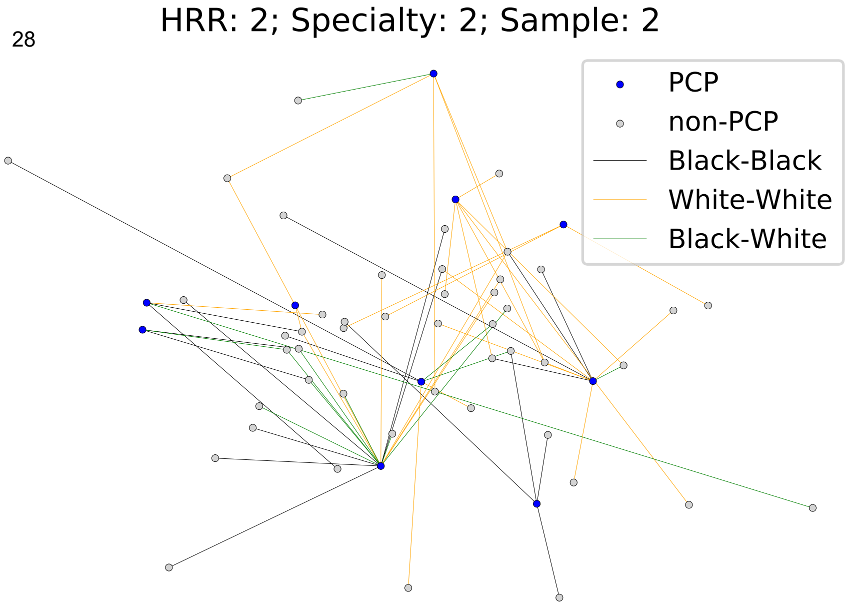

© 2021 Landon BE et al. JAMA Network Open.

HRR: 2; Specialty: 2; Sample: 3

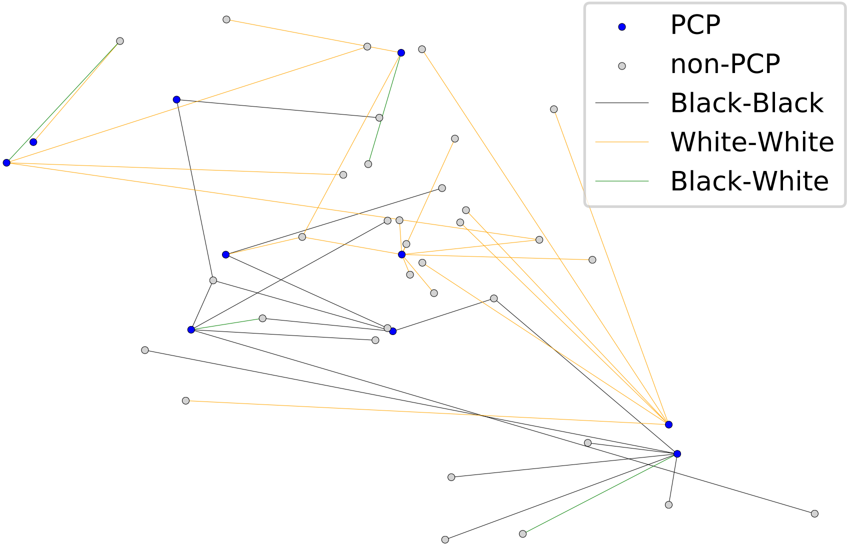

© 2021 Landon BE et al. JAMA Network Open.

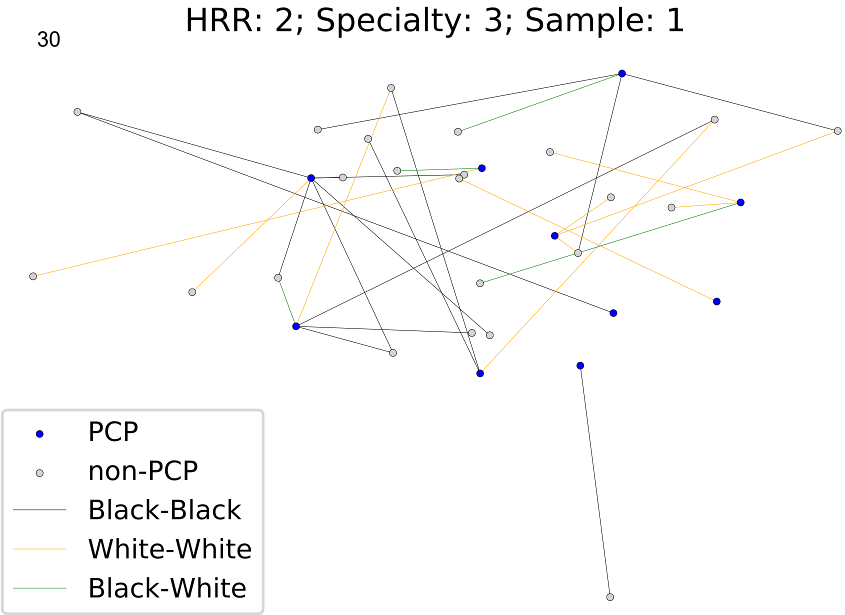

© 2021 Landon BE et al. JAMA Network Open.

HRR: 2; Specialty: 3; Sample: 2

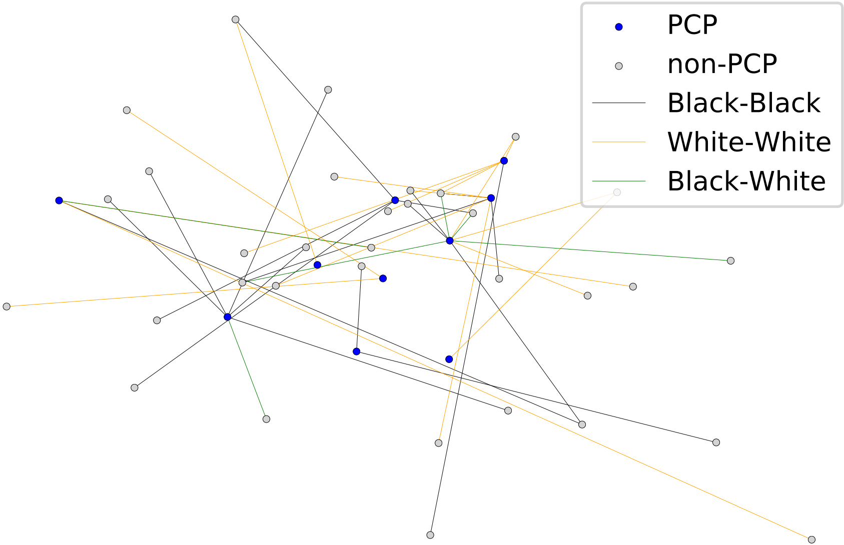

© 2021 Landon BE et al. JAMA Network Open.

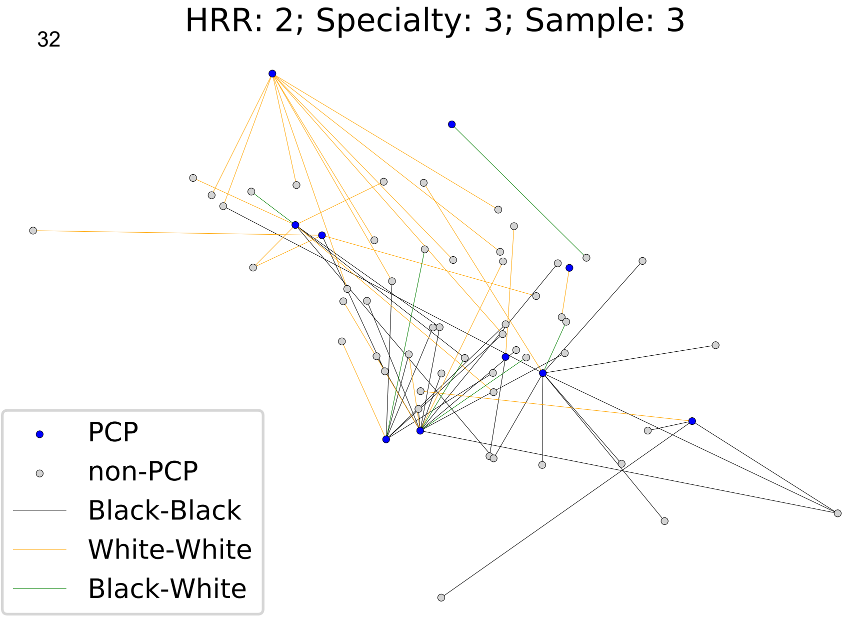

© 2021 Landon BE et al. JAMA Network Open.

HRR: 2; Specialty: 4; Sample: 1

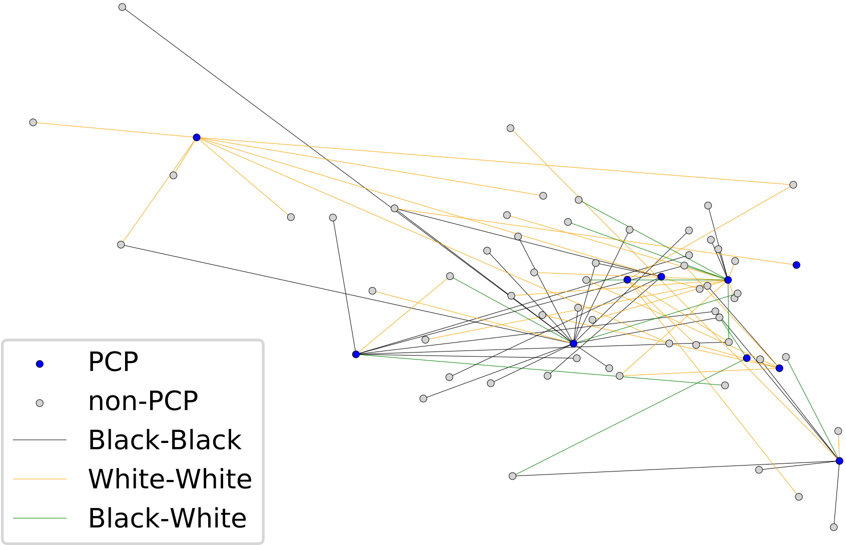

© 2021 Landon BE et al. JAMA Network Open.

HRR: 2; Specialty: 4; Sample: 2

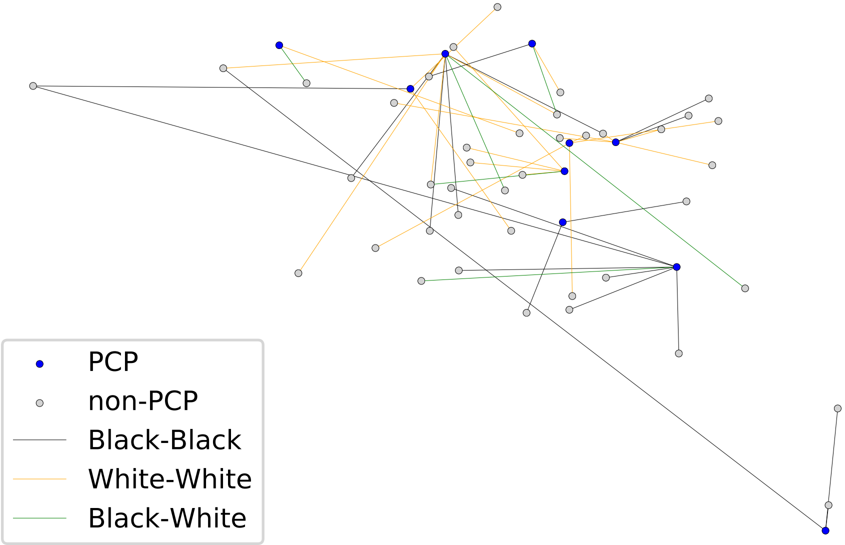

© 2021 Landon BE et al. JAMA Network Open.

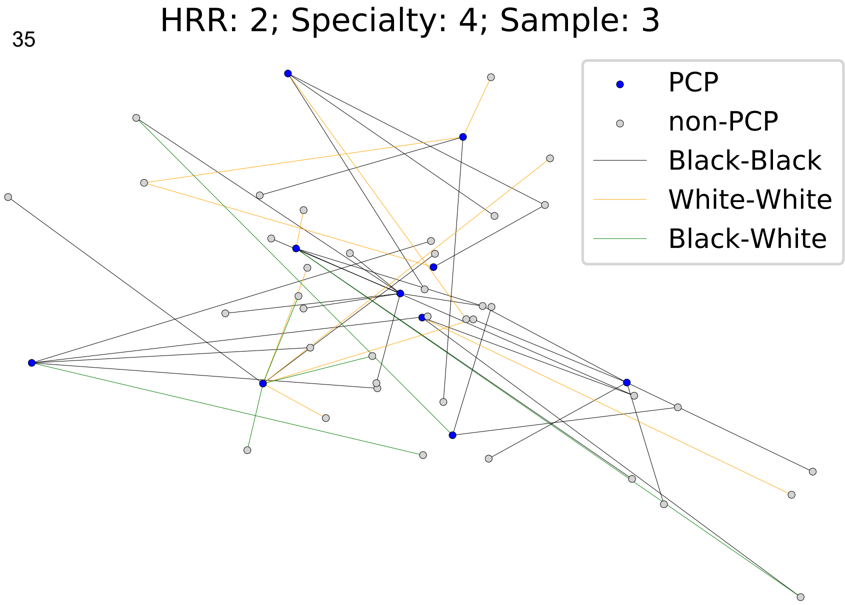

© 2021 Landon BE et al. JAMA Network Open.

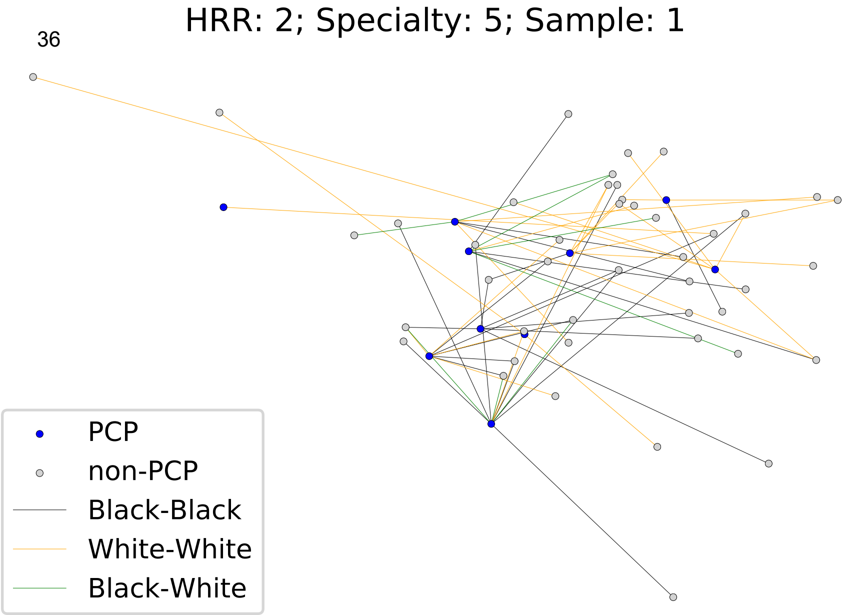

© 2021 Landon BE et al. JAMA Network Open.

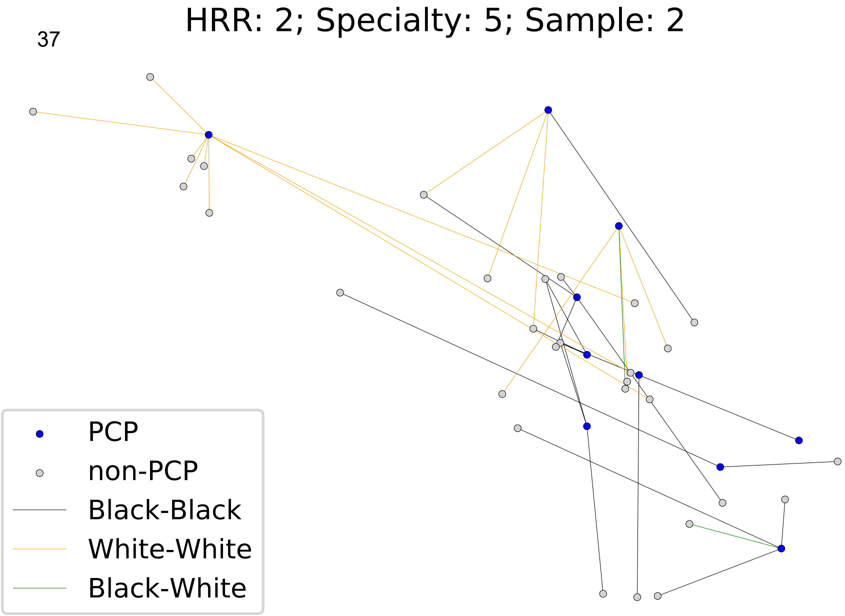

© 2021 Landon BE et al. JAMA Network Open.

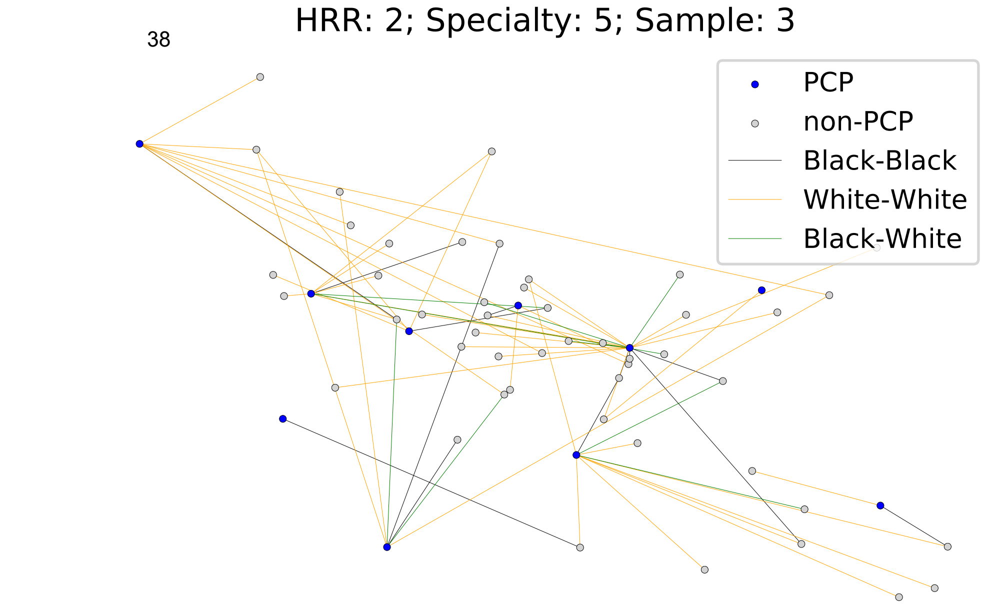

© 2021 Landon BE et al. JAMA Network Open.

HRR: 2; Specialty: 6; Sample: 1

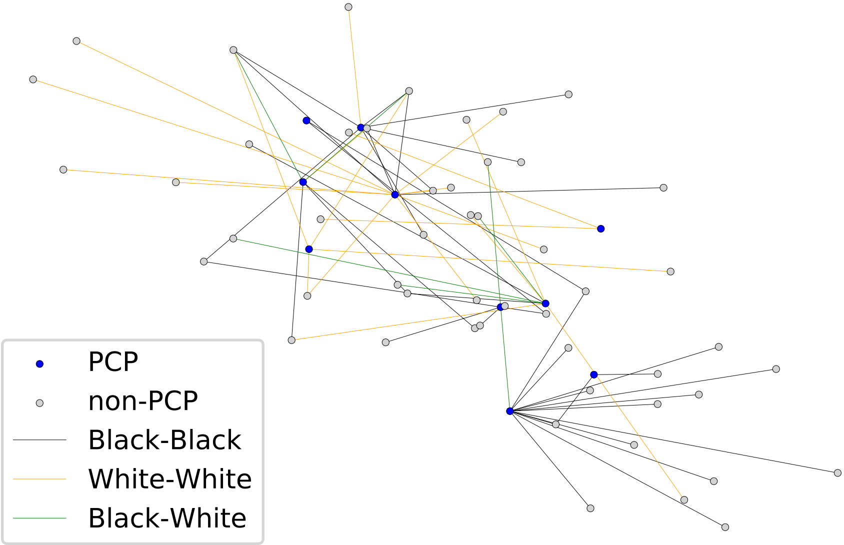

© 2021 Landon BE et al. JAMA Network Open.

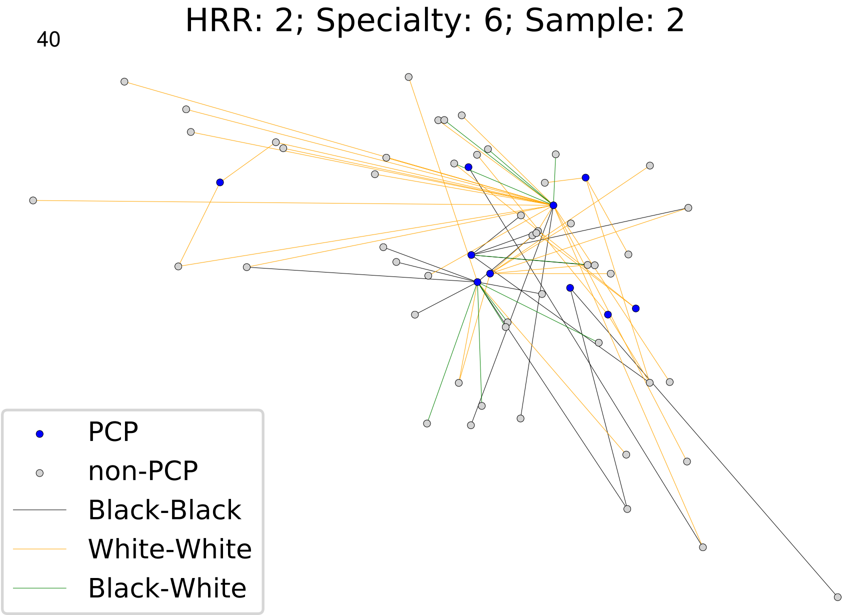

© 2021 Landon BE et al. JAMA Network Open.

HRR: 2; Specialty: 6; Sample: 3

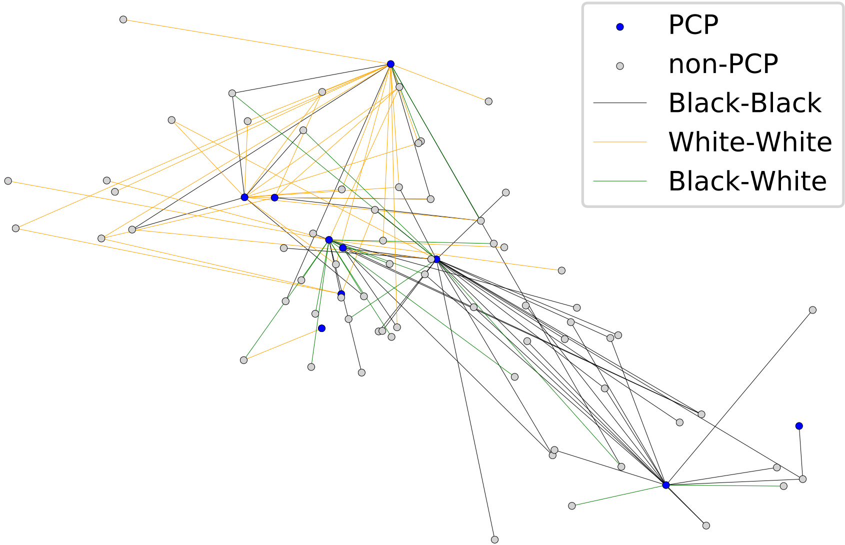

© 2021 Landon BE et al. JAMA Network Open.

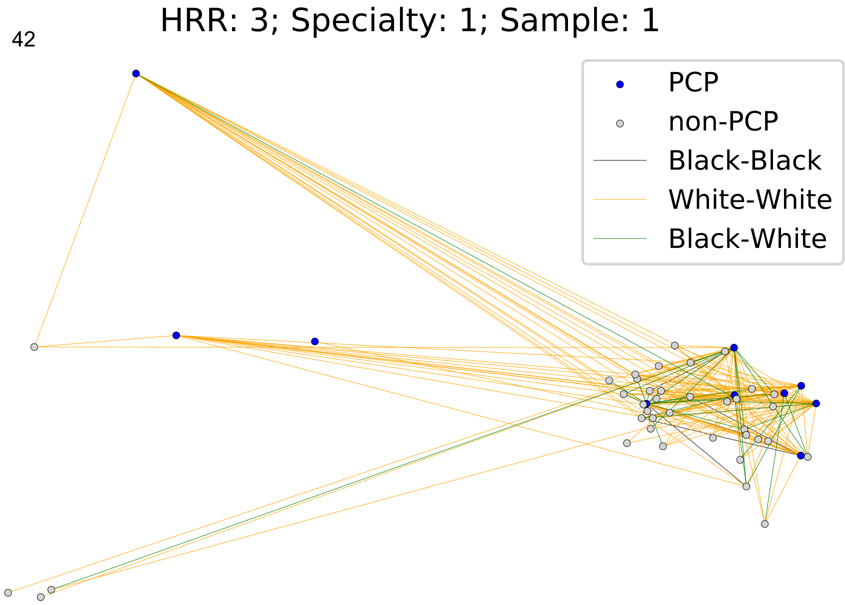

© 2021 Landon BE et al. JAMA Network Open.

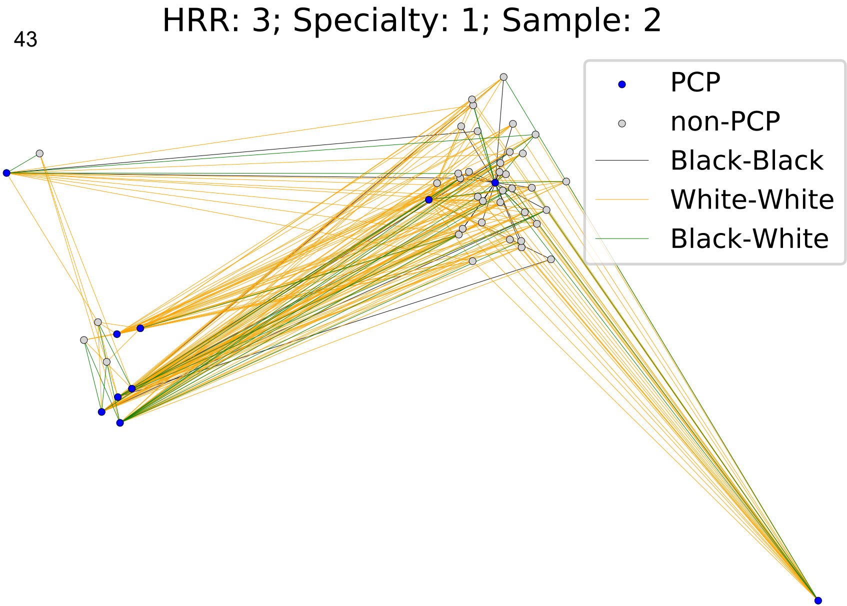

© 2021 Landon BE et al. JAMA Network Open.

HRR: 3; Specialty: 1; Sample: 3

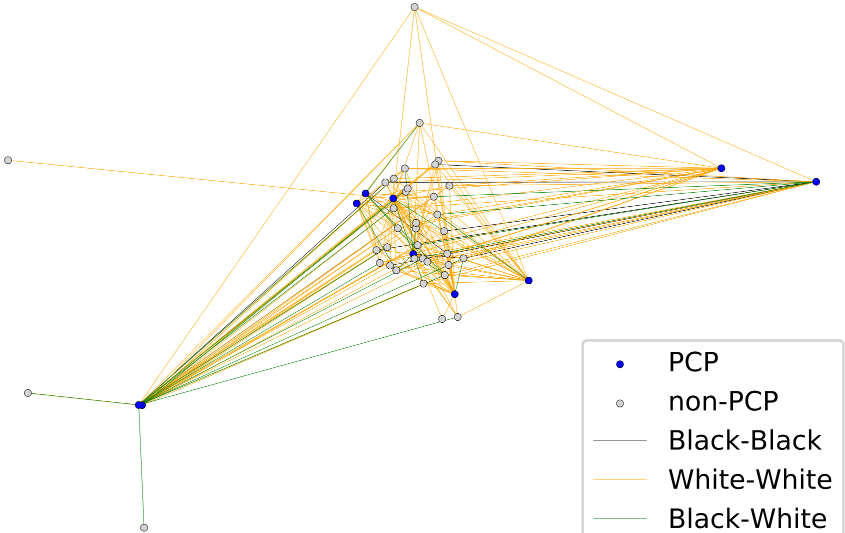

© 2021 Landon BE et al. JAMA Network Open.

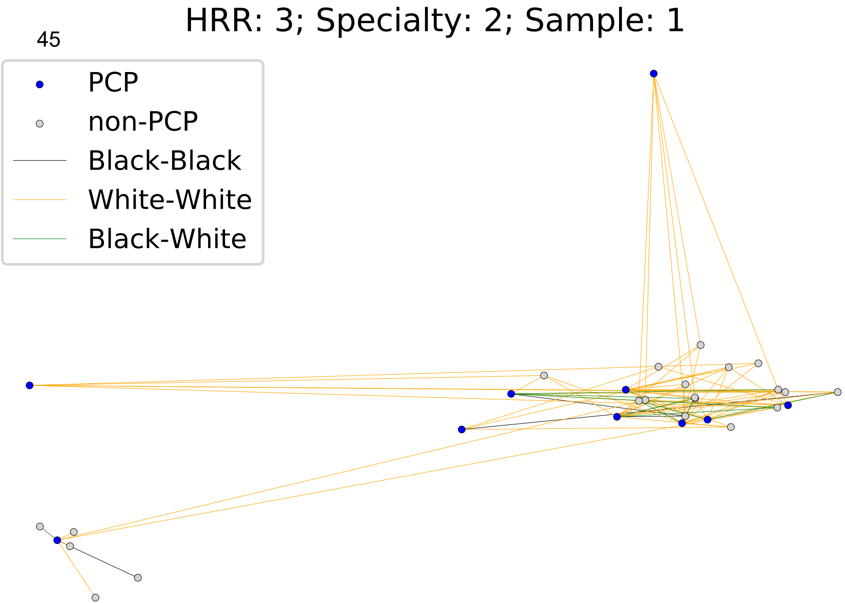

© 2021 Landon BE et al. JAMA Network Open.

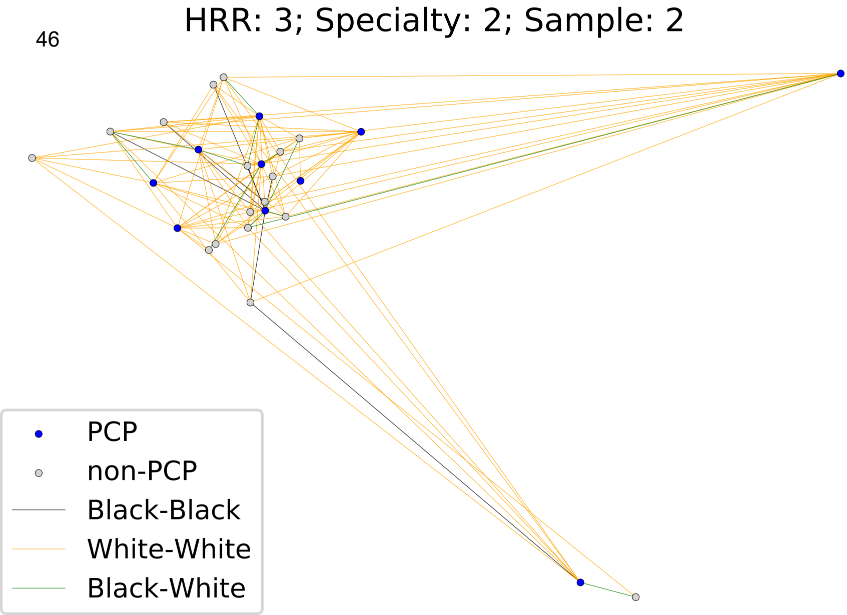

© 2021 Landon BE et al. JAMA Network Open.

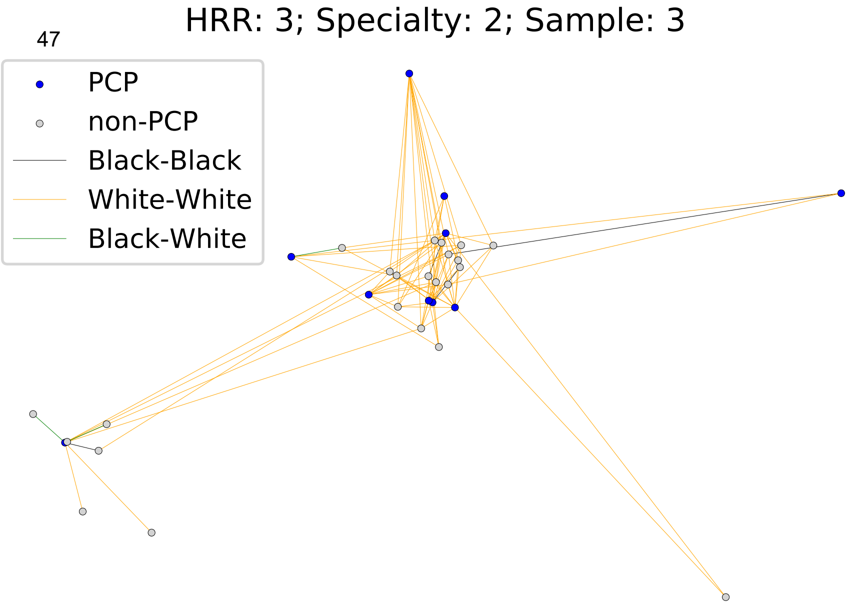

© 2021 Landon BE et al. JAMA Network Open.

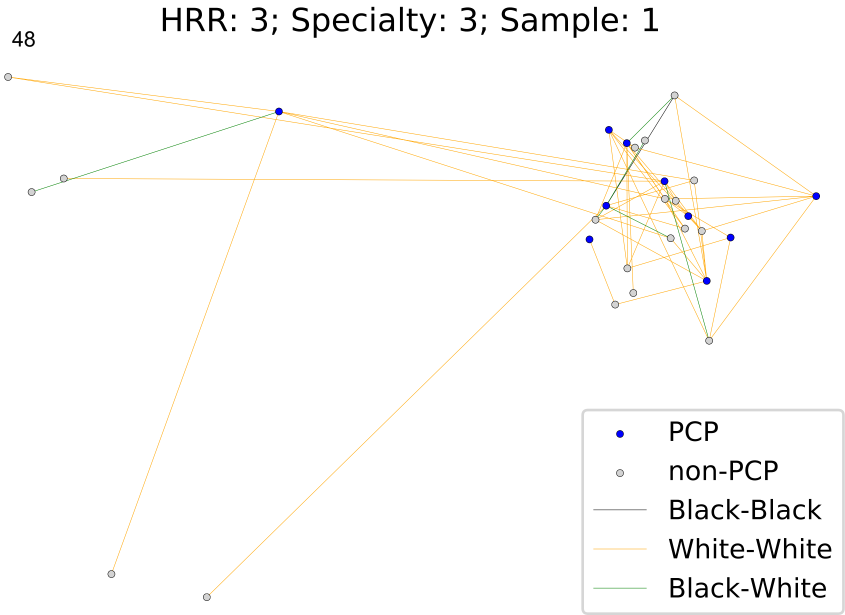

© 2021 Landon BE et al. JAMA Network Open.

HRR: 3; Specialty: 3; Sample: 2

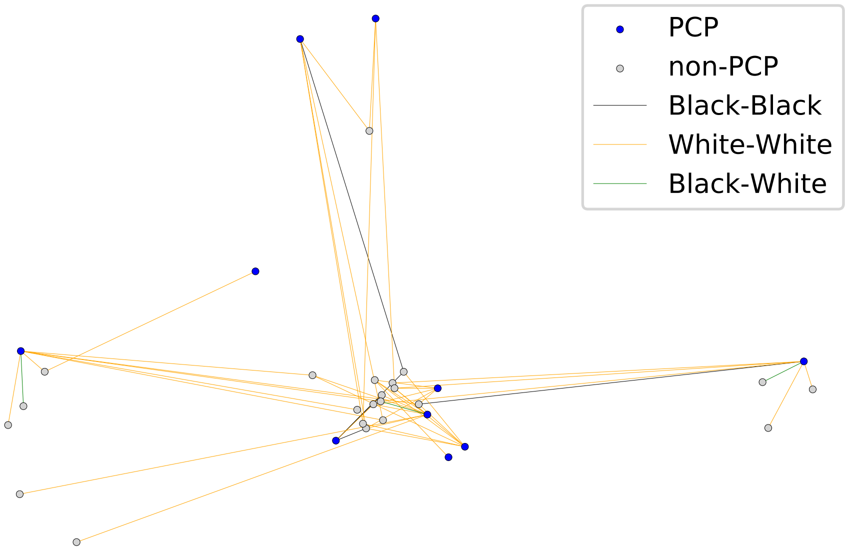

© 2021 Landon BE et al. JAMA Network Open.

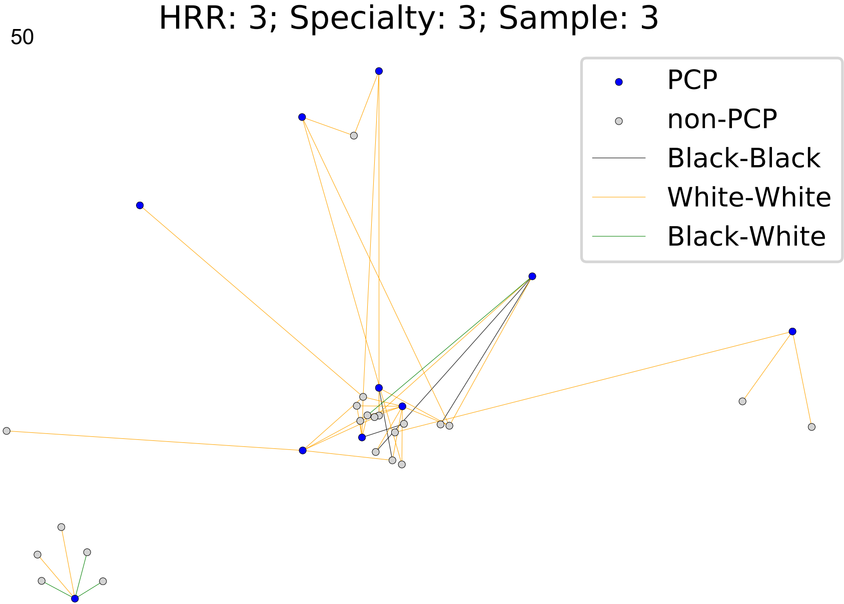

© 2021 Landon BE et al. JAMA Network Open.

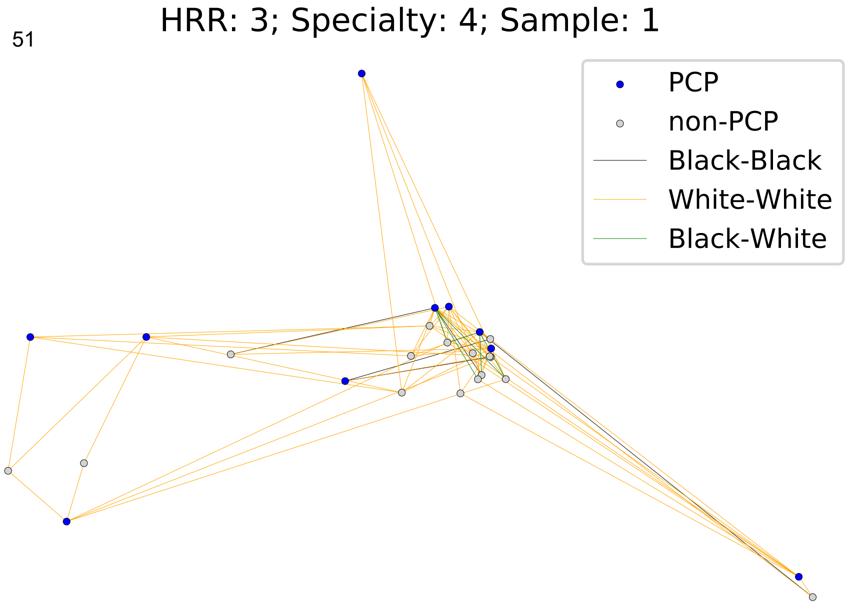

© 2021 Landon BE et al. JAMA Network Open.

52

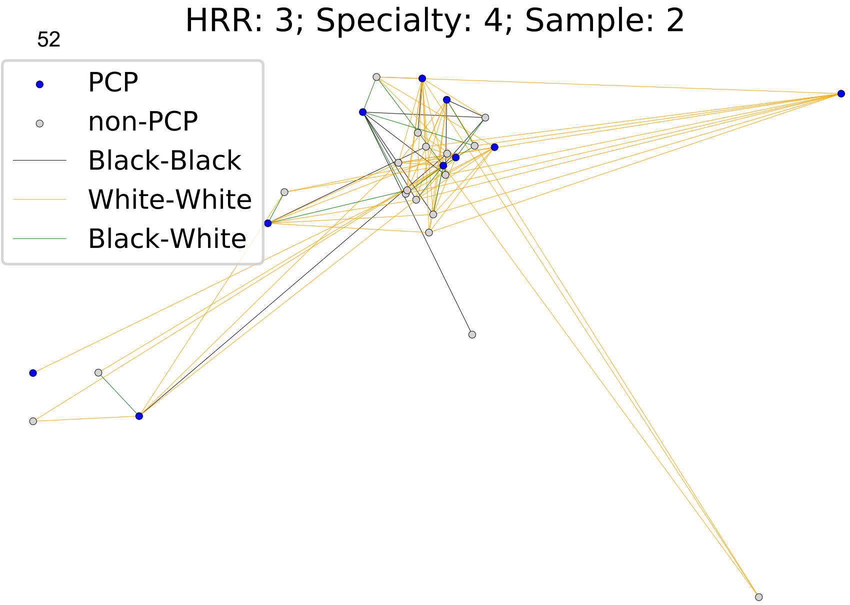

© 2021 Landon BE et al. JAMA Network Open.

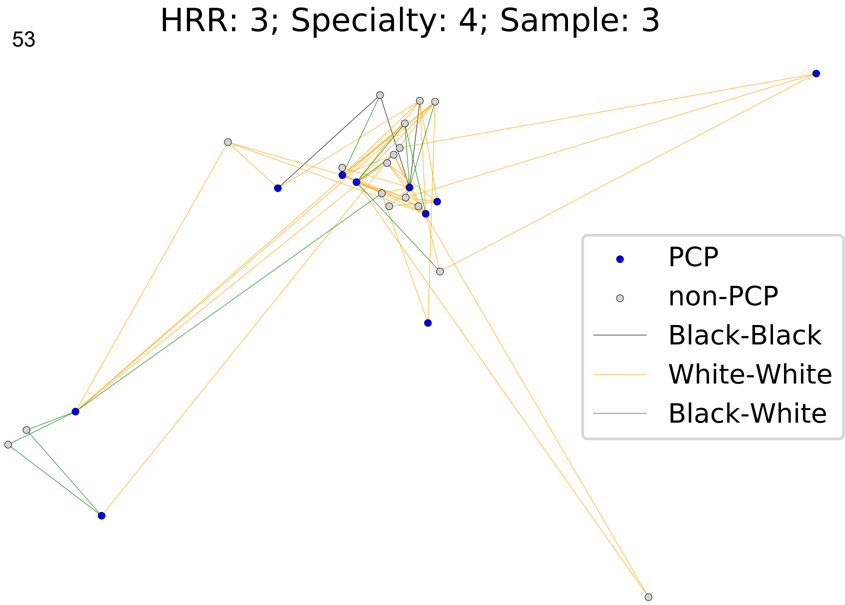

© 2021 Landon BE et al. JAMA Network Open.

HRR: 3; Specialty: 5; Sample: 1

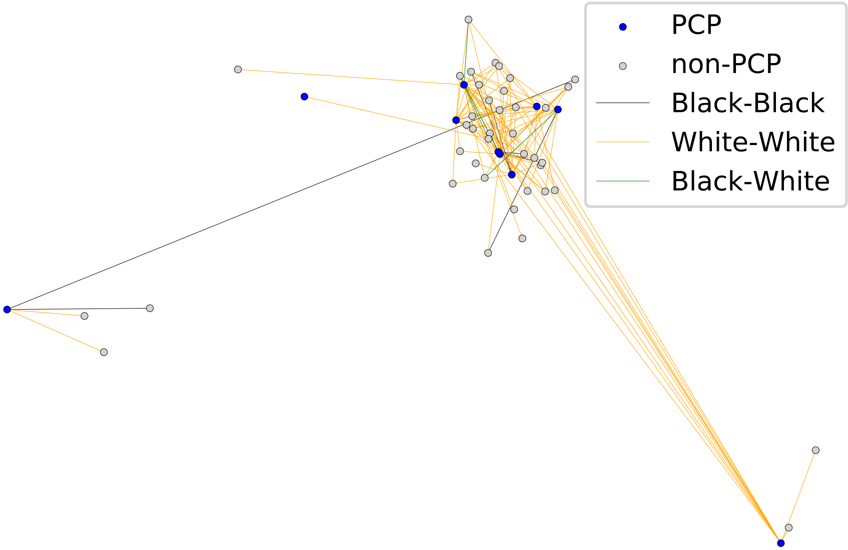

© 2021 Landon BE et al. JAMA Network Open.

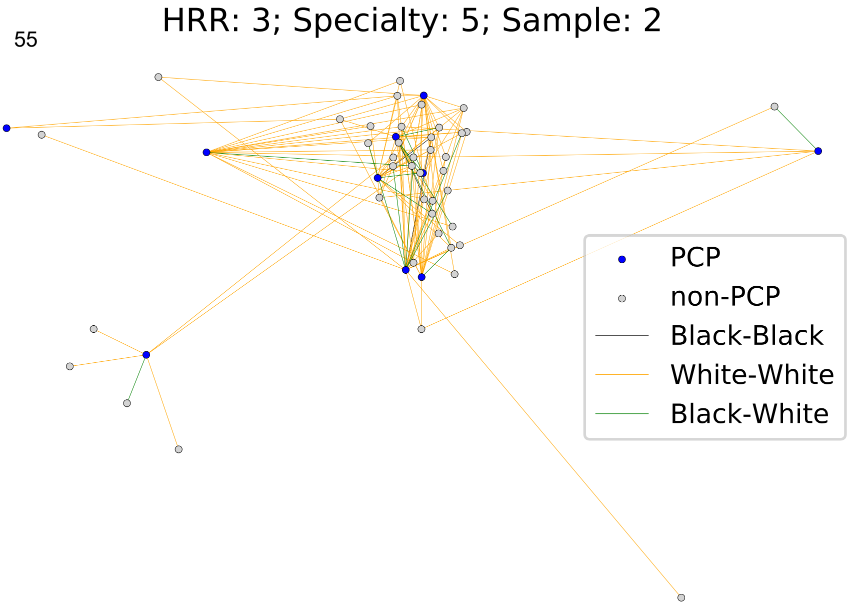

© 2021 Landon BE et al. JAMA Network Open.

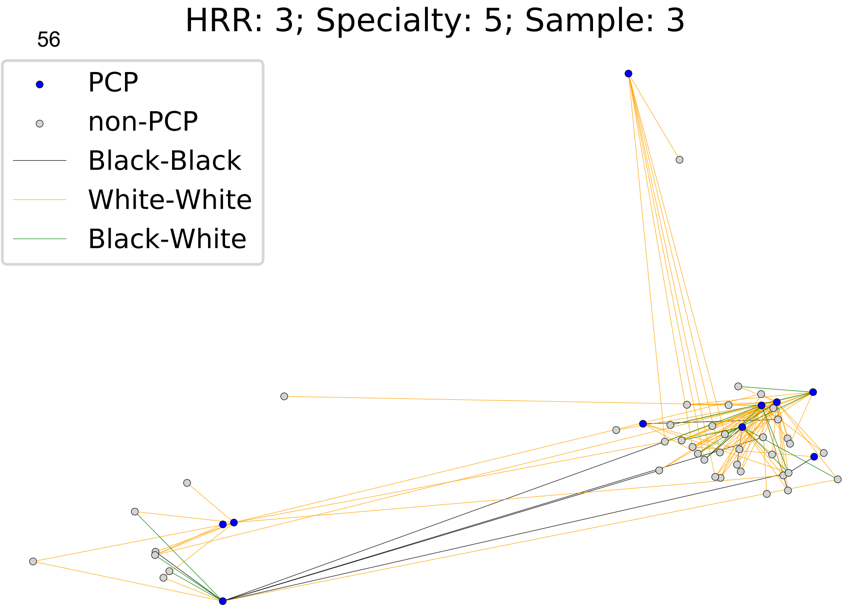

© 2021 Landon BE et al. JAMA Network Open.

HRR: 3; Specialty: 6; Sample: 1

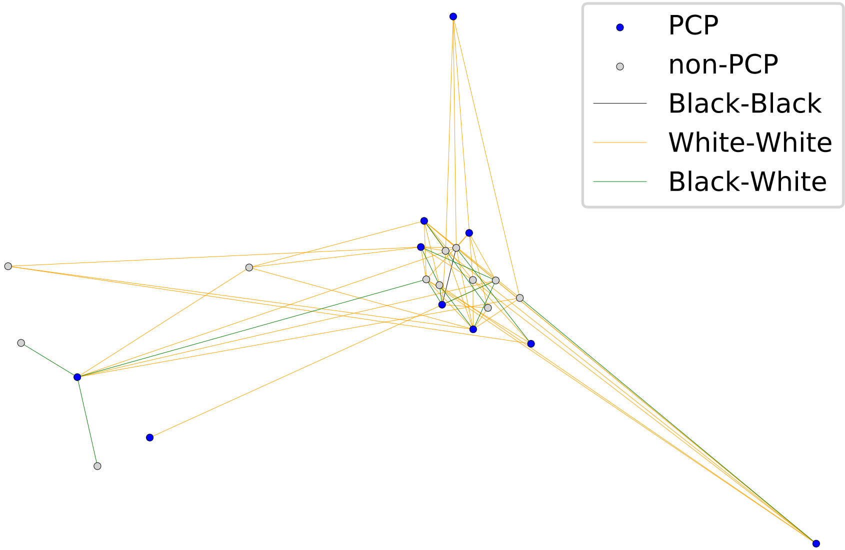

© 2021 Landon BE et al. JAMA Network Open.

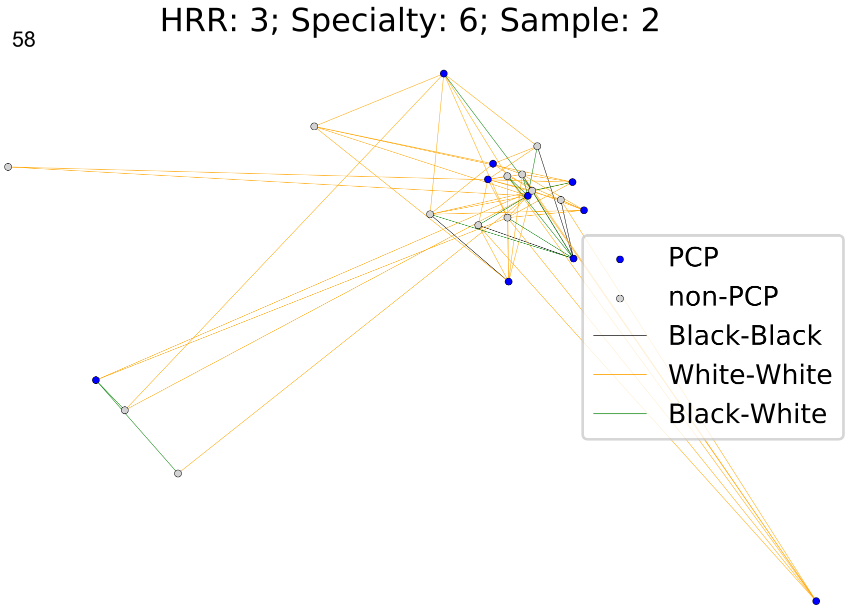

© 2021 Landon BE et al. JAMA Network Open.

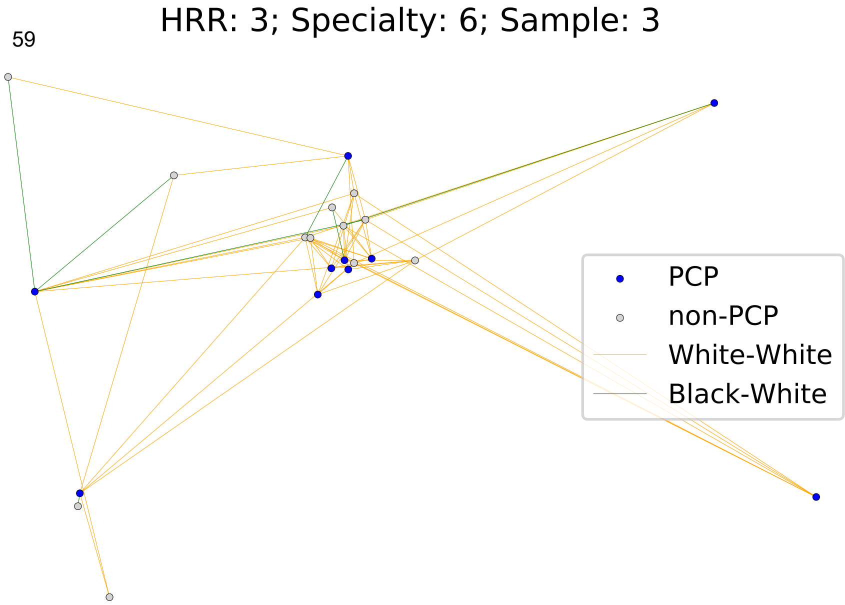

© 2021 Landon BE et al. JAMA Network Open.

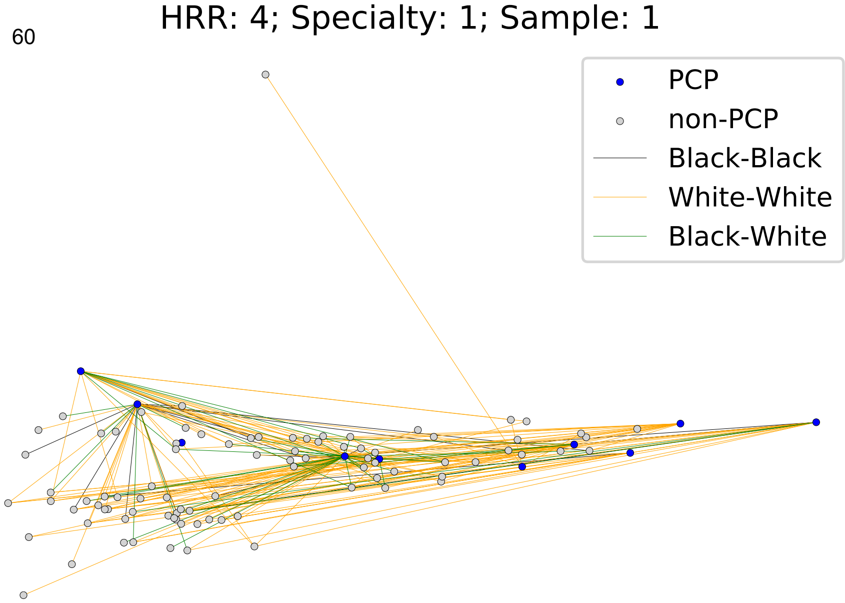

© 2021 Landon BE et al. JAMA Network Open.

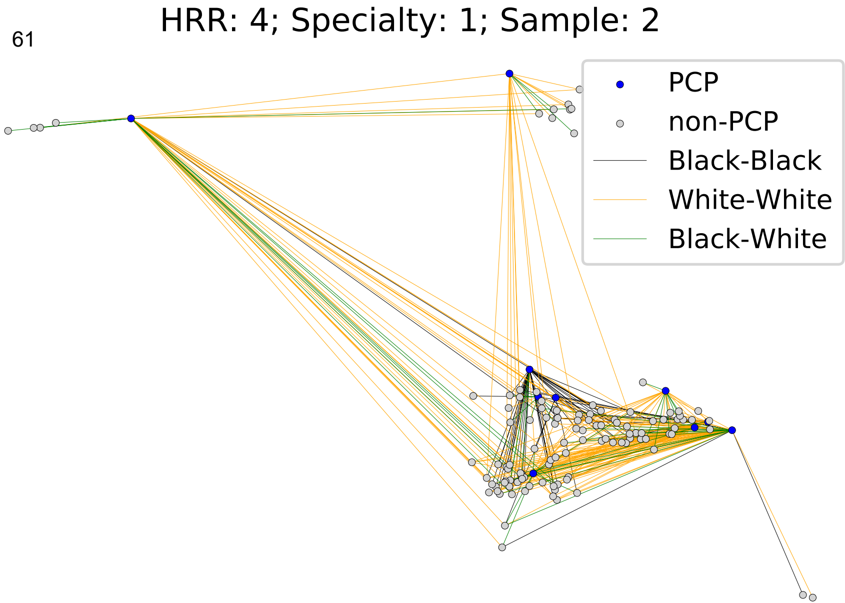

© 2021 Landon BE et al. JAMA Network Open.

HRR: 4; Specialty: 1; Sample: 3

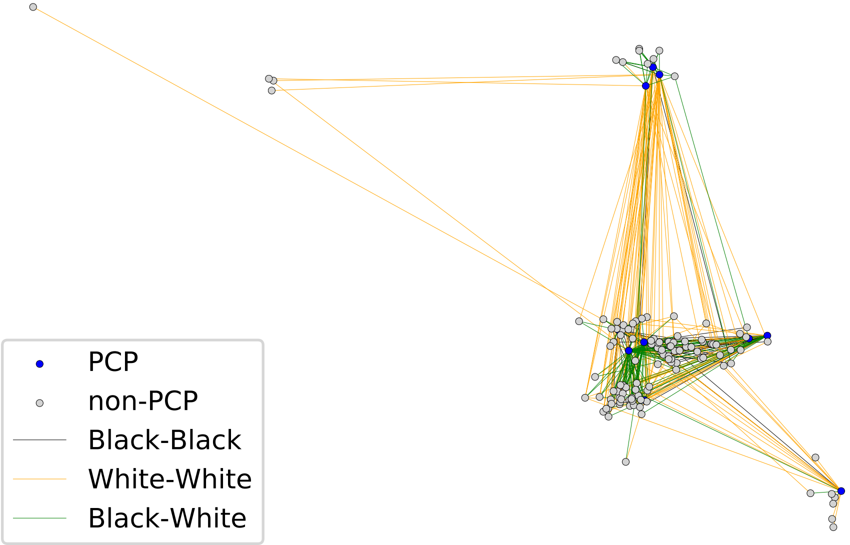

© 2021 Landon BE et al. JAMA Network Open.

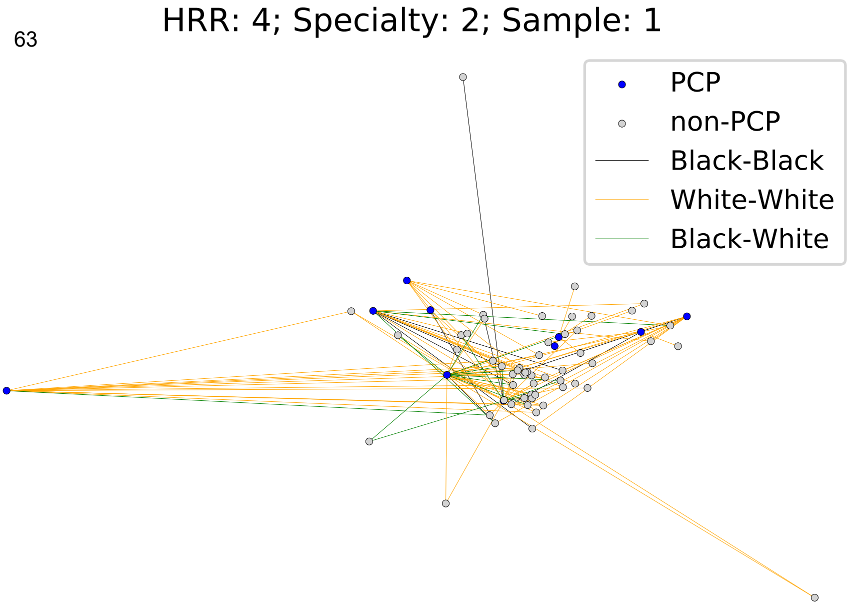

© 2021 Landon BE et al. JAMA Network Open.

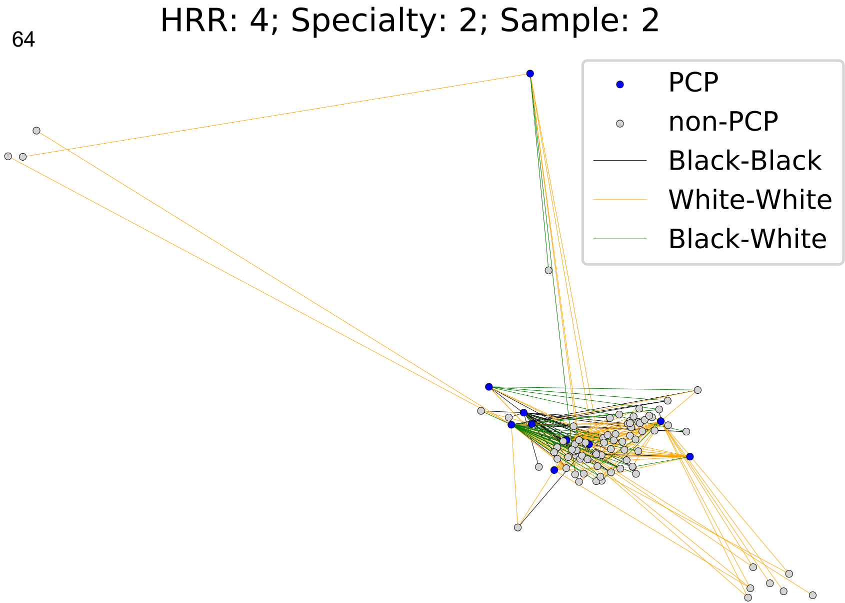

© 2021 Landon BE et al. JAMA Network Open.

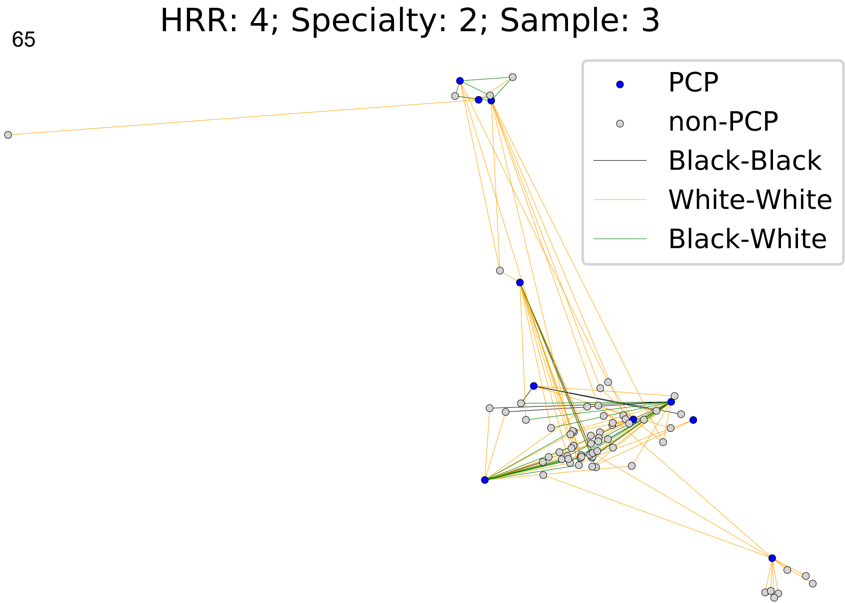

© 2021 Landon BE et al. JAMA Network Open.

HRR: 4; Specialty: 3; Sample: 1

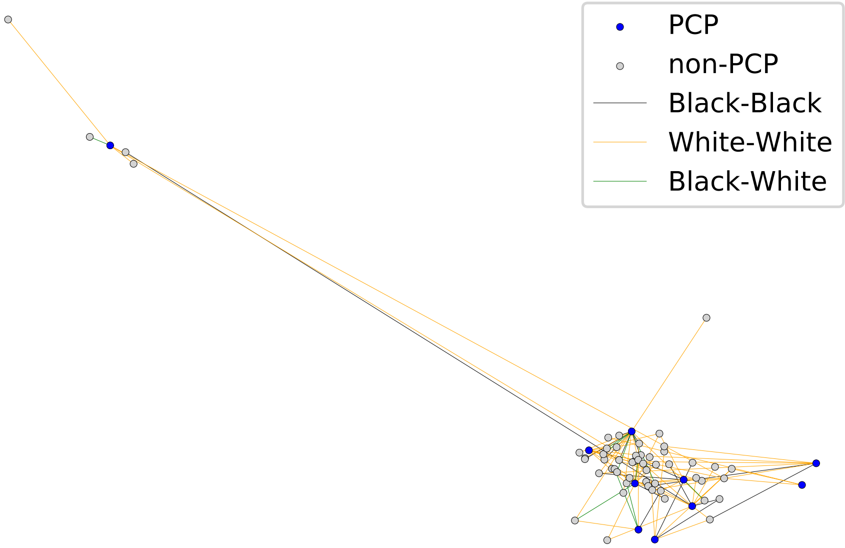

© 2021 Landon BE et al. JAMA Network Open.

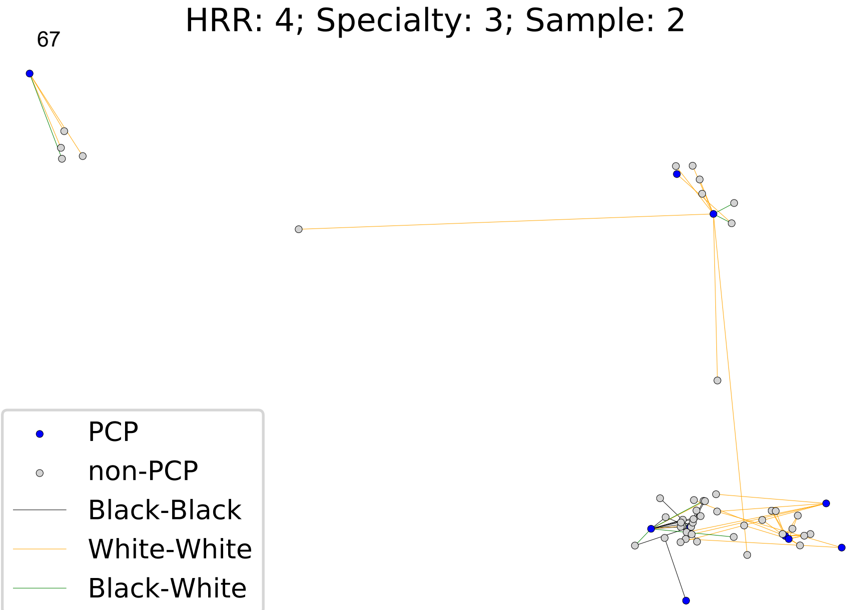

© 2021 Landon BE et al. JAMA Network Open.

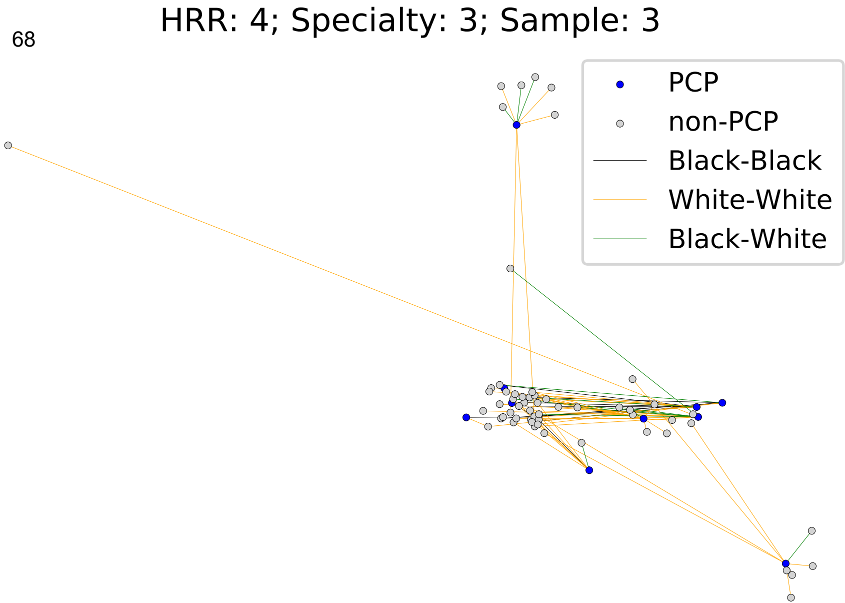

© 2021 Landon BE et al. JAMA Network Open.

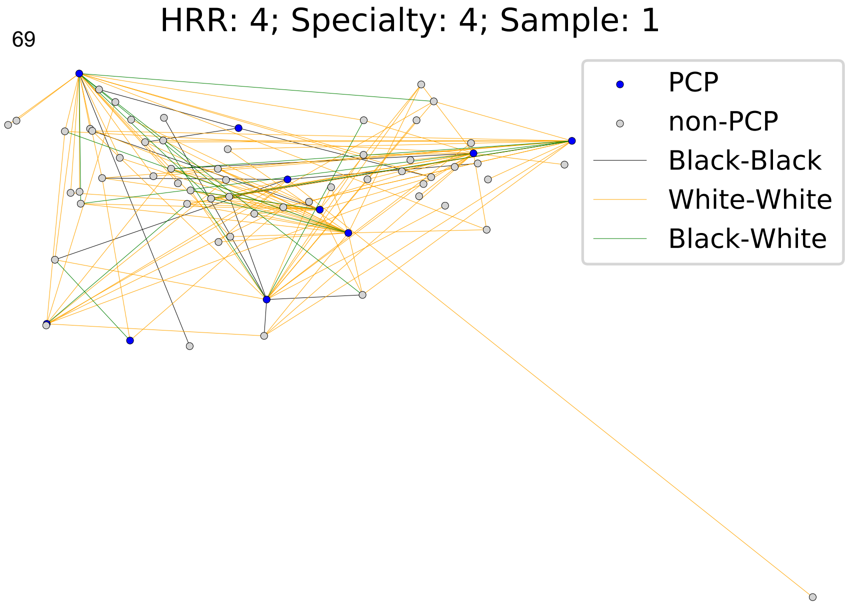

© 2021 Landon BE et al. JAMA Network Open.

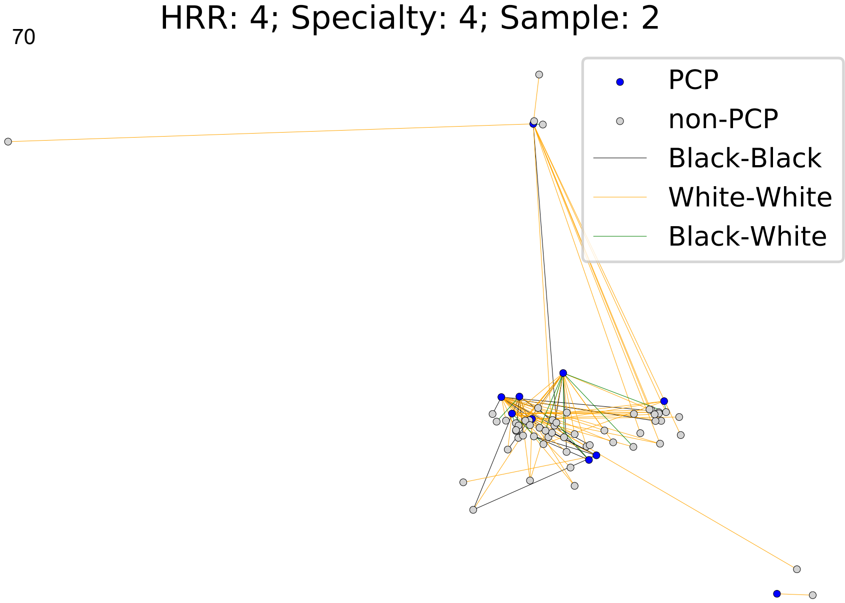

© 2021 Landon BE et al. JAMA Network Open.

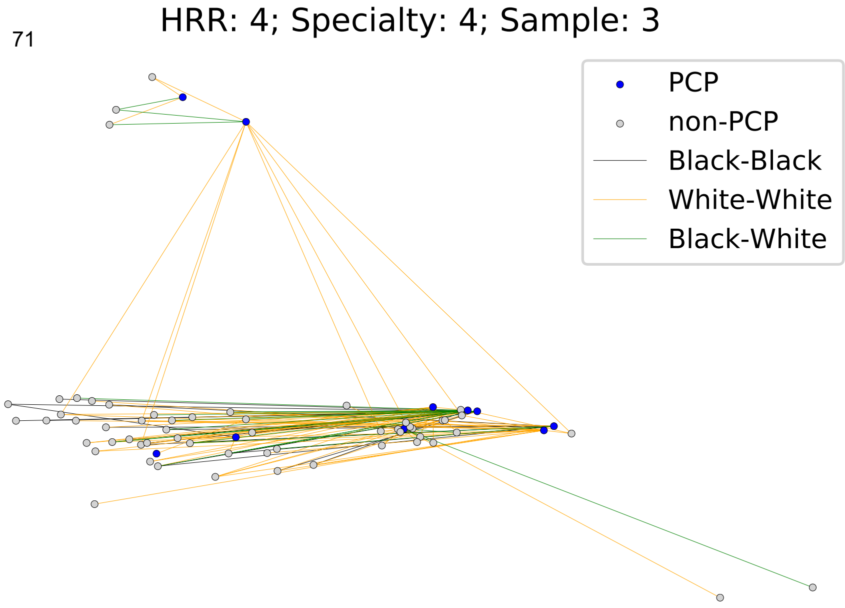

© 2021 Landon BE et al. JAMA Network Open.

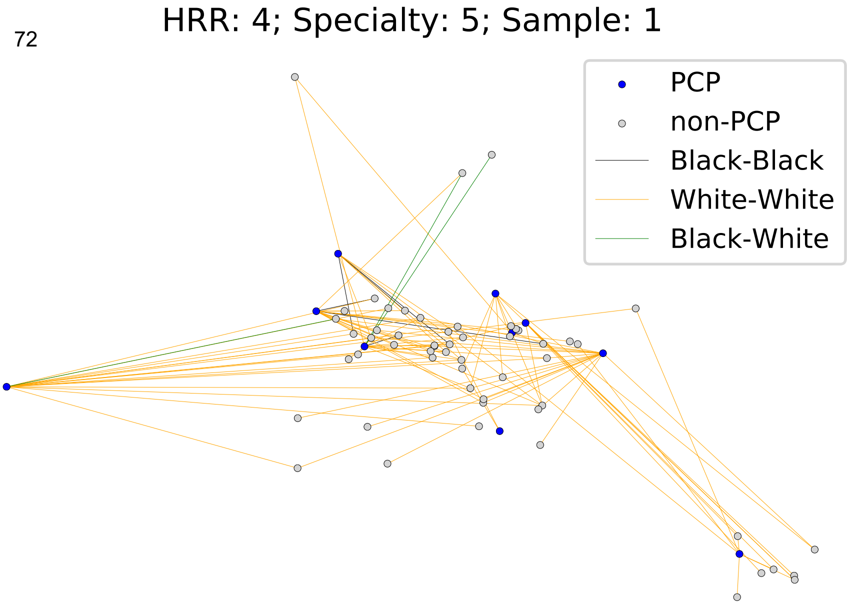

© 2021 Landon BE et al. JAMA Network Open.

HRR: 4; Specialty: 5; Sample: 2

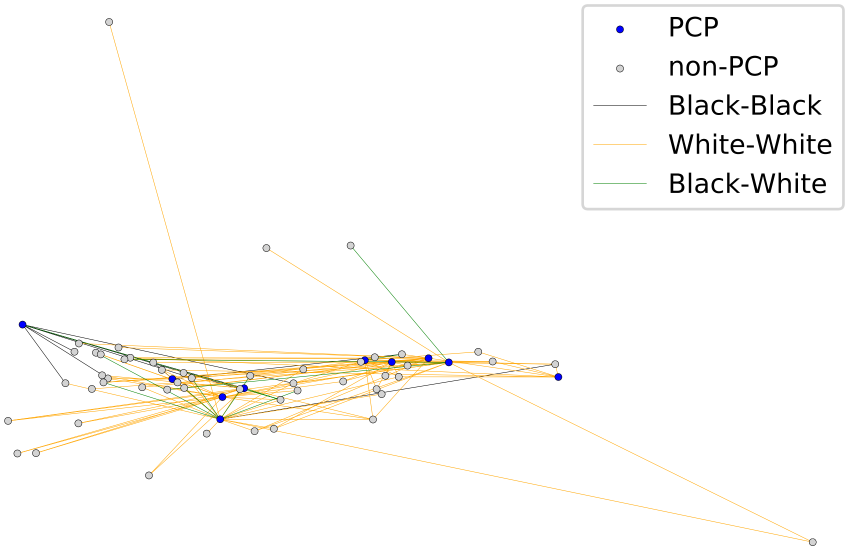

© 2021 Landon BE et al. JAMA Network Open.

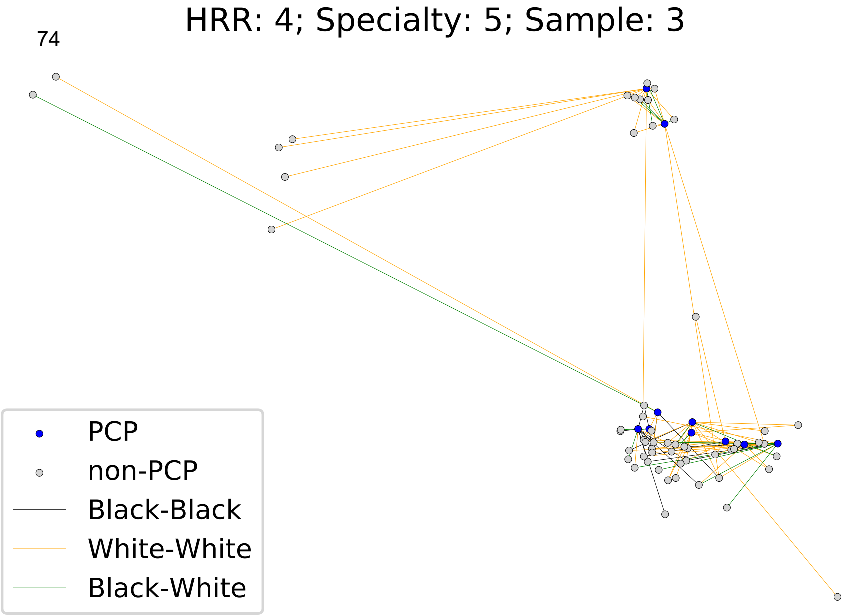

© 2021 Landon BE et al. JAMA Network Open.

HRR: 4; Specialty: 6; Sample: 1

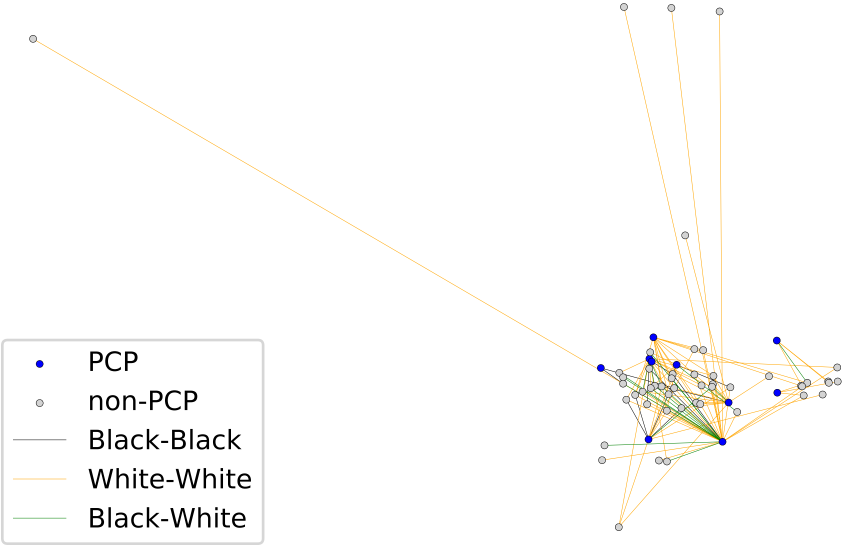

© 2021 Landon BE et al. JAMA Network Open.

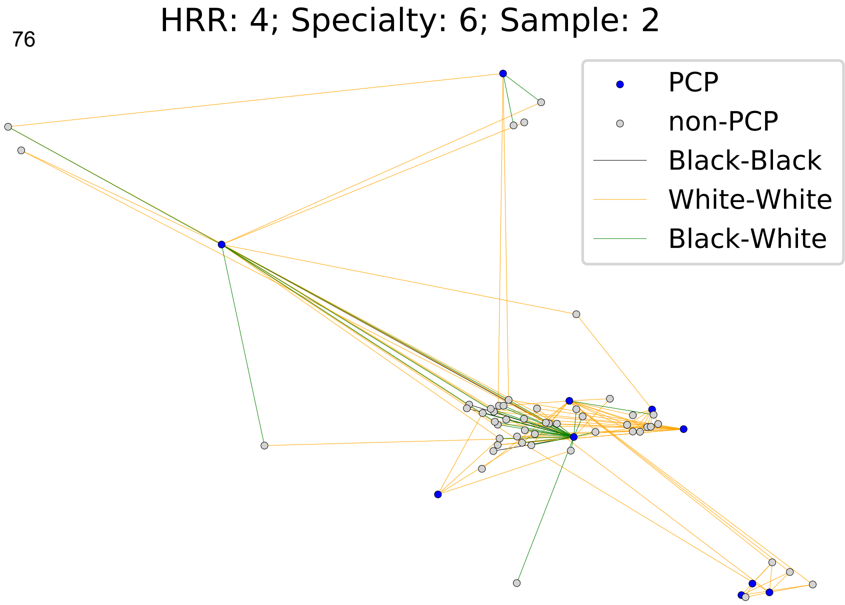

© 2021 Landon BE et al. JAMA Network Open.

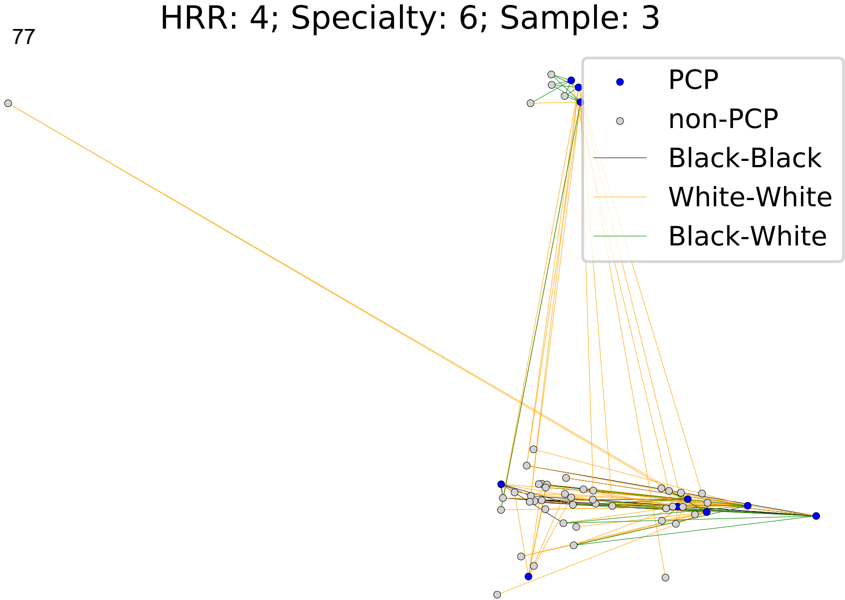

© 2021 Landon BE et al. JAMA Network Open.

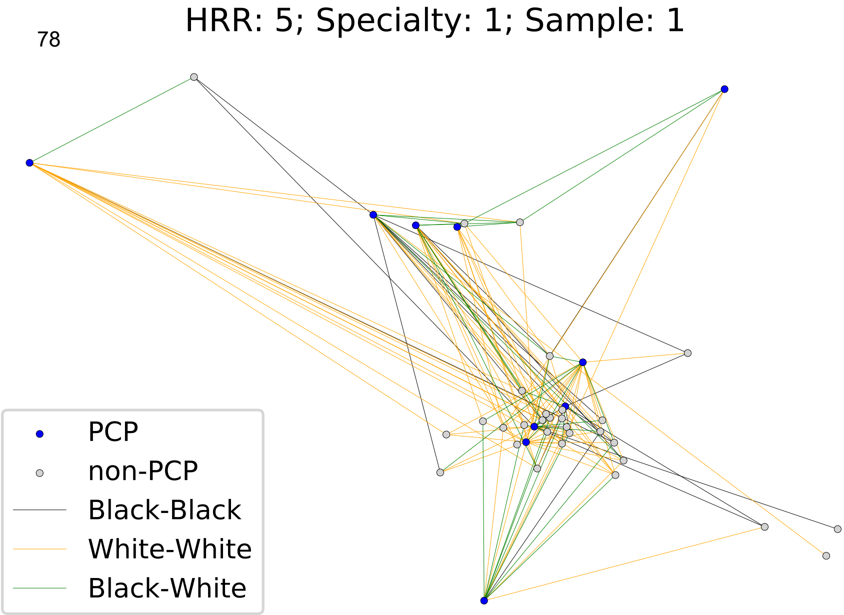

© 2021 Landon BE et al. JAMA Network Open.

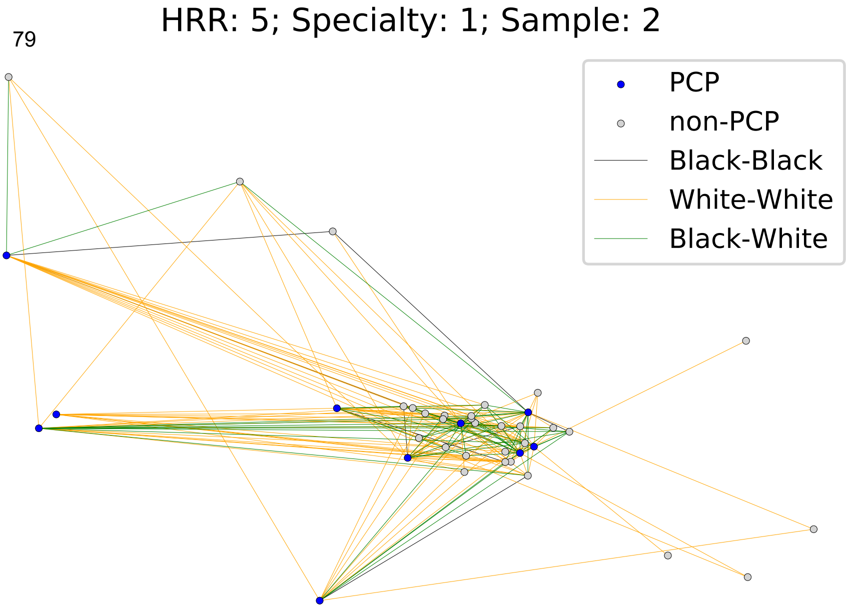

© 2021 Landon BE et al. JAMA Network Open.

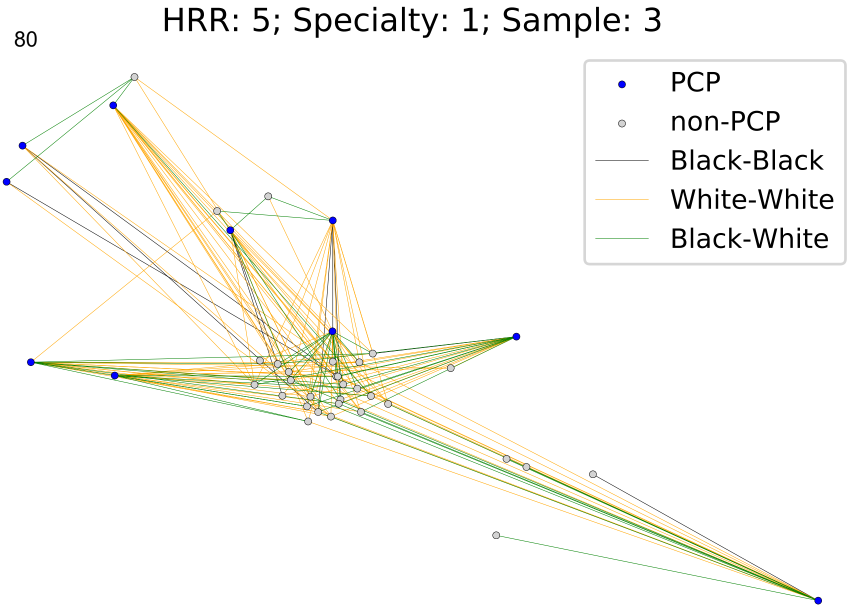

© 2021 Landon BE et al. JAMA Network Open.

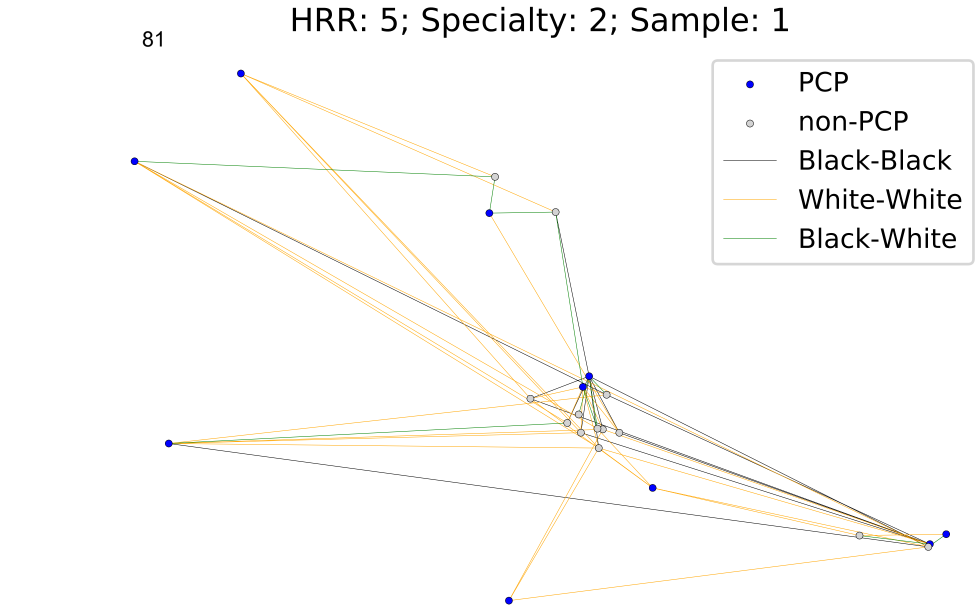

© 2021 Landon BE et al. JAMA Network Open.

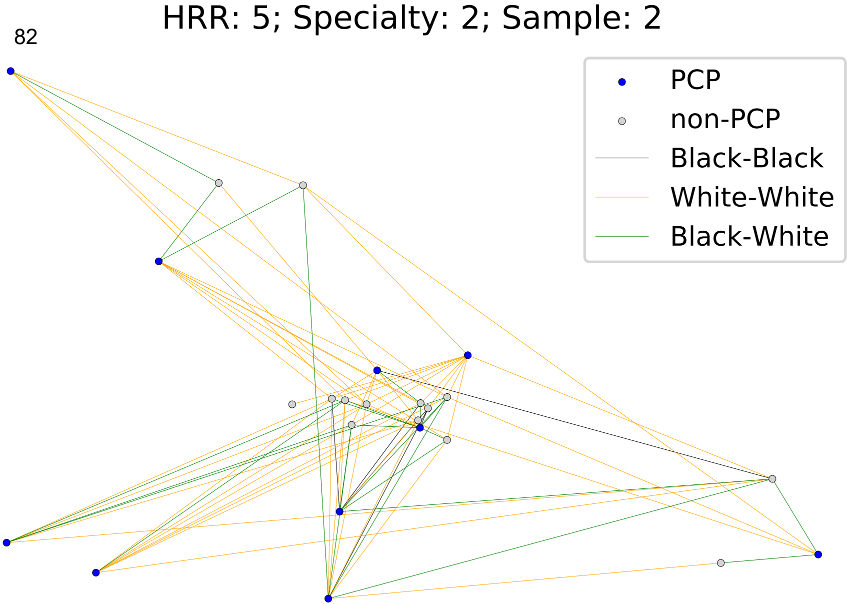

© 2021 Landon BE et al. JAMA Network Open.

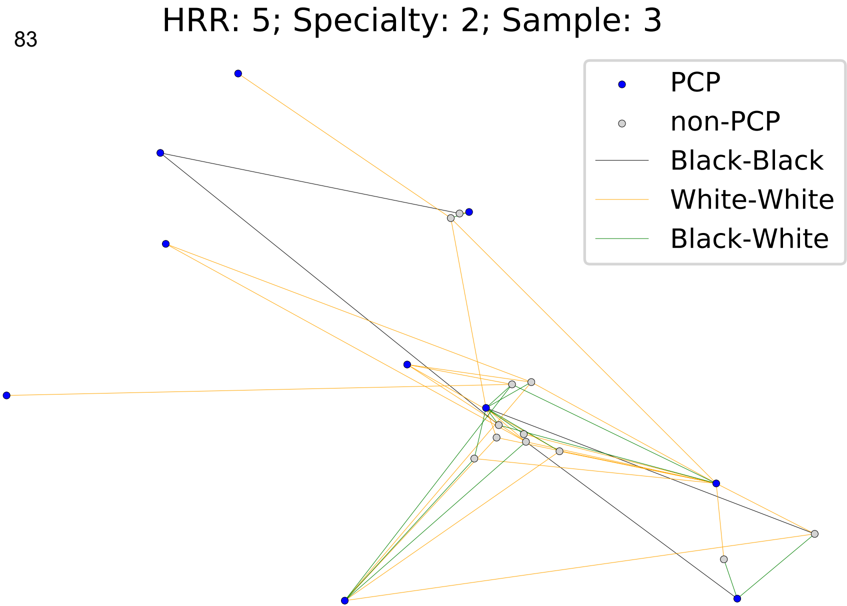

© 2021 Landon BE et al. JAMA Network Open.

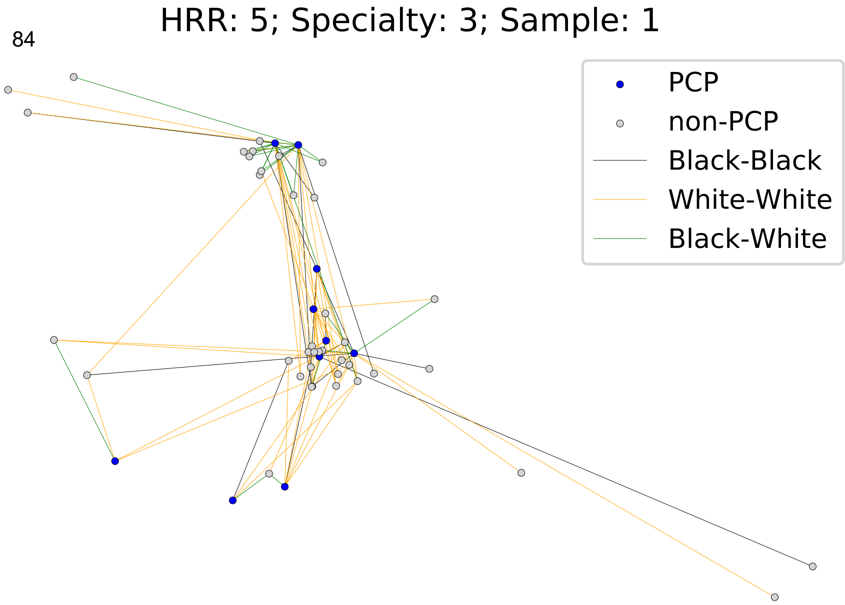

© 2021 Landon BE et al. JAMA Network Open.

HRR: 5; Specialty: 3; Sample: 2

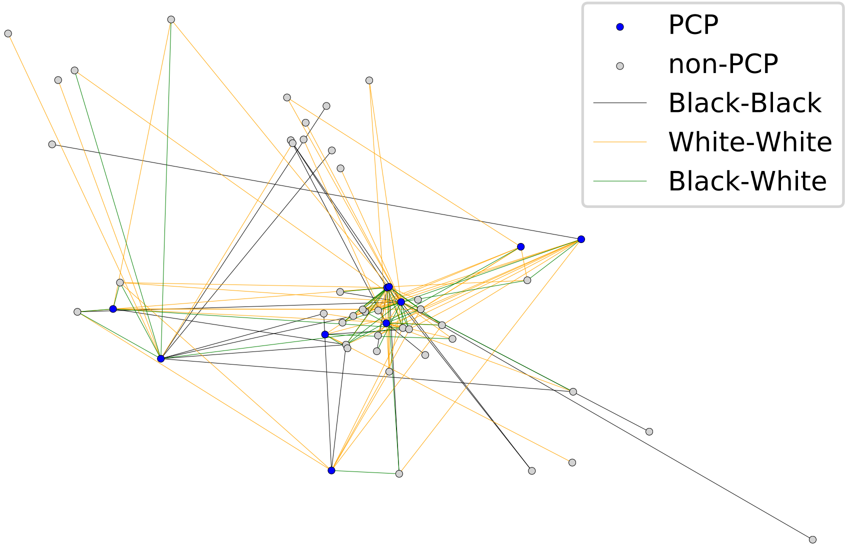

© 2021 Landon BE et al. JAMA Network Open.

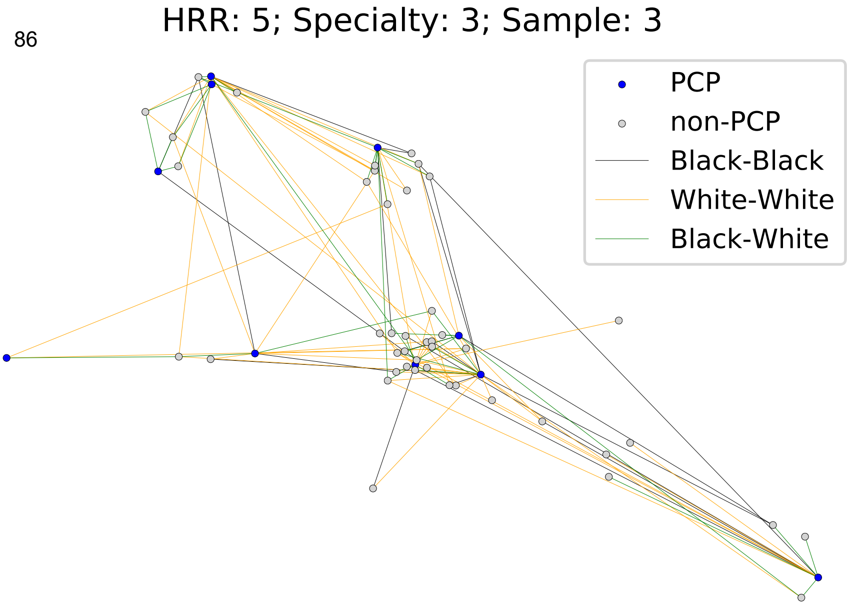

© 2021 Landon BE et al. JAMA Network Open.

HRR: 5; Specialty: 4; Sample: 1

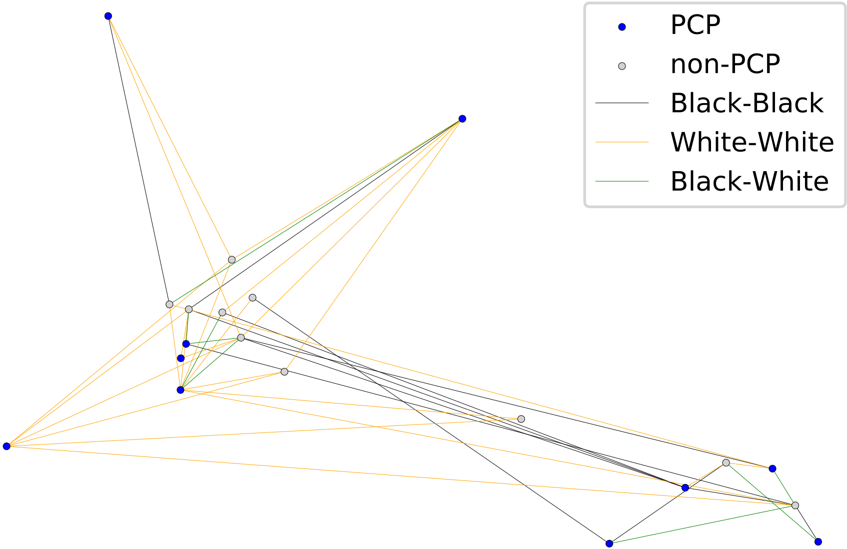

© 2021 Landon BE et al. JAMA Network Open.

HRR: 5; Specialty: 4; Sample: 2

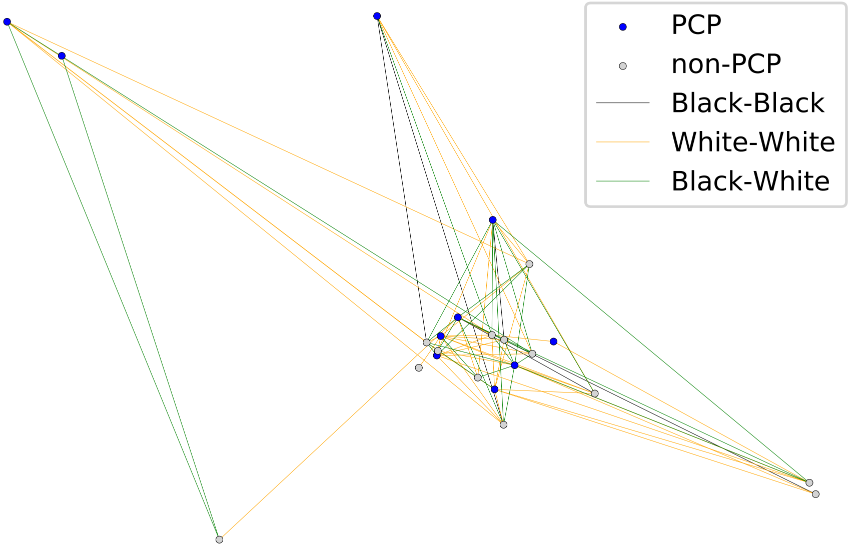

© 2021 Landon BE et al. JAMA Network Open.

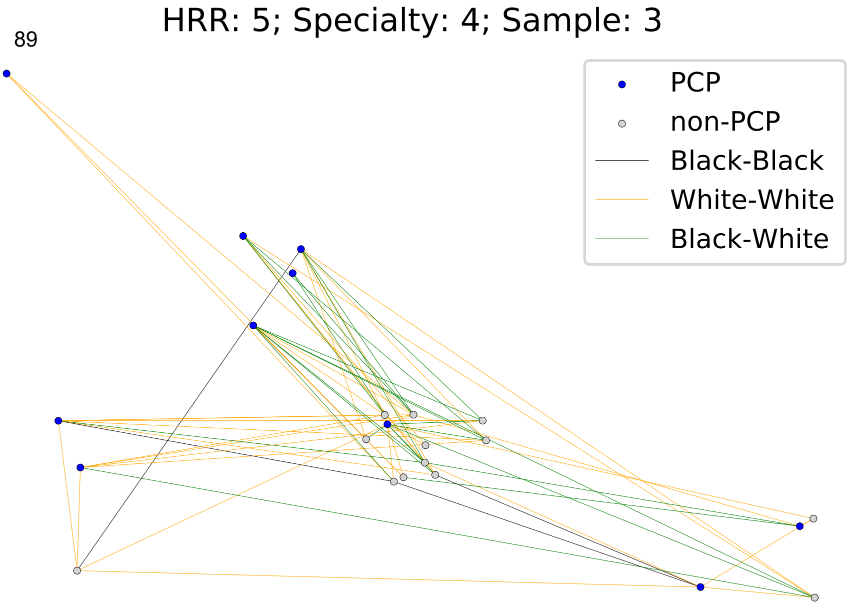

© 2021 Landon BE et al. JAMA Network Open.

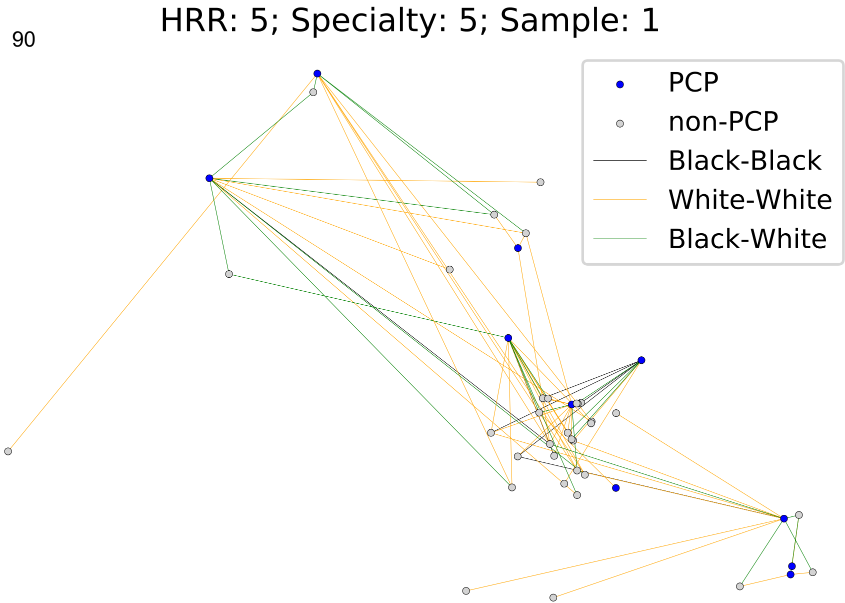

© 2021 Landon BE et al. JAMA Network Open.

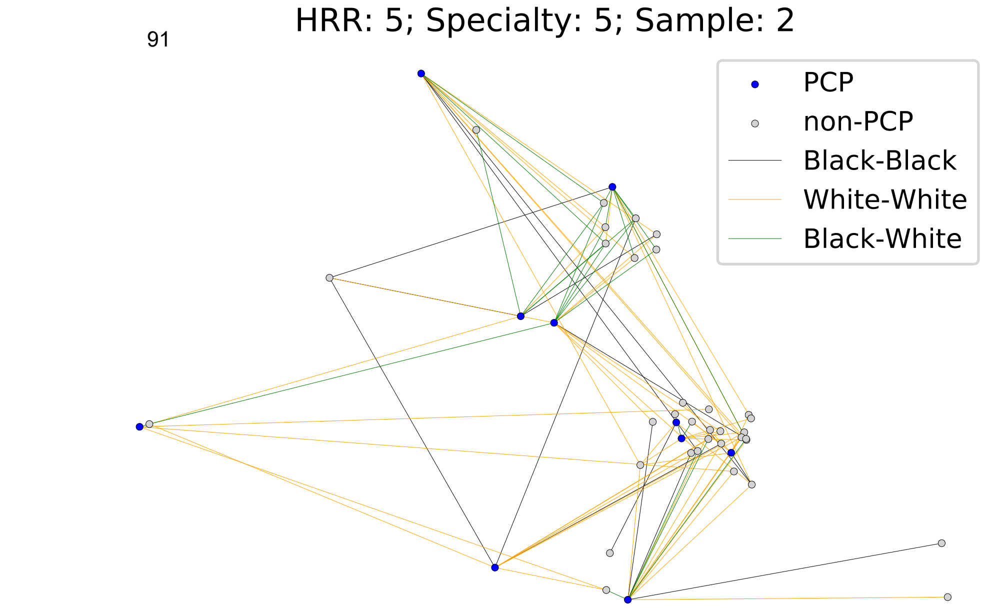

© 2021 Landon BE et al. JAMA Network Open.

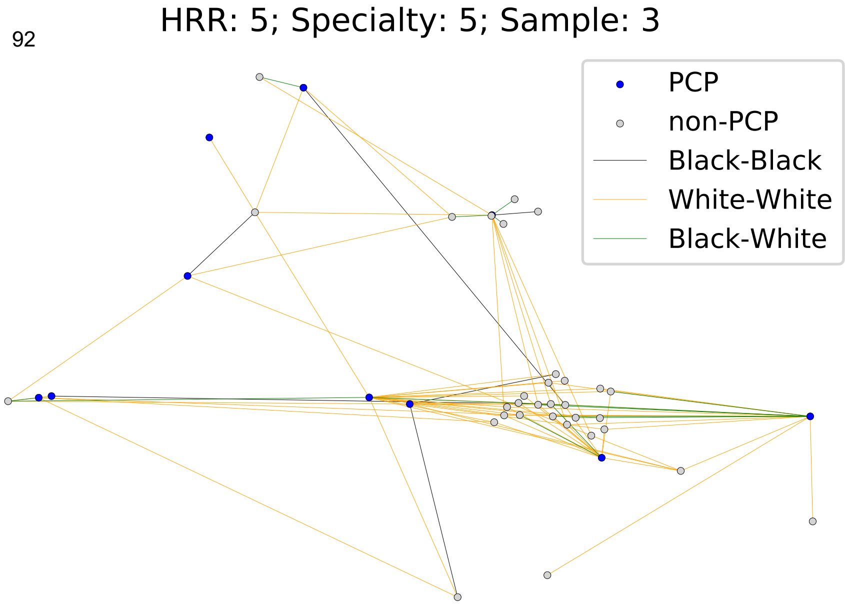

© 2021 Landon BE et al. JAMA Network Open.

HRR: 5; Specialty: 6; Sample: 1

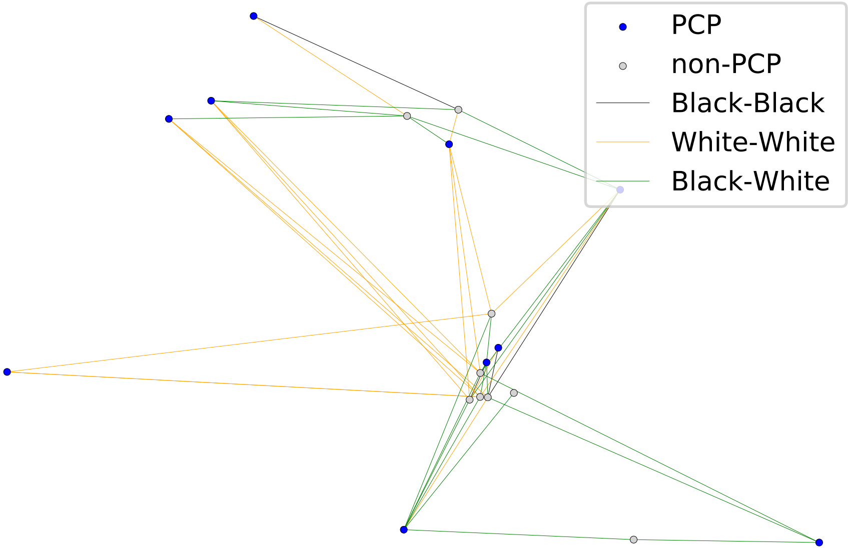

© 2021 Landon BE et al. JAMA Network Open.

HRR: 5; Specialty: 6; Sample: 2

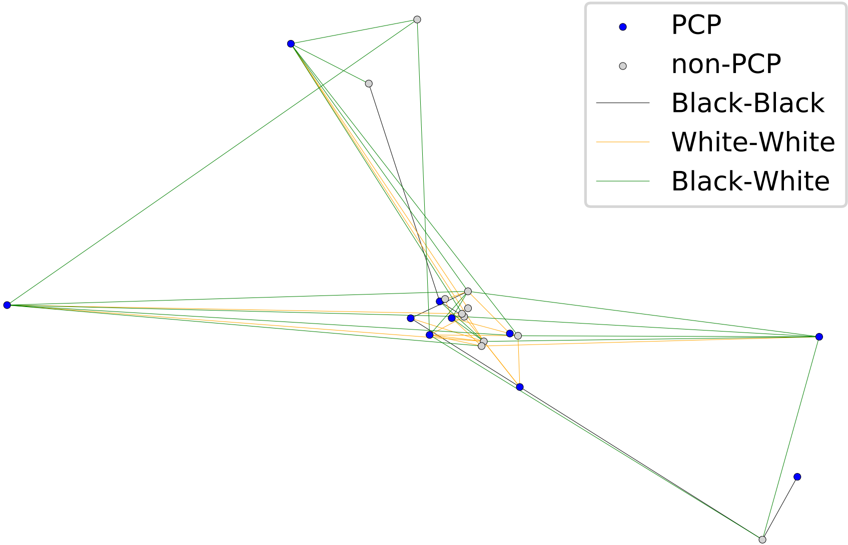

© 2021 Landon BE et al. JAMA Network Open.

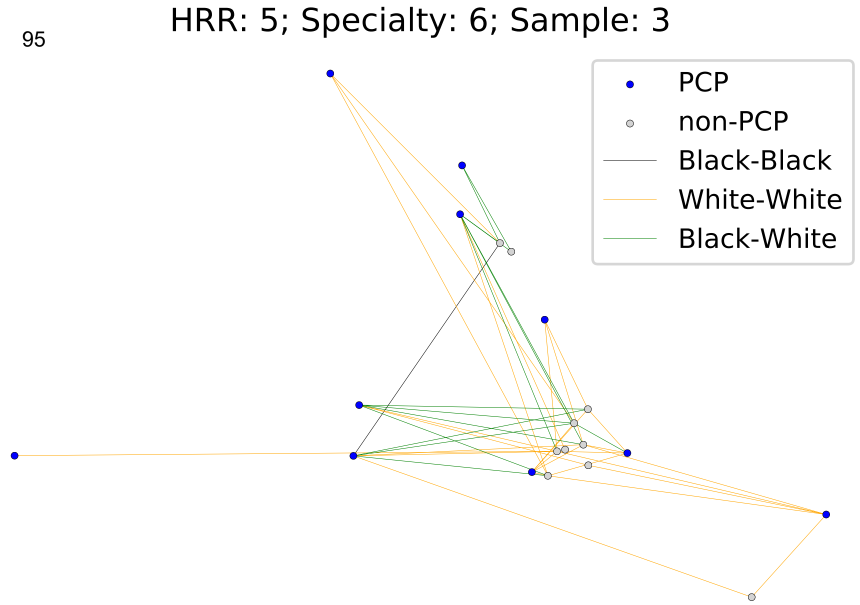

© 2021 Landon BE et al. JAMA Network Open.

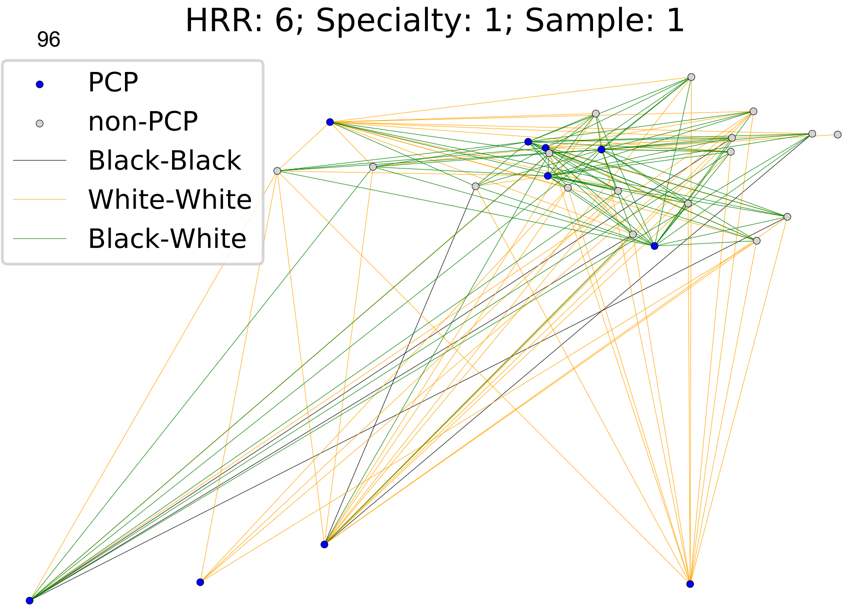

© 2021 Landon BE et al. JAMA Network Open.

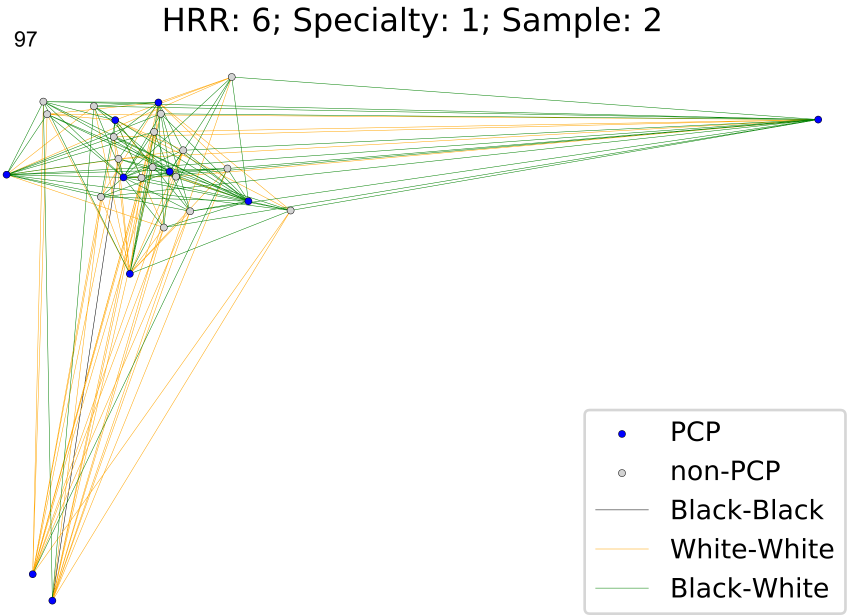

© 2021 Landon BE et al. JAMA Network Open.

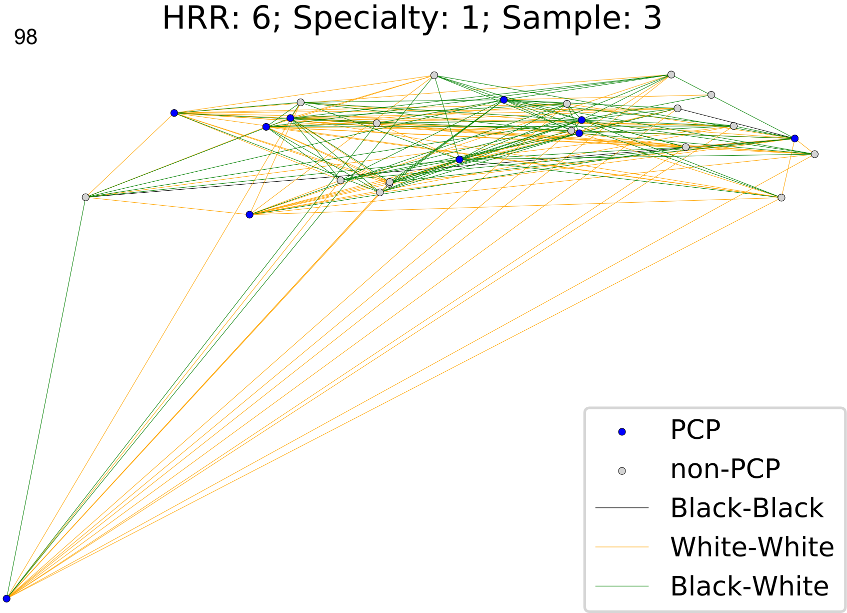

© 2021 Landon BE et al. JAMA Network Open.

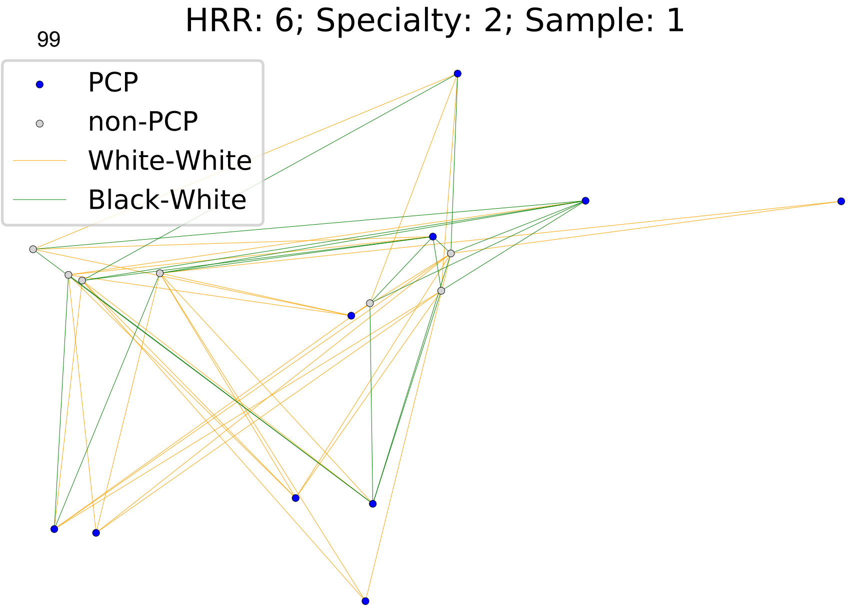

© 2021 Landon BE et al. JAMA Network Open.

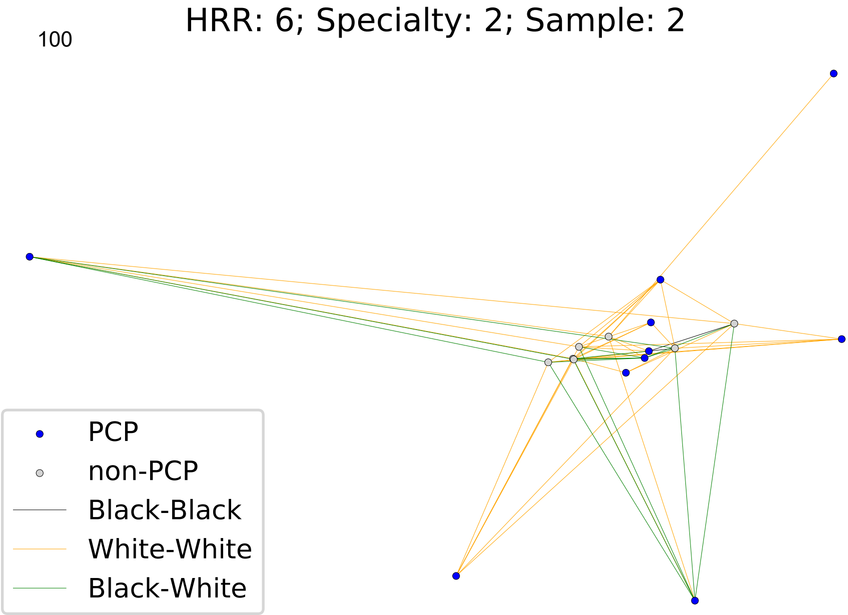

© 2021 Landon BE et al. JAMA Network Open.

HRR: 6; Specialty: 2; Sample: 3

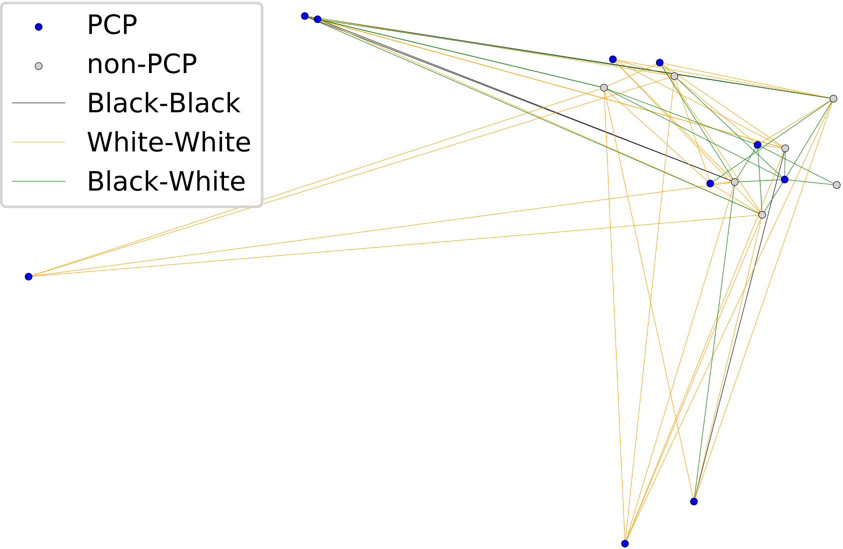

© 2021 Landon BE et al. JAMA Network Open.

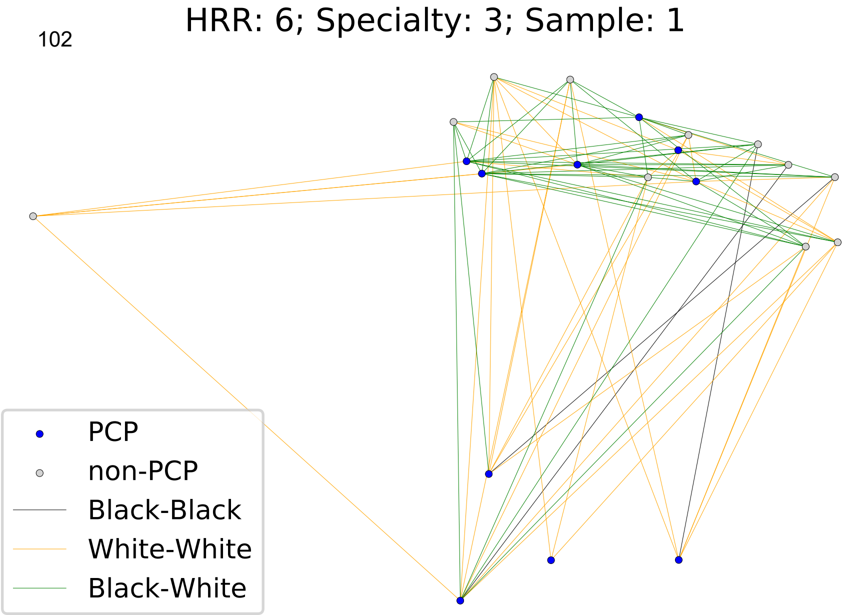

© 2021 Landon BE et al. JAMA Network Open.

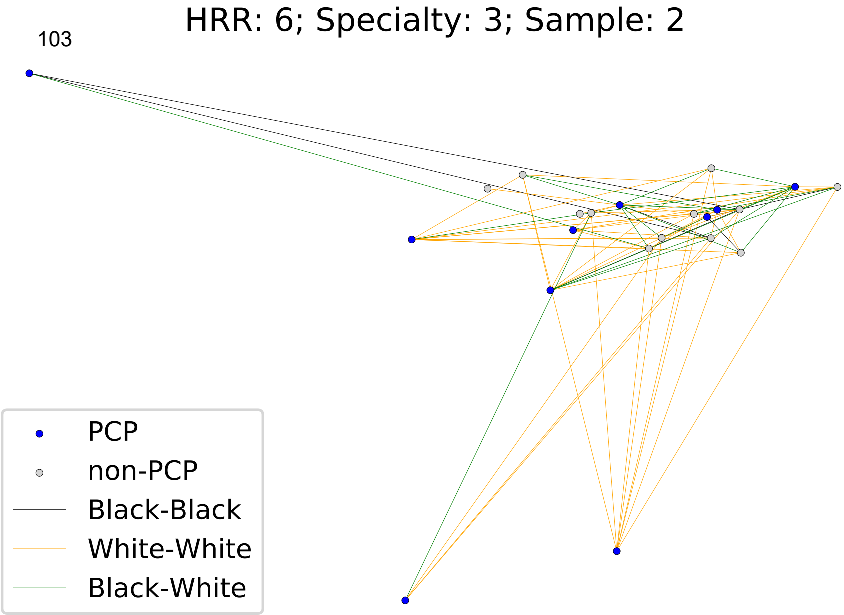

© 2021 Landon BE et al. JAMA Network Open.

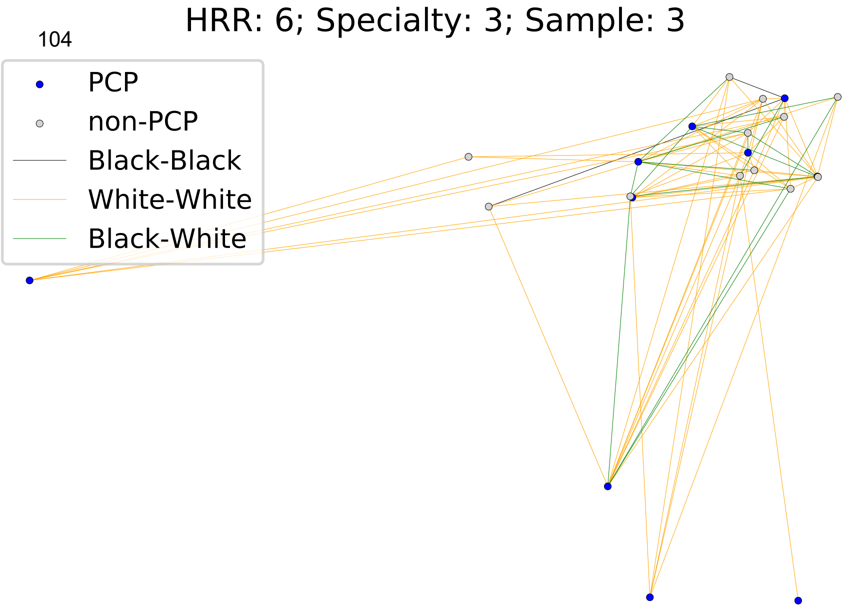

© 2021 Landon BE et al. JAMA Network Open.

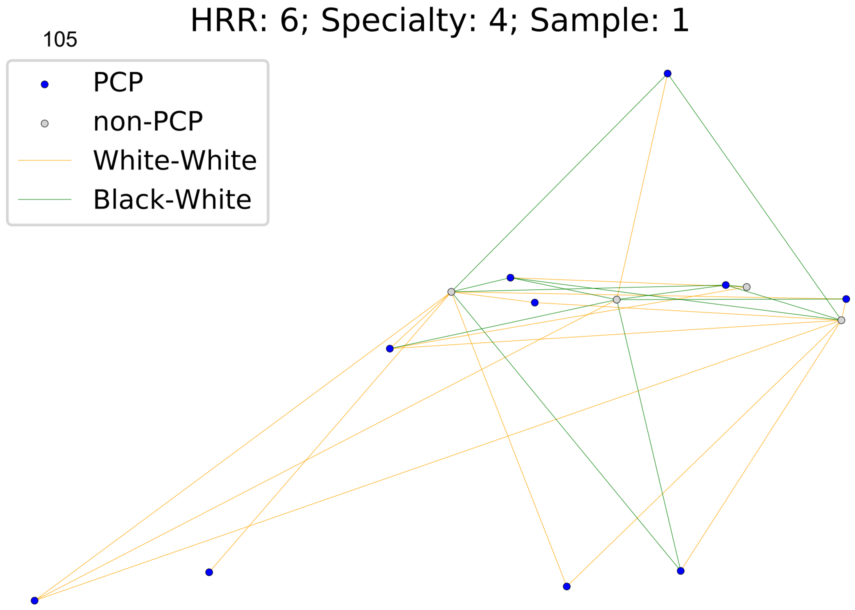

© 2021 Landon BE et al. JAMA Network Open.

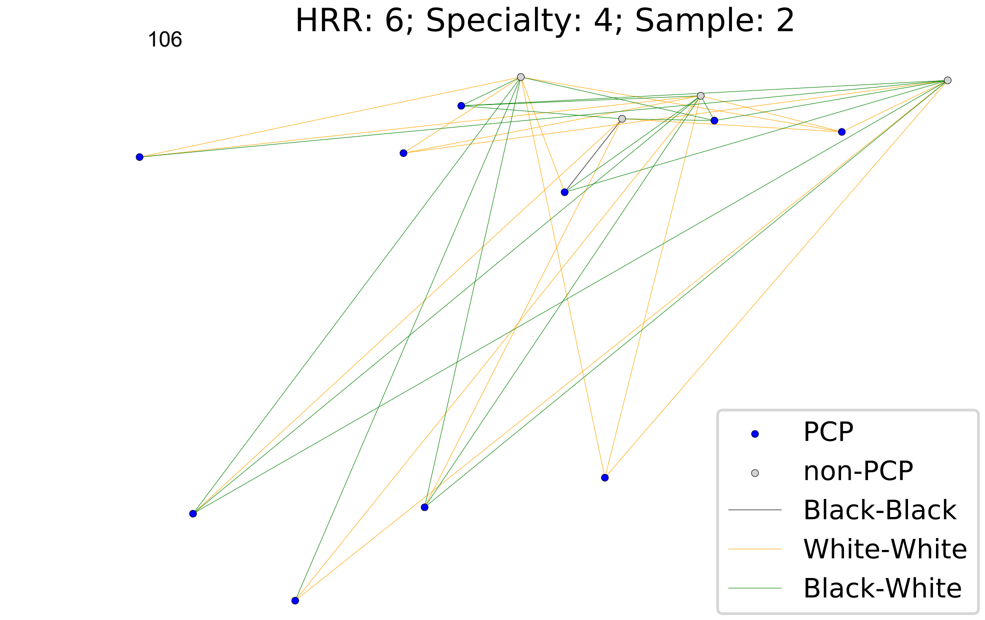

© 2021 Landon BE et al. JAMA Network Open.

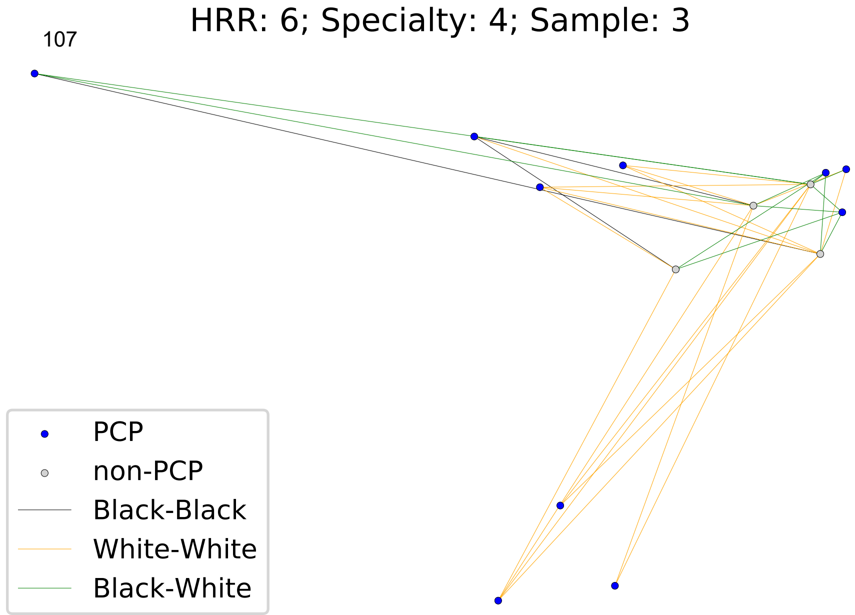

© 2021 Landon BE et al. JAMA Network Open.

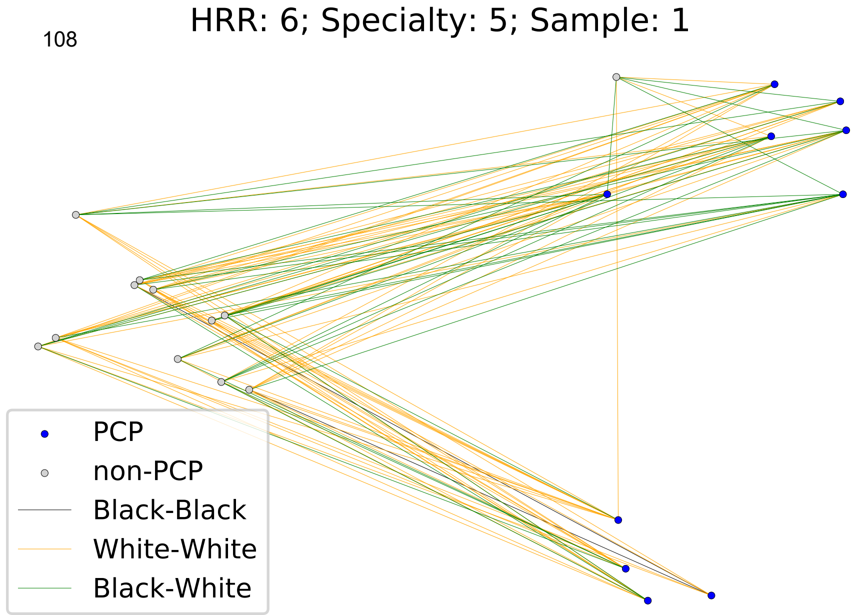

© 2021 Landon BE et al. JAMA Network Open.

HRR: 6; Specialty: 5; Sample: 2

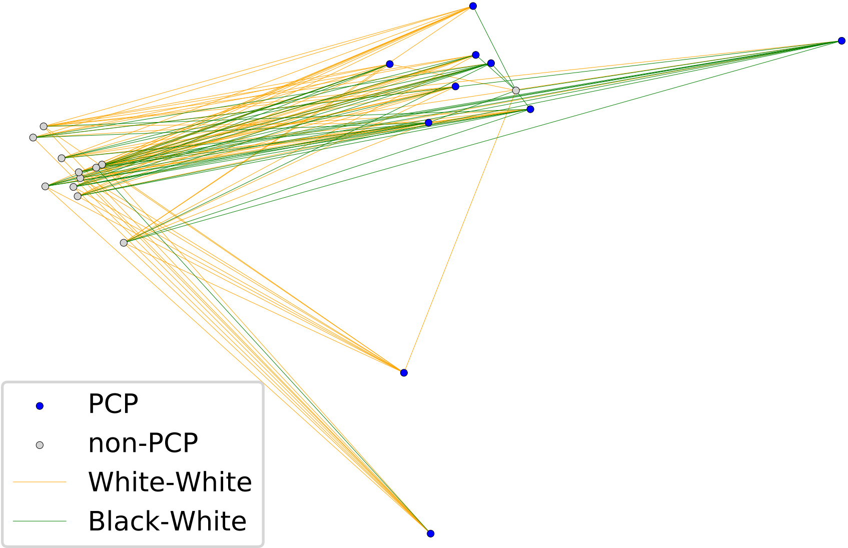

© 2021 Landon BE et al. JAMA Network Open.

HRR: 6; Specialty: 5; Sample: 3

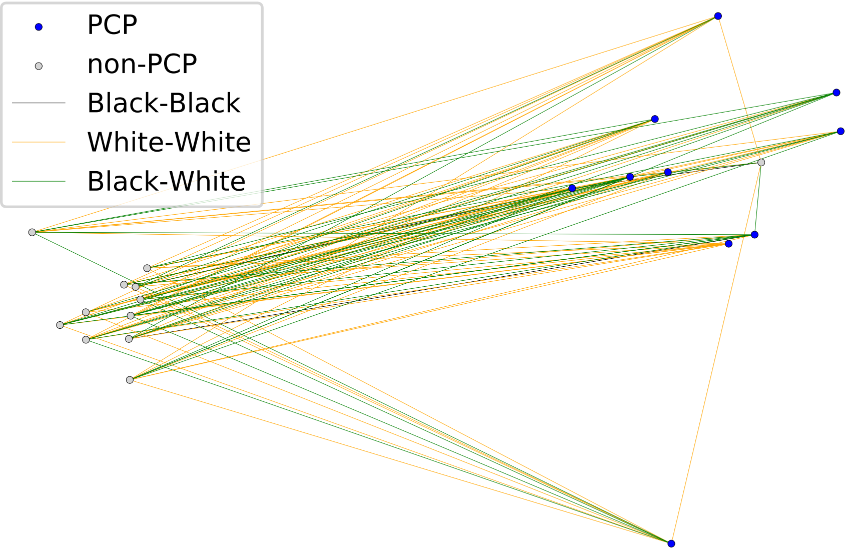

© 2021 Landon BE et al. JAMA Network Open.

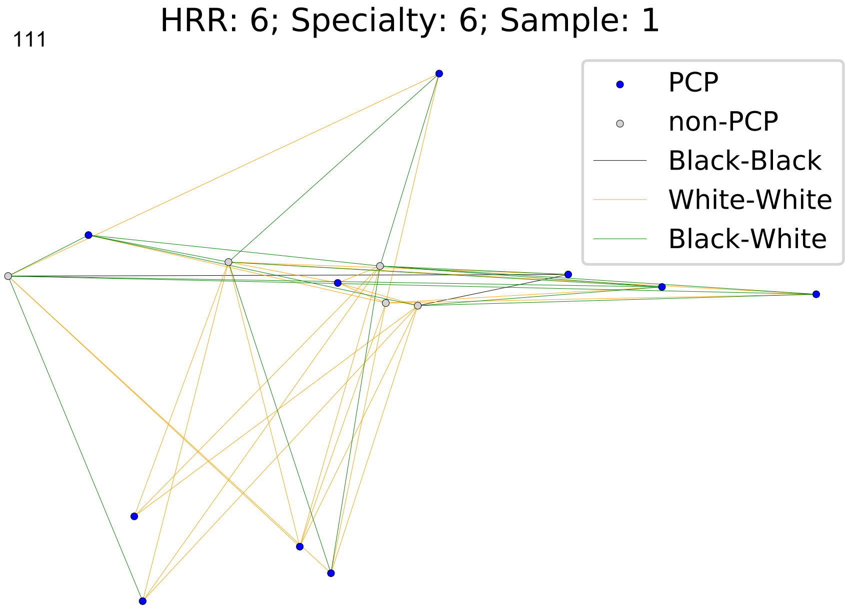

© 2021 Landon BE et al. JAMA Network Open.

HRR: 6; Specialty: 6; Sample: 2

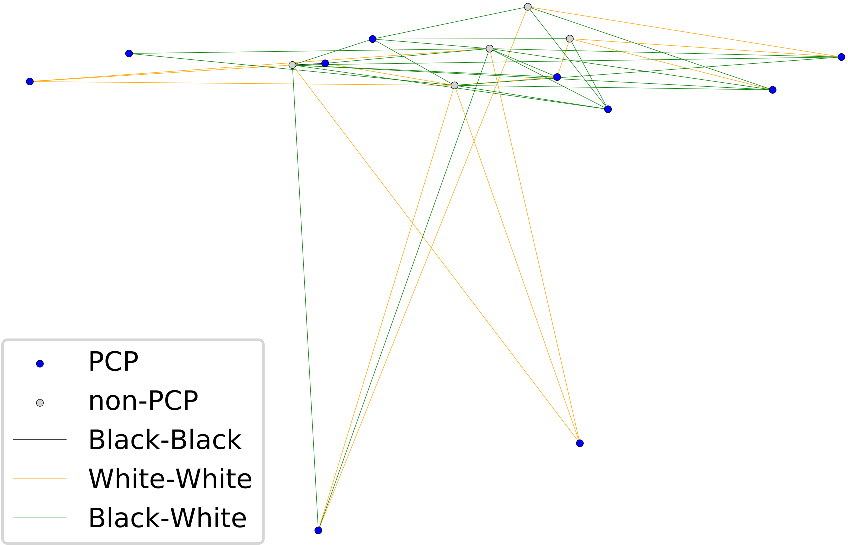

© 2021 Landon BE et al. JAMA Network Open.

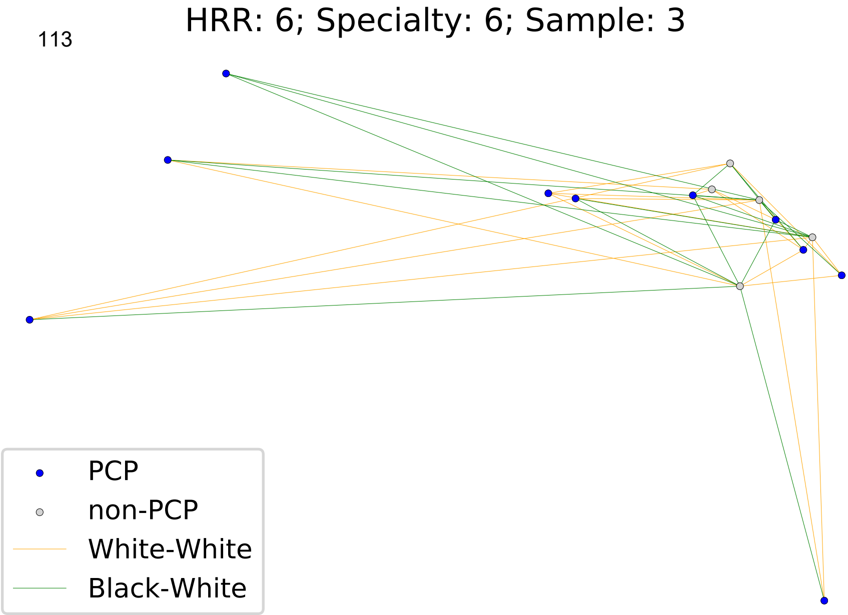

© 2021 Landon BE et al. JAMA Network Open.

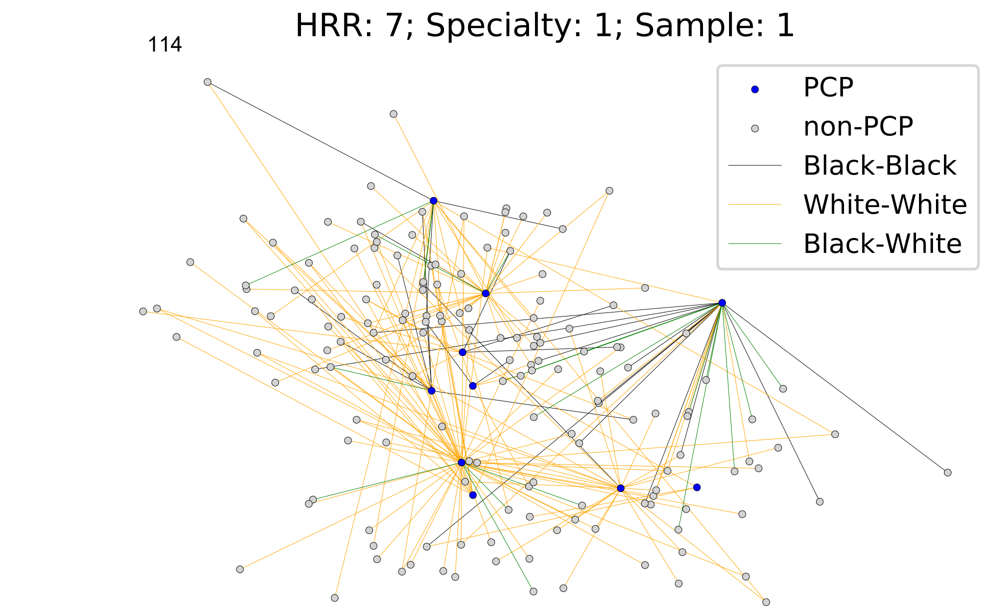

© 2021 Landon BE et al. JAMA Network Open.

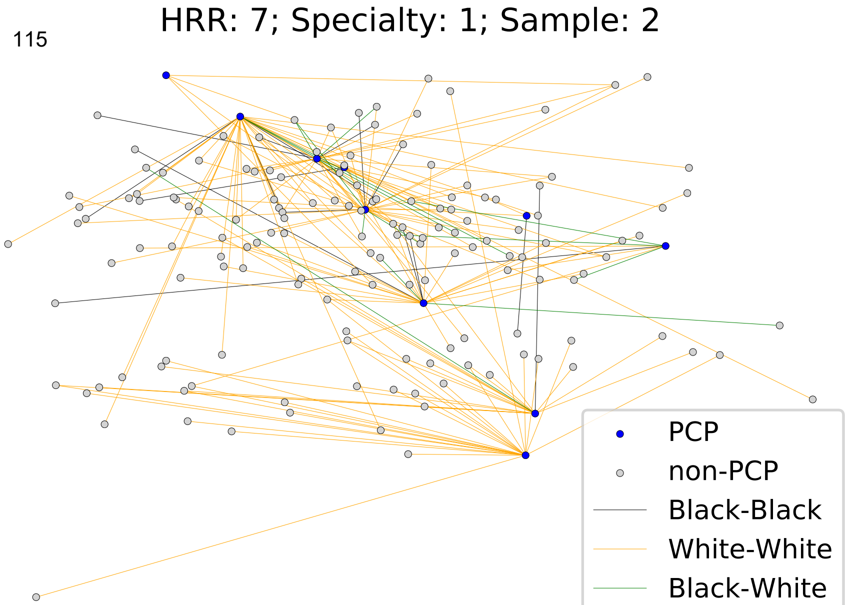

© 2021 Landon BE et al. JAMA Network Open.

HRR: 7; Specialty: 1; Sample: 3

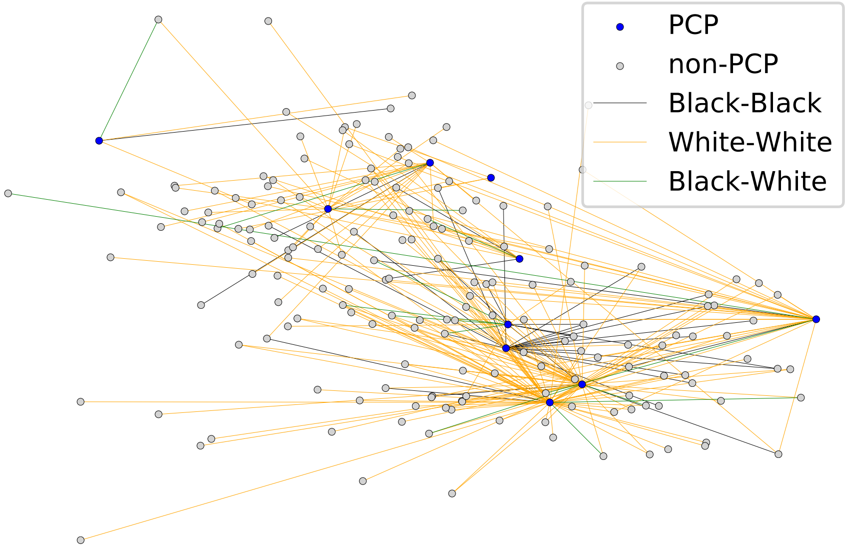

© 2021 Landon BE et al. JAMA Network Open.

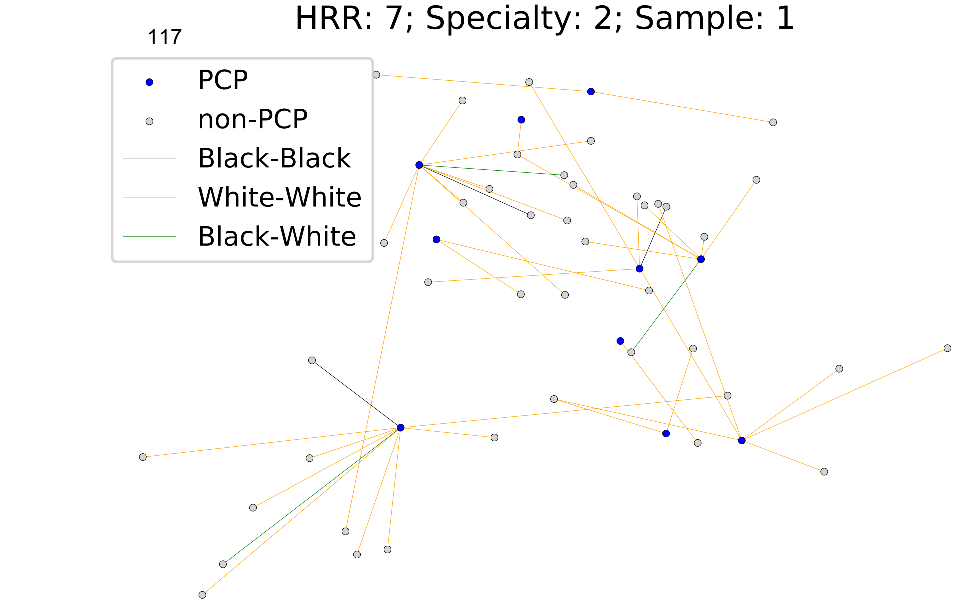

© 2021 Landon BE et al. JAMA Network Open.

HRR: 7; Specialty: 2; Sample: 2

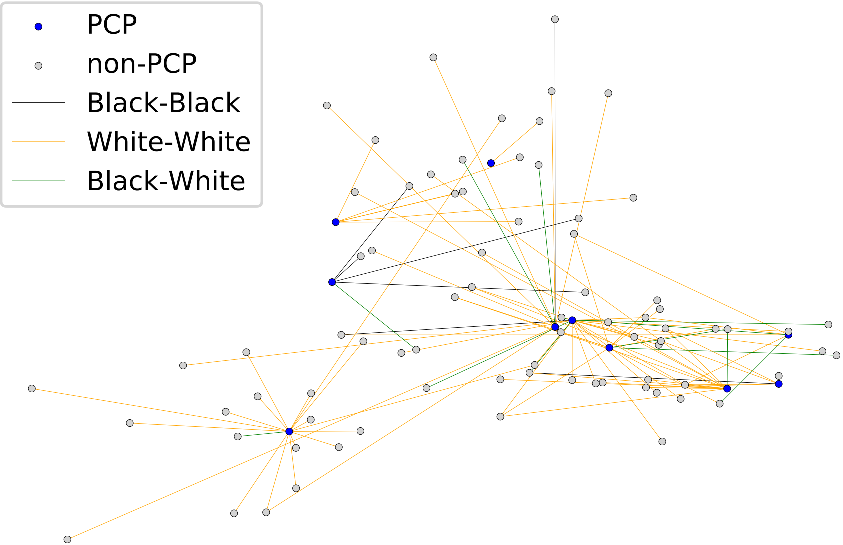

© 2021 Landon BE et al. JAMA Network Open.

HRR: 7; Specialty: 2; Sample: 3

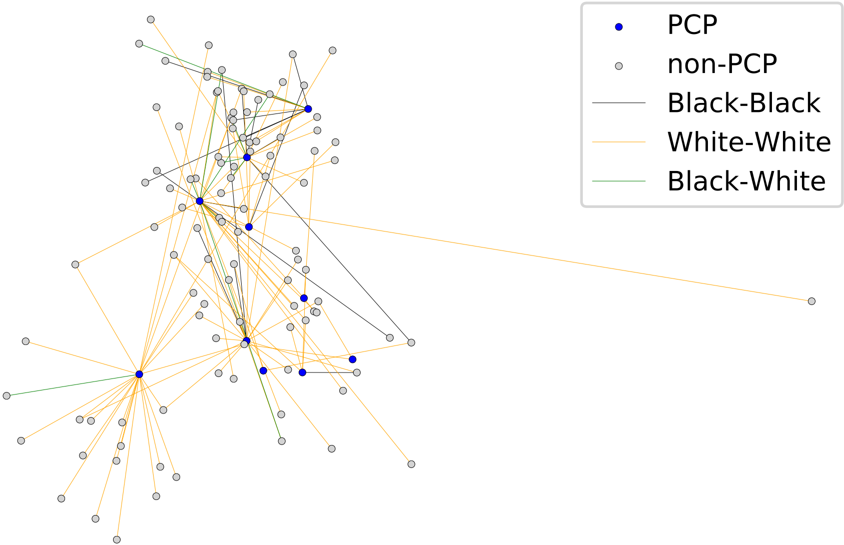

© 2021 Landon BE et al. JAMA Network Open.

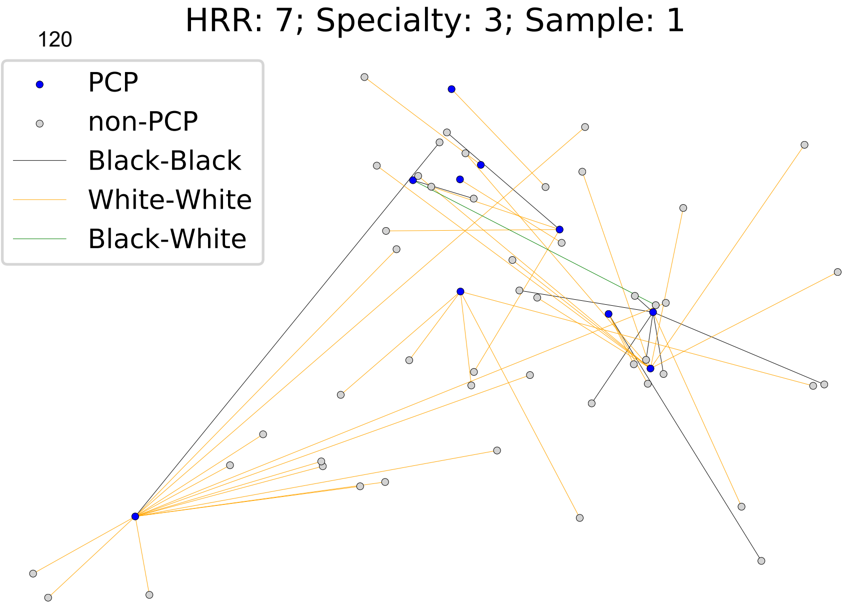

© 2021 Landon BE et al. JAMA Network Open.

HRR: 7; Specialty: 3; Sample: 2

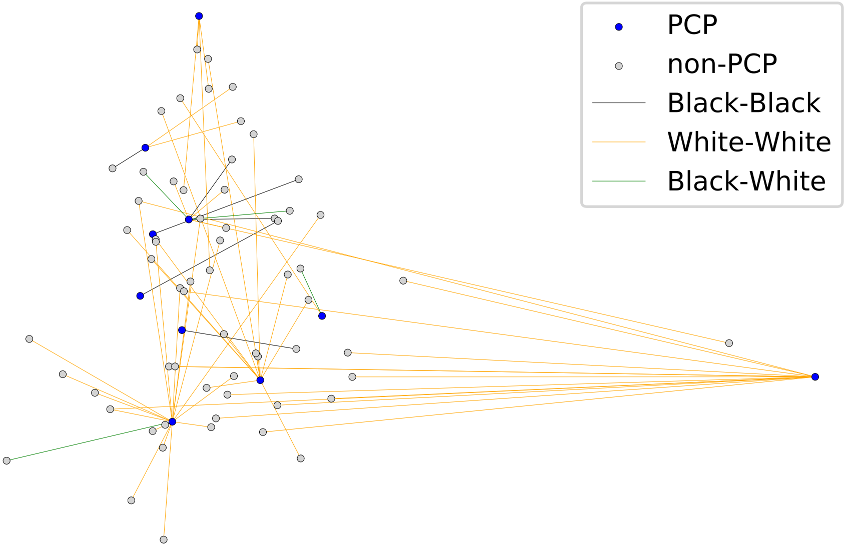

© 2021 Landon BE et al. JAMA Network Open.

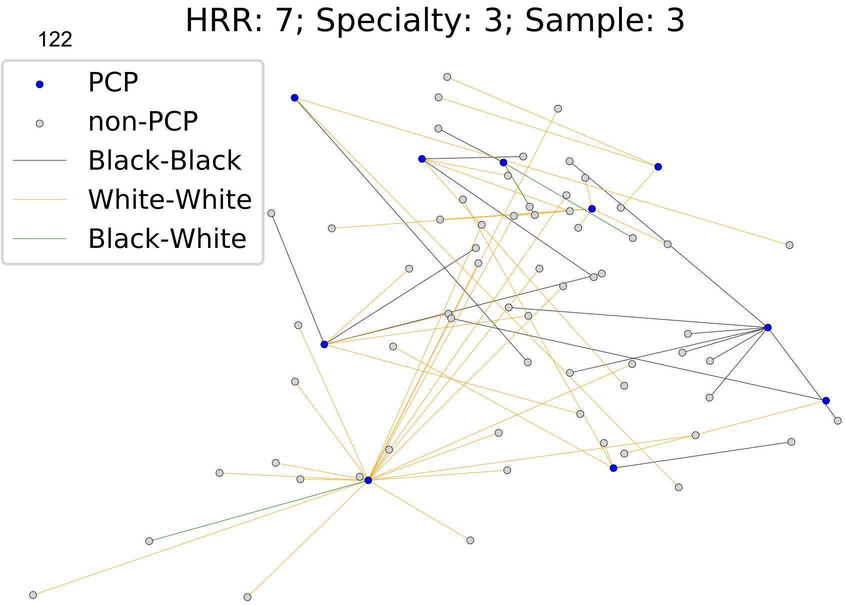

© 2021 Landon BE et al. JAMA Network Open.

HRR: 7; Specialty: 4; Sample: 1

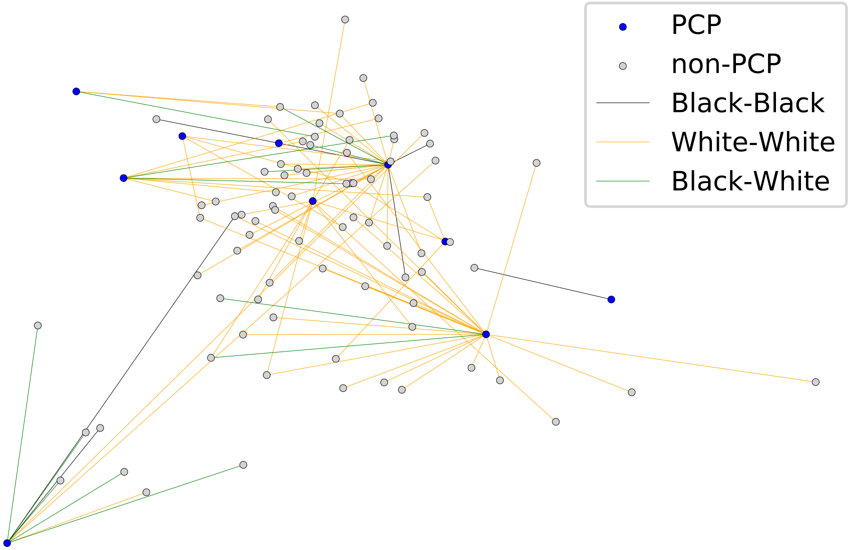

© 2021 Landon BE et al. JAMA Network Open.

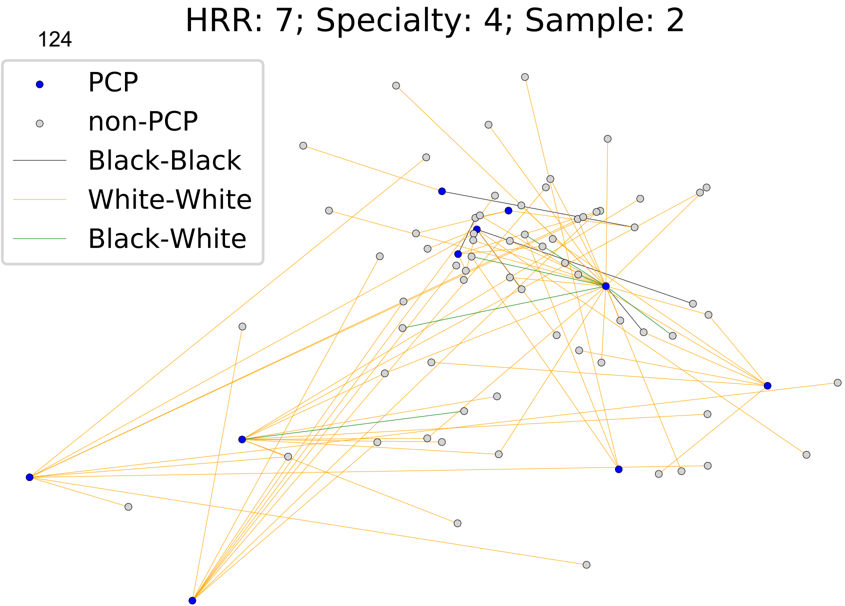

© 2021 Landon BE et al. JAMA Network Open.

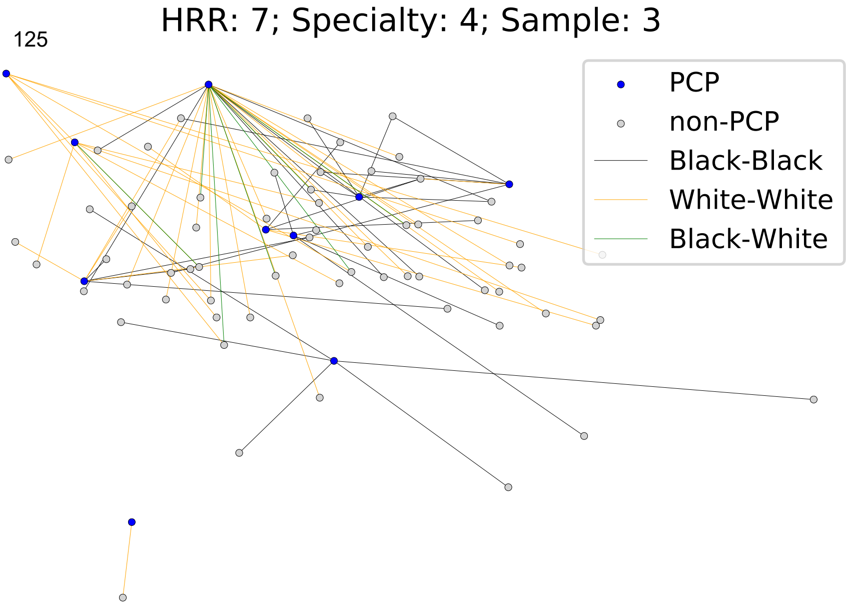

© 2021 Landon BE et al. JAMA Network Open.

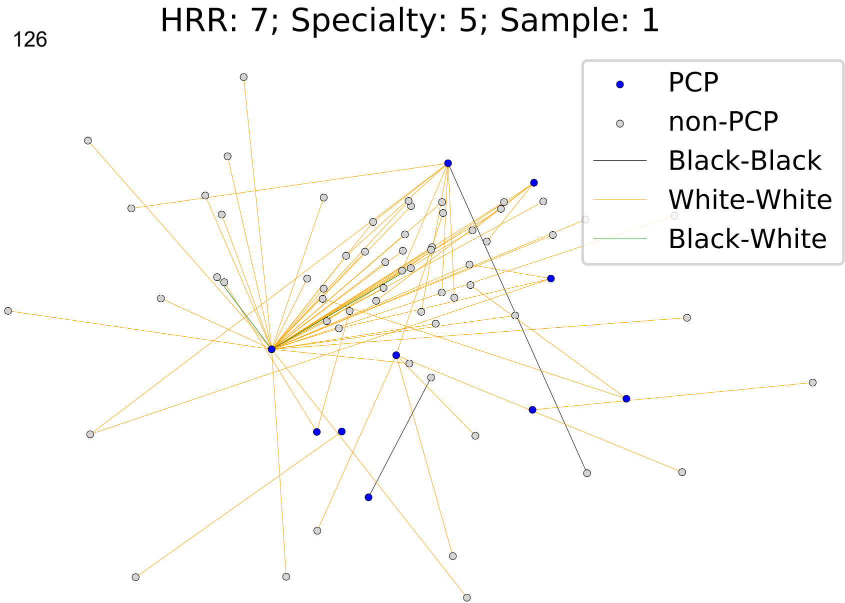

© 2021 Landon BE et al. JAMA Network Open.

HRR: 7; Specialty: 5; Sample: 2

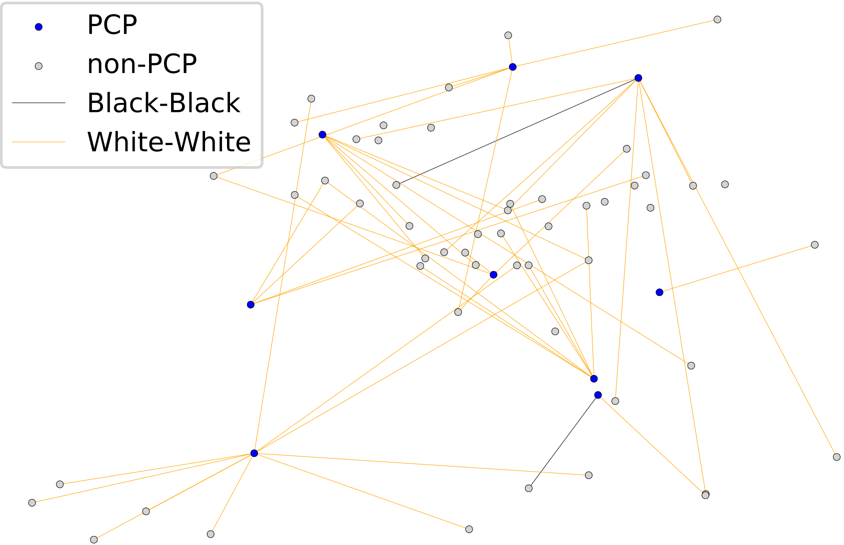

© 2021 Landon BE et al. JAMA Network Open.

HRR: 7; Specialty: 5; Sample: 3

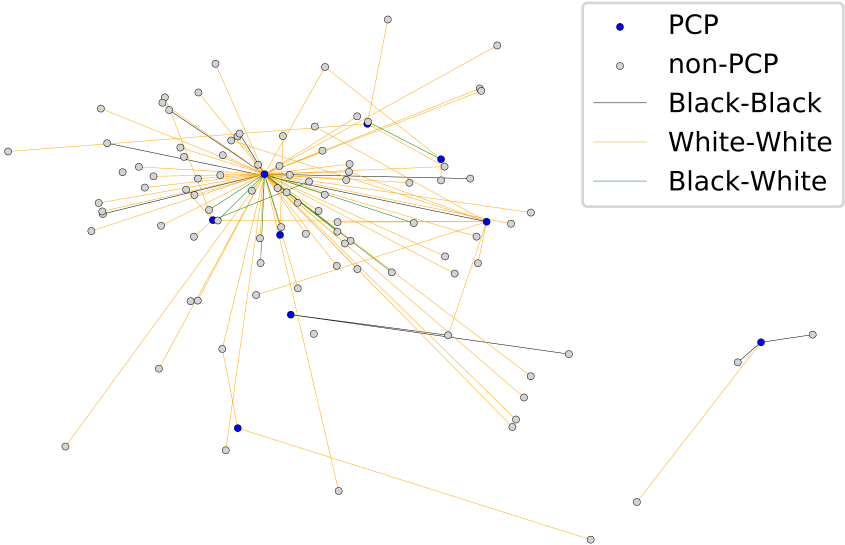

© 2021 Landon BE et al. JAMA Network Open.

HRR: 7; Specialty: 6; Sample: 1

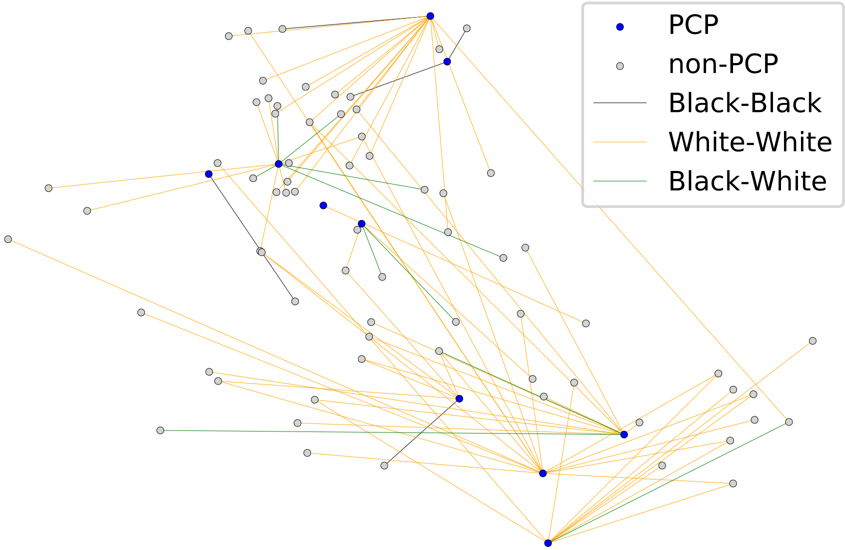

© 2021 Landon BE et al. JAMA Network Open.

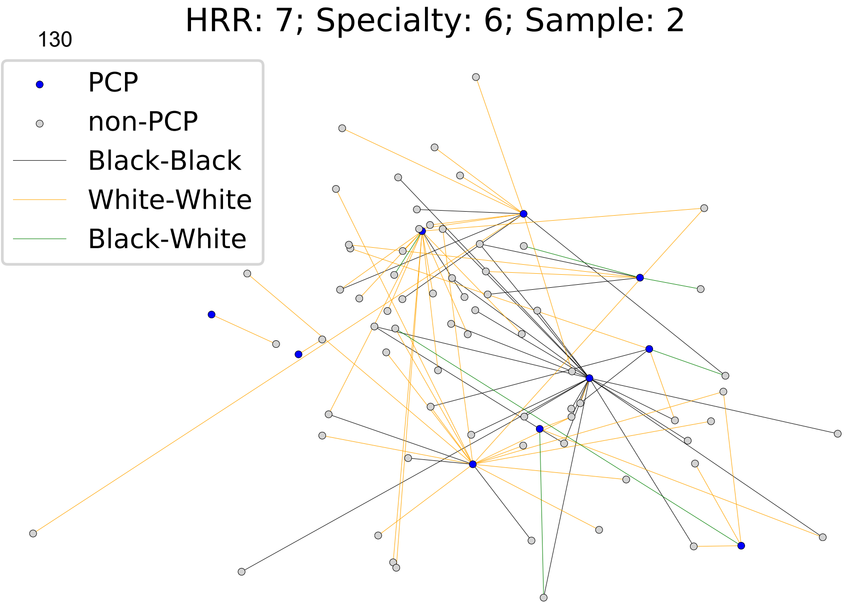

© 2021 Landon BE et al. JAMA Network Open.

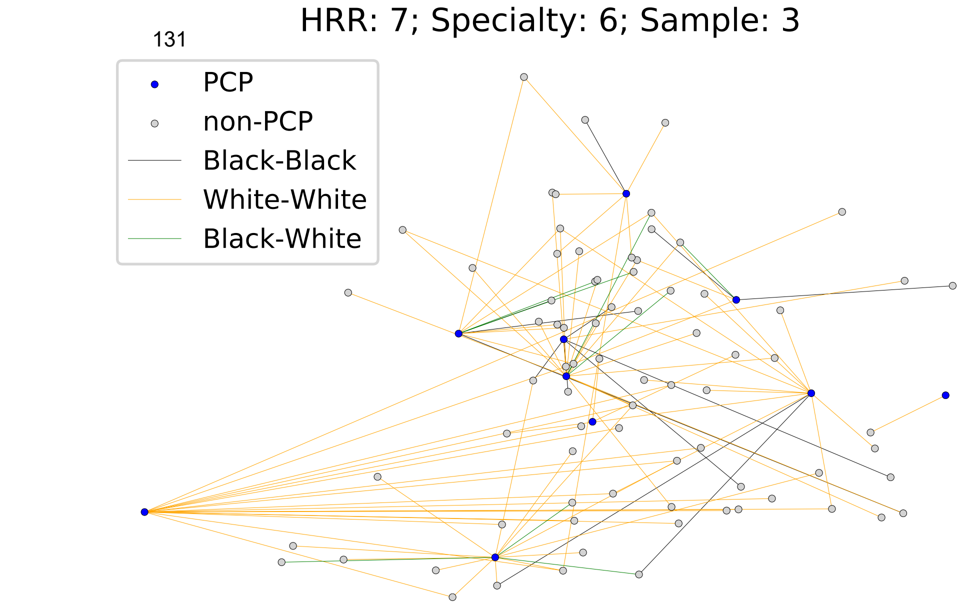

© 2021 Landon BE et al. JAMA Network Open.

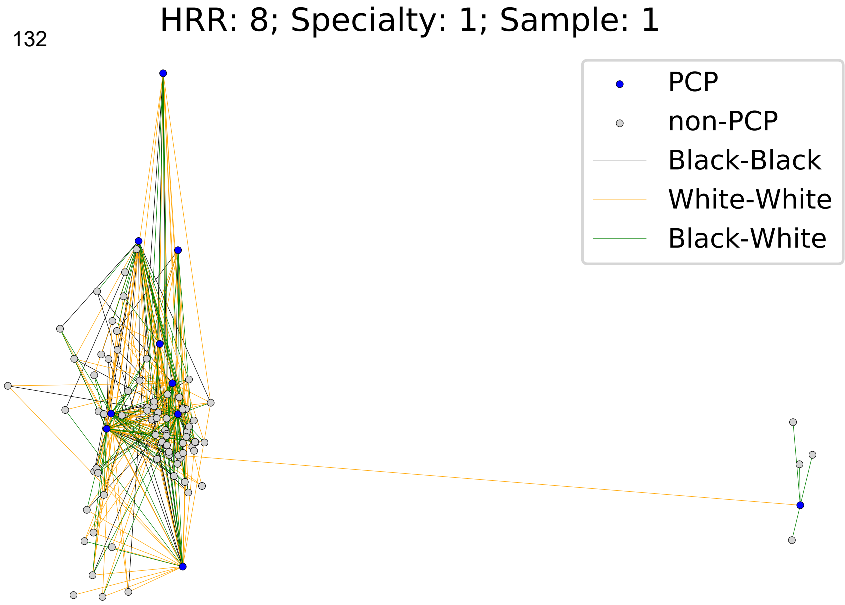

© 2021 Landon BE et al. JAMA Network Open.

HRR: 8; Specialty: 1; Sample: 2

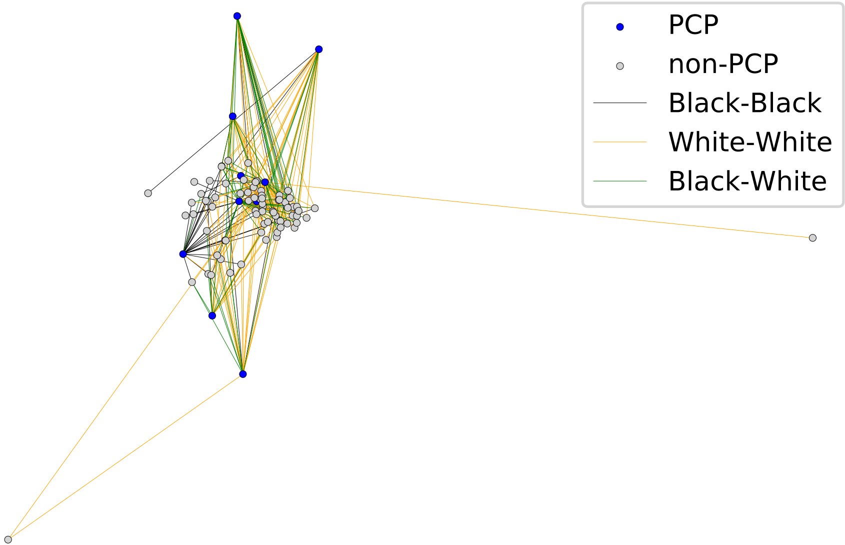

© 2021 Landon BE et al. JAMA Network Open.

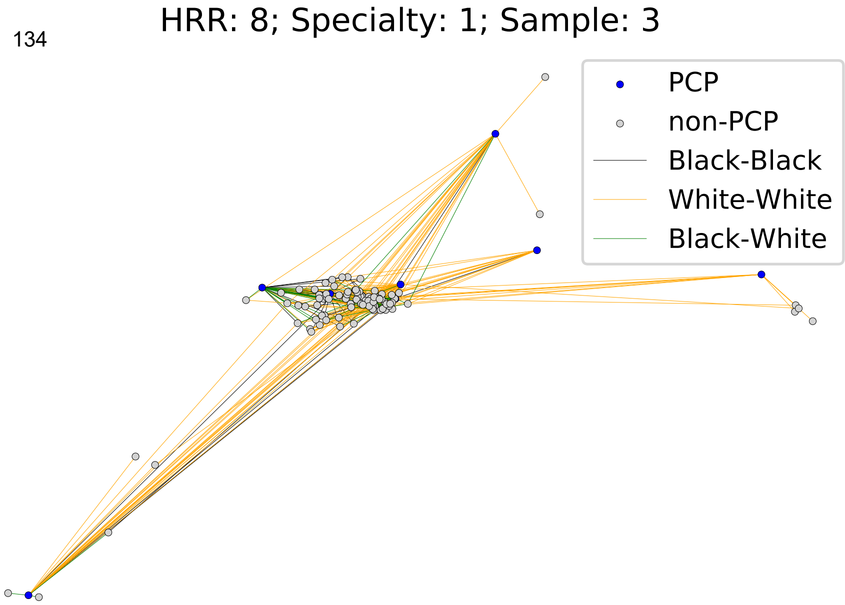

© 2021 Landon BE et al. JAMA Network Open.

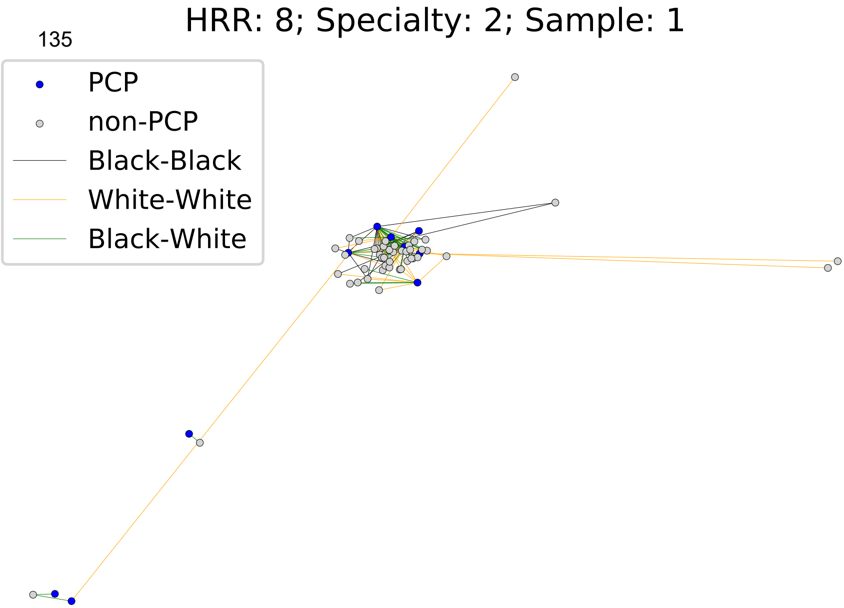

© 2021 Landon BE et al. JAMA Network Open.

HRR: 8; Specialty: 2; Sample: 2

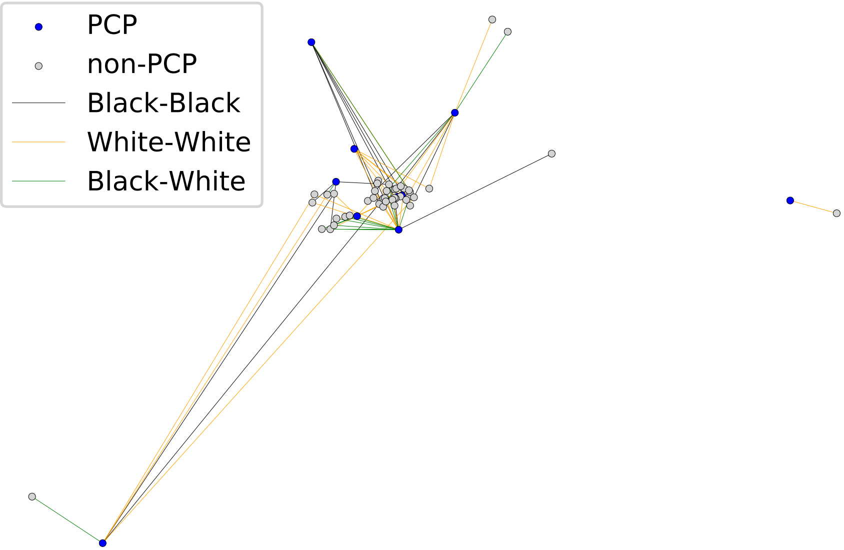

© 2021 Landon BE et al. JAMA Network Open.

HRR: 8; Specialty: 2; Sample: 3

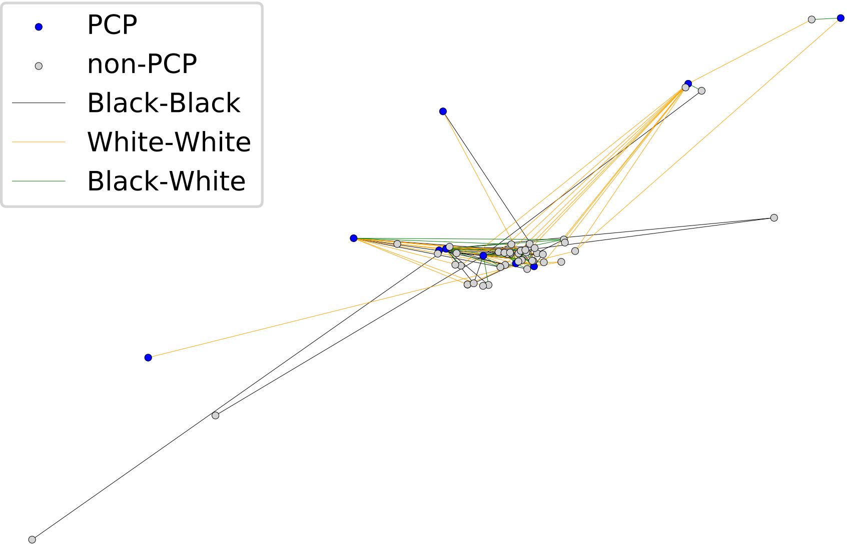

© 2021 Landon BE et al. JAMA Network Open.

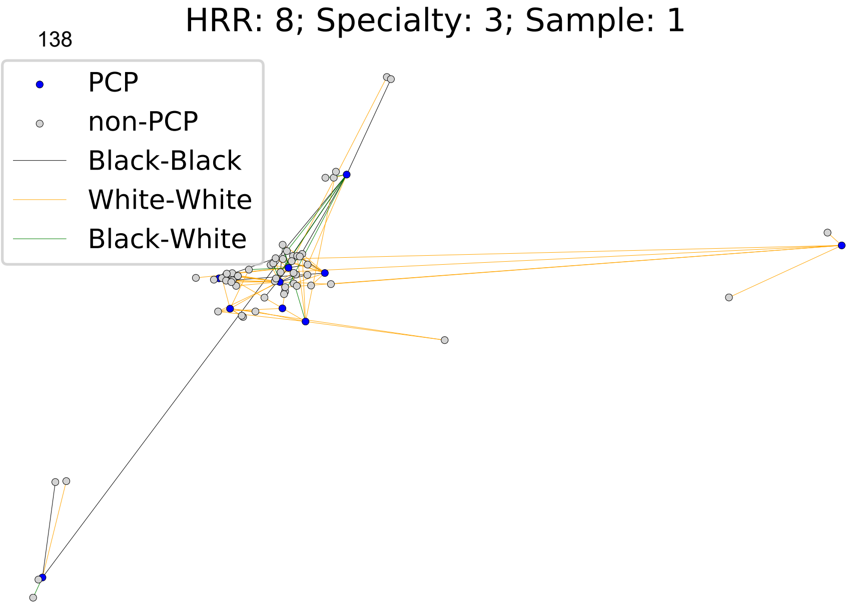

© 2021 Landon BE et al. JAMA Network Open.

HRR: 8; Specialty: 3; Sample: 2

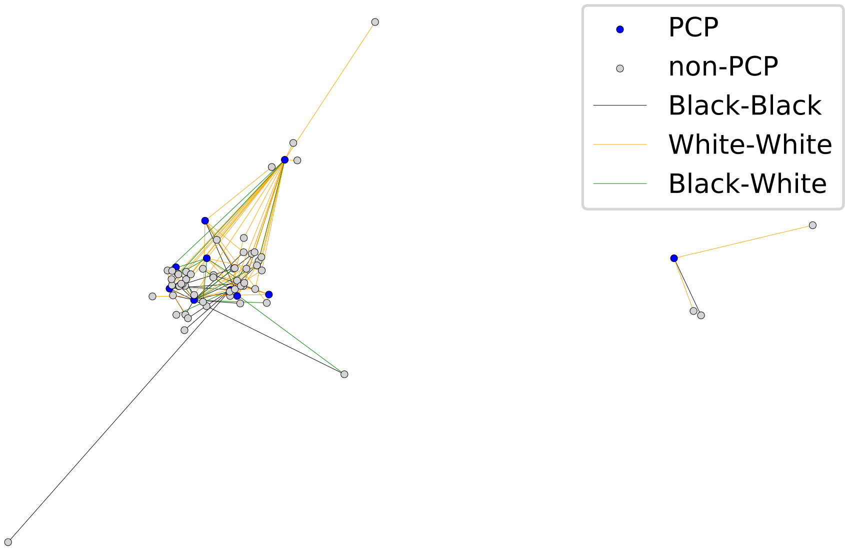

© 2021 Landon BE et al. JAMA Network Open.

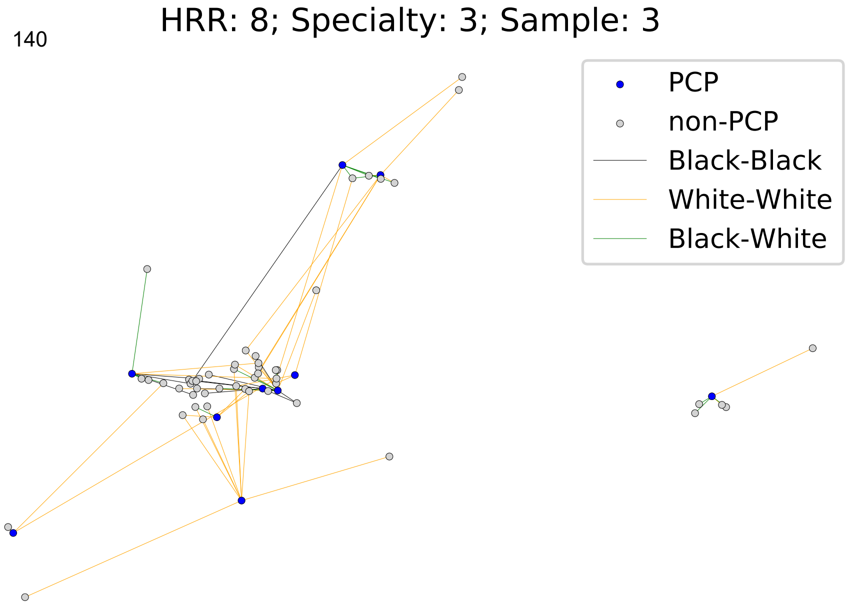

© 2021 Landon BE et al. JAMA Network Open.

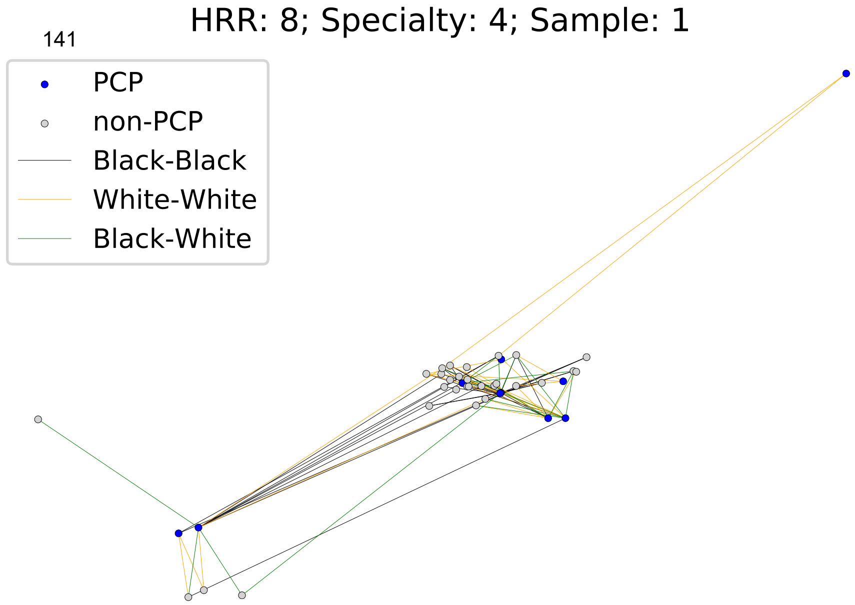

© 2021 Landon BE et al. JAMA Network Open.

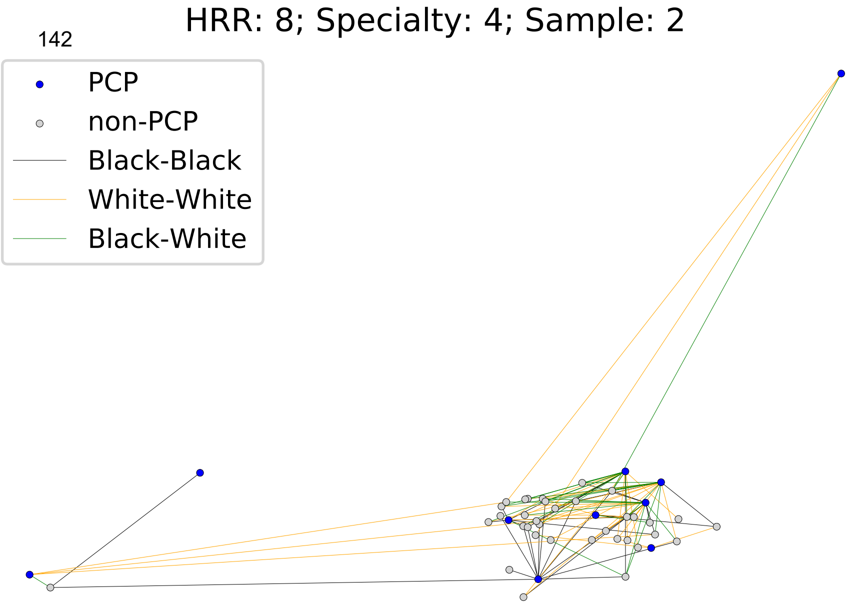

© 2021 Landon BE et al. JAMA Network Open.

HRR: 8; Specialty: 4; Sample: 3

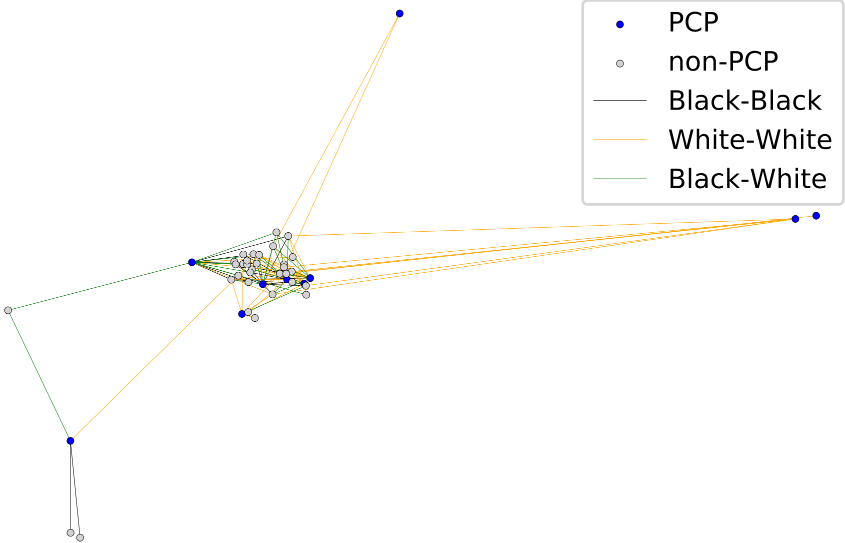

© 2021 Landon BE et al. JAMA Network Open.

HRR: 8; Specialty: 5; Sample: 1

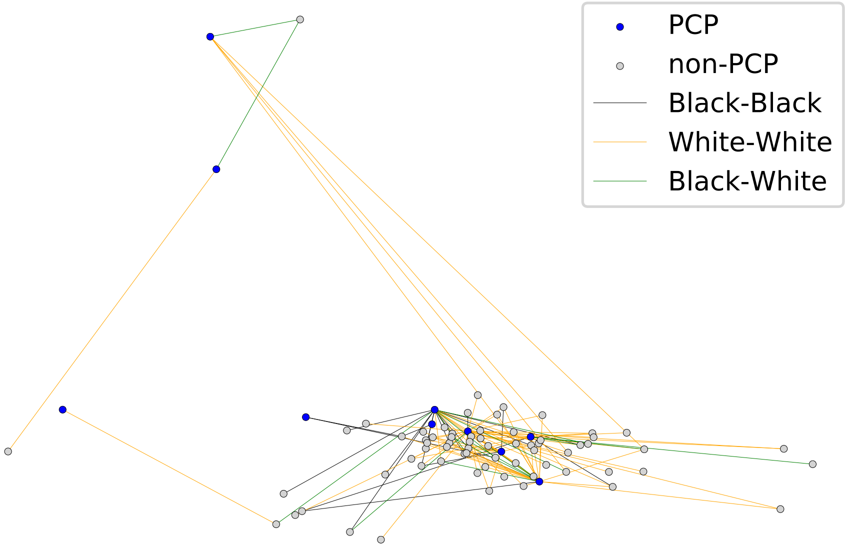

© 2021 Landon BE et al. JAMA Network Open.

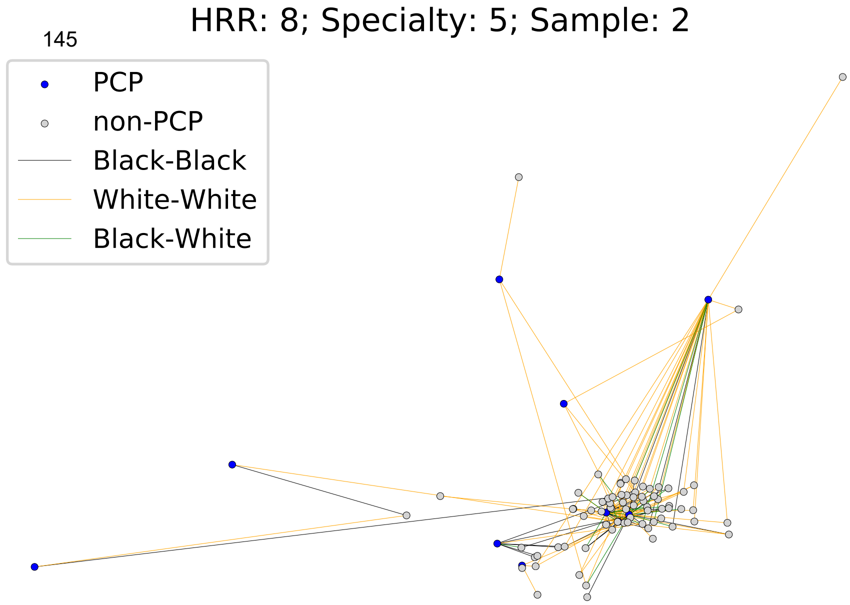

© 2021 Landon BE et al. JAMA Network Open.

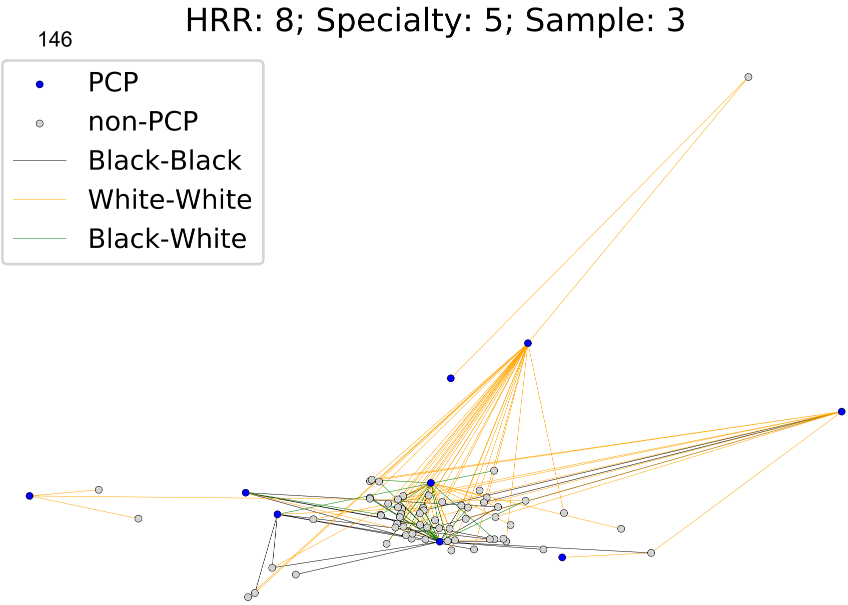

© 2021 Landon BE et al. JAMA Network Open.

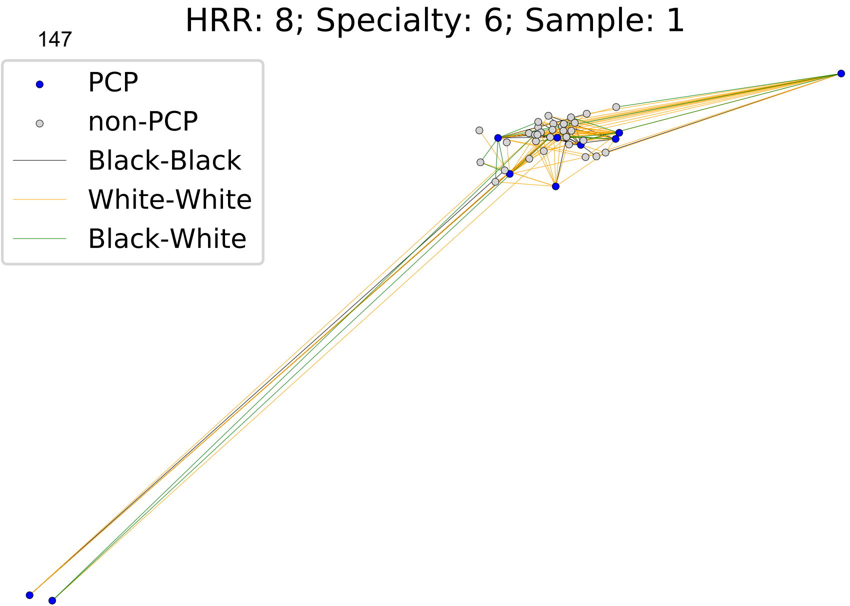

© 2021 Landon BE et al. JAMA Network Open.

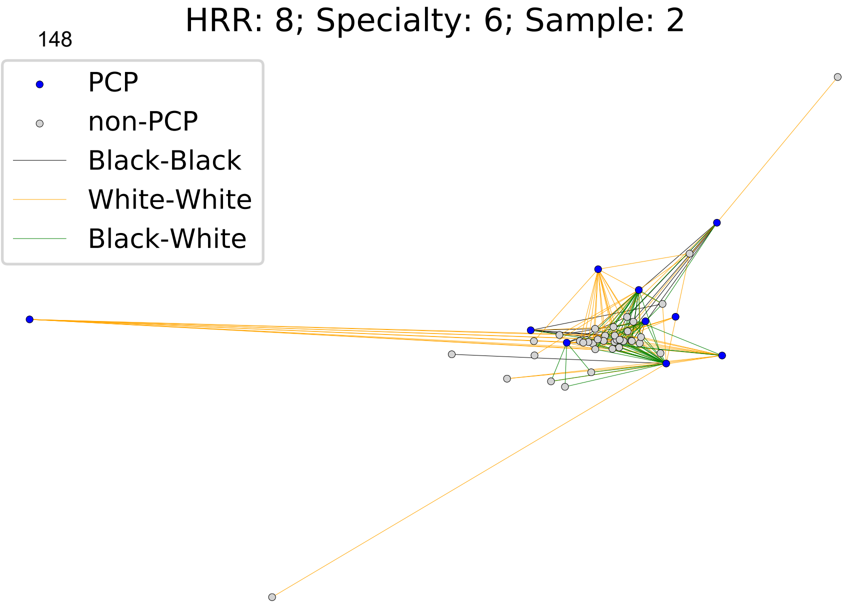

© 2021 Landon BE et al. JAMA Network Open.

HRR: 8; Specialty: 6; Sample: 3

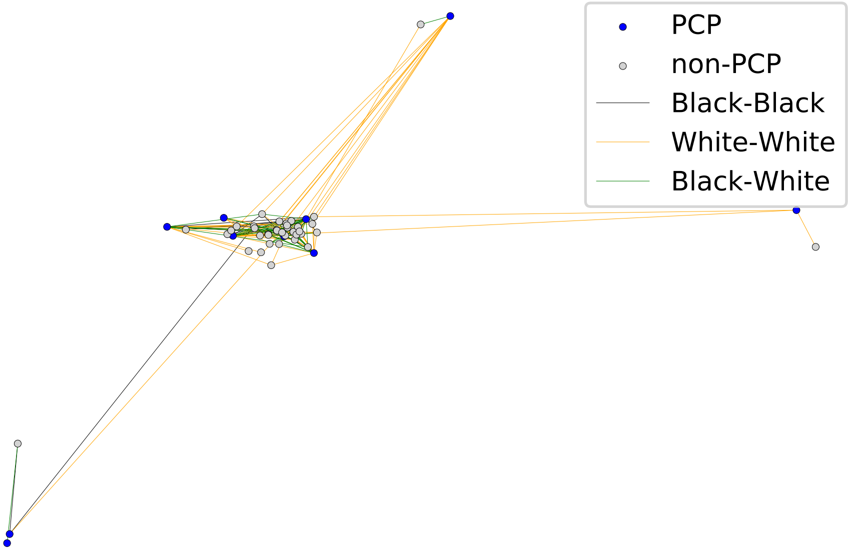

© 2021 Landon BE et al. JAMA Network Open.

HRR: 9; Specialty: 1; Sample: 1

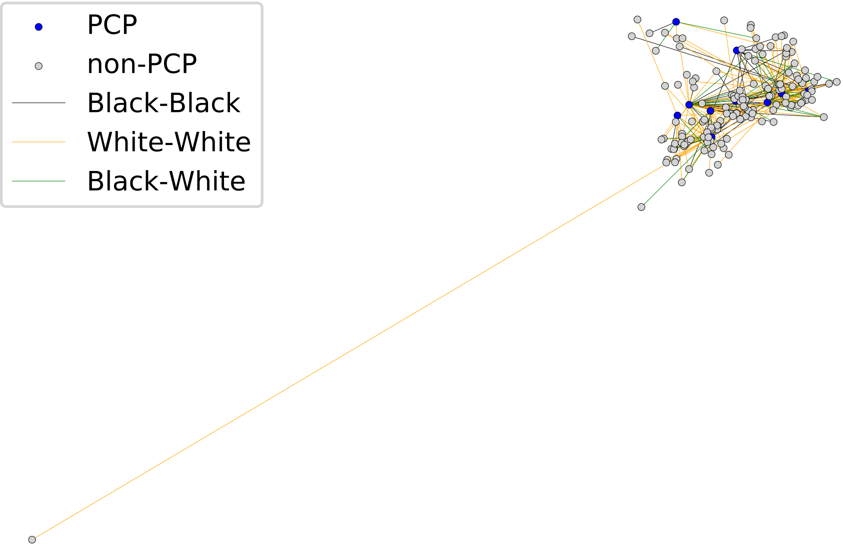

© 2021 Landon BE et al. JAMA Network Open.

HRR: 9; Specialty: 1; Sample: 2

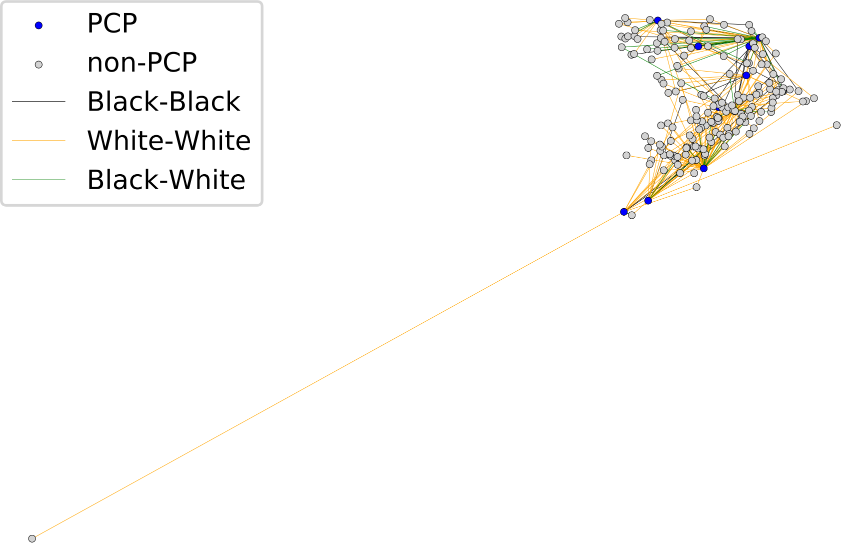

© 2021 Landon BE et al. JAMA Network Open.

HRR: 9; Specialty: 1; Sample: 3

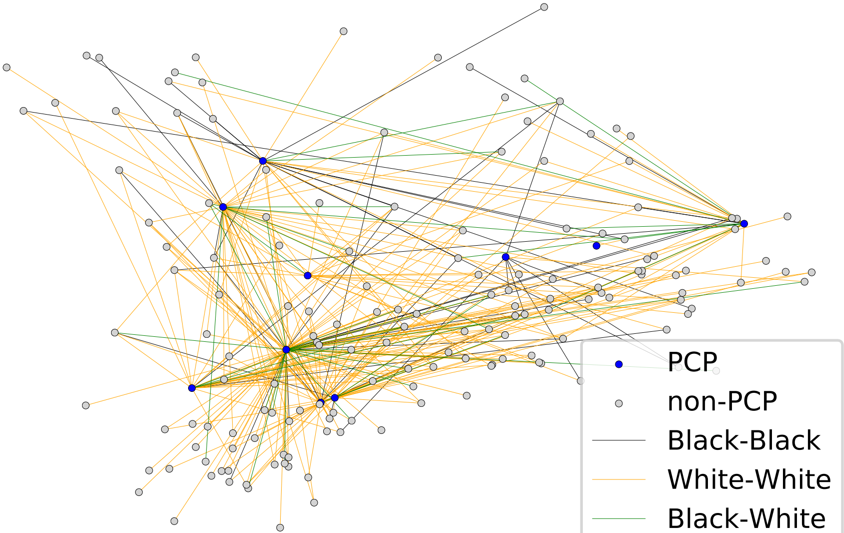

© 2021 Landon BE et al. JAMA Network Open.

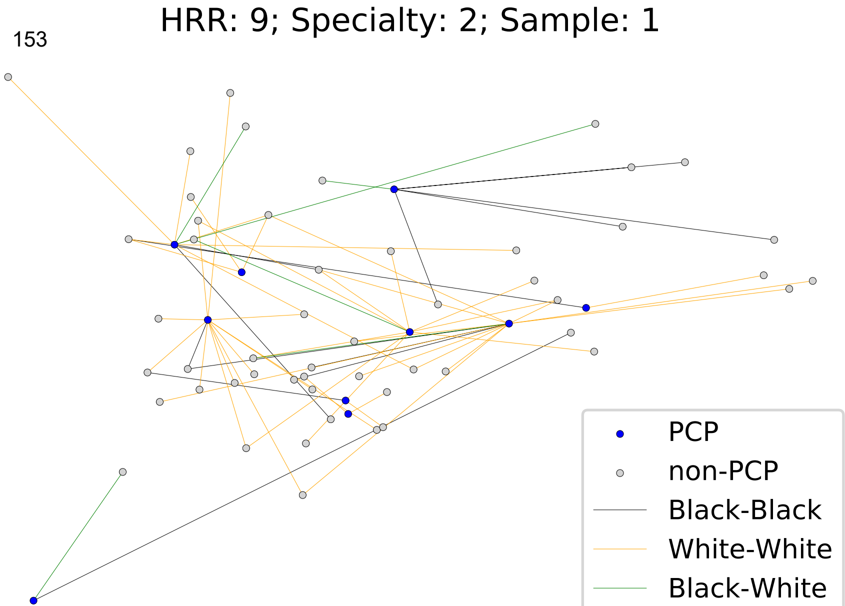

© 2021 Landon BE et al. JAMA Network Open.

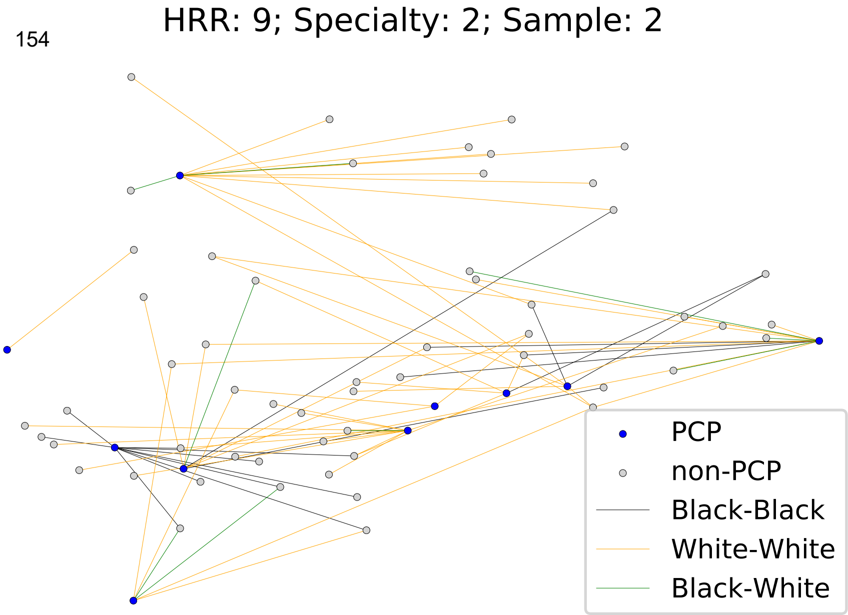

© 2021 Landon BE et al. JAMA Network Open.

HRR: 9; Specialty: 2; Sample: 3

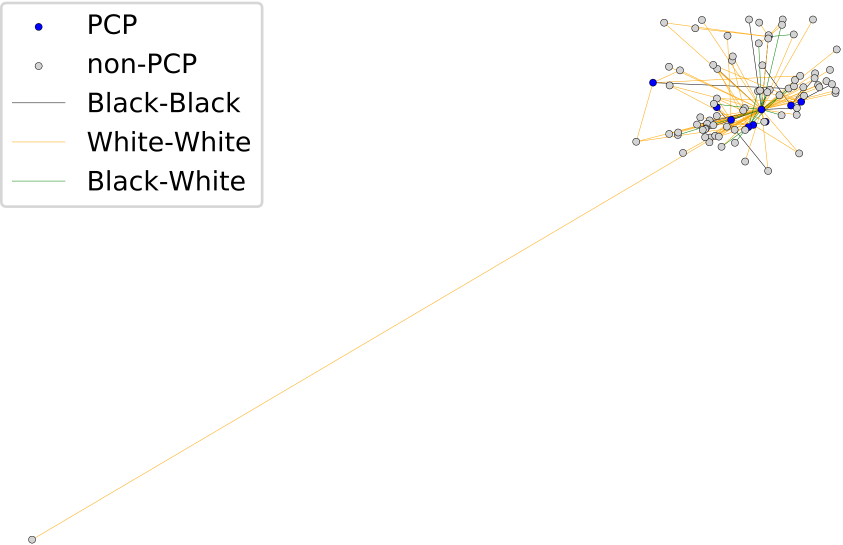

© 2021 Landon BE et al. JAMA Network Open.

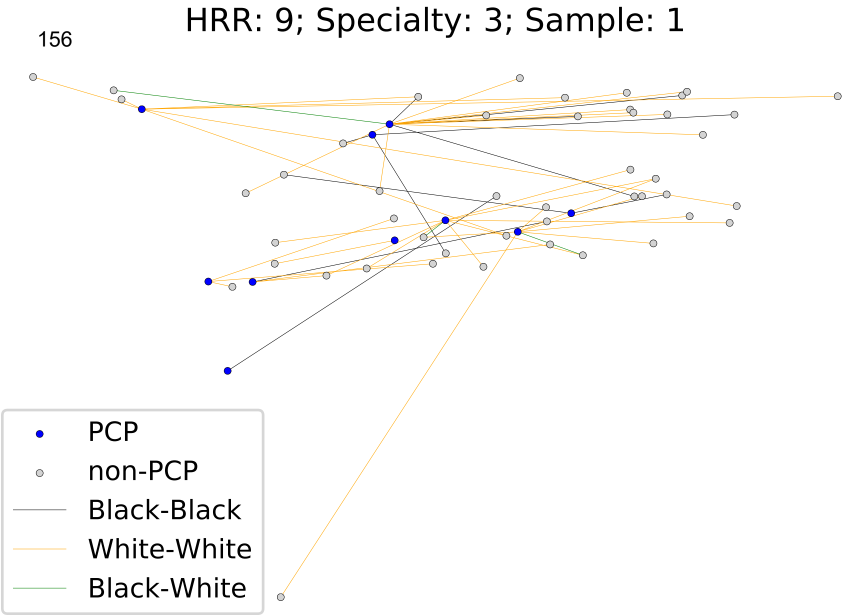

© 2021 Landon BE et al. JAMA Network Open.

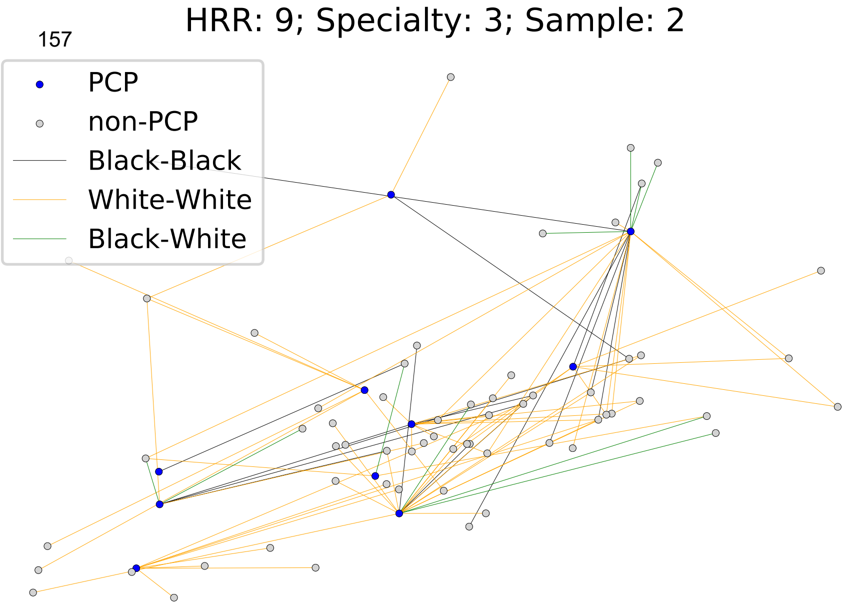

© 2021 Landon BE et al. JAMA Network Open.

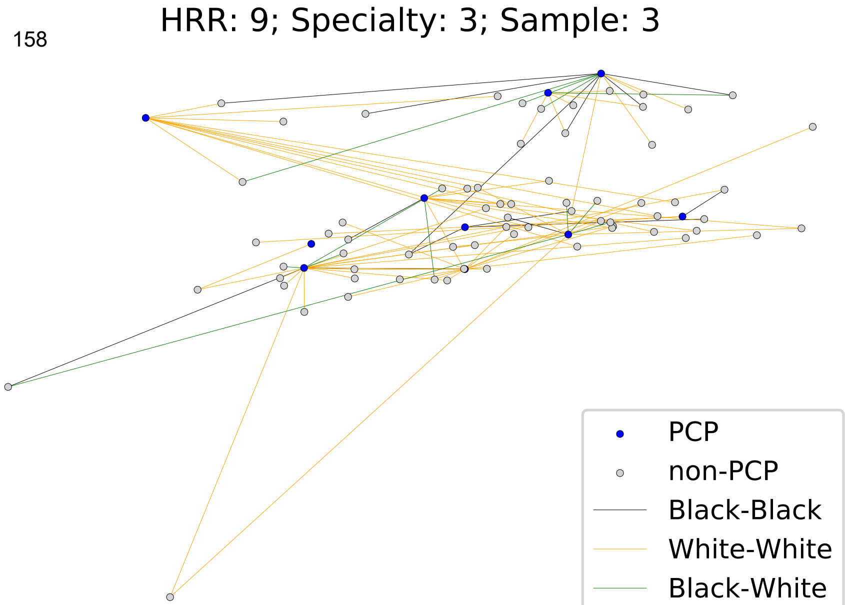

© 2021 Landon BE et al. JAMA Network Open.

HRR: 9; Specialty: 4; Sample: 1

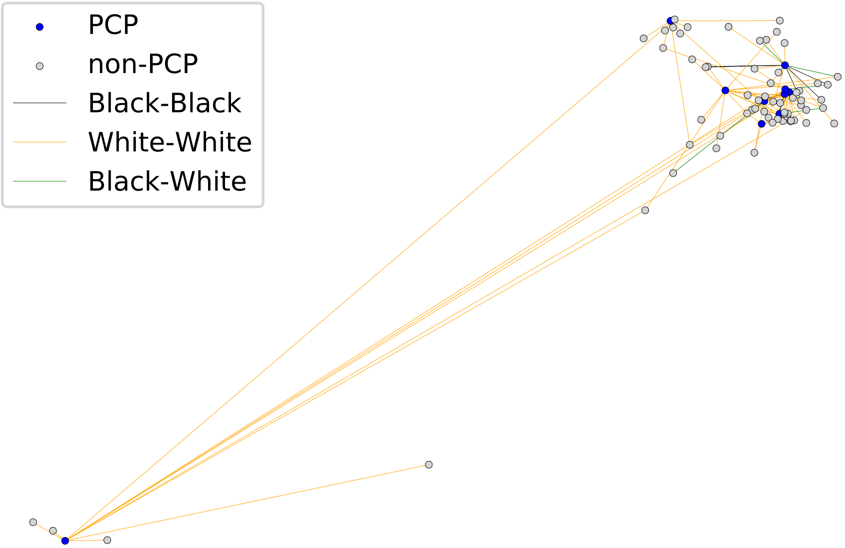

© 2021 Landon BE et al. JAMA Network Open.

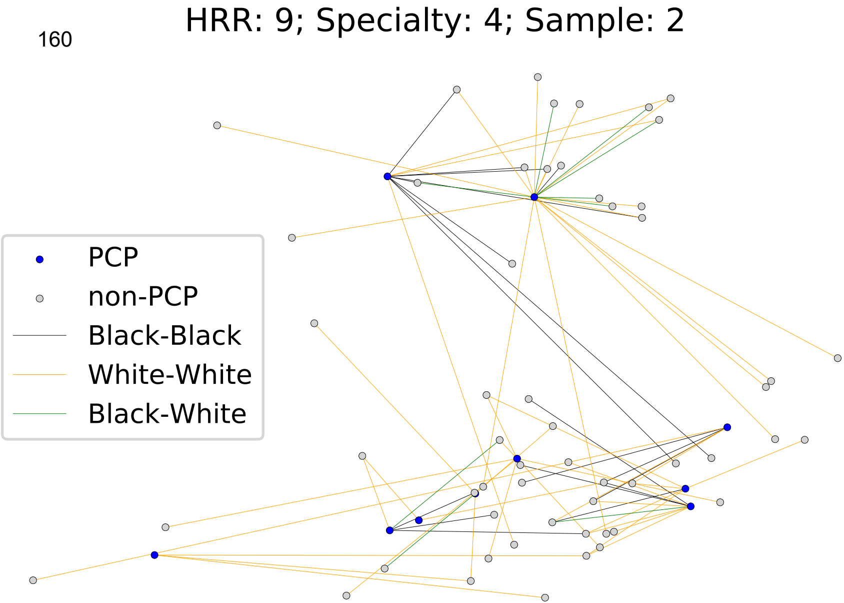

© 2021 Landon BE et al. JAMA Network Open.

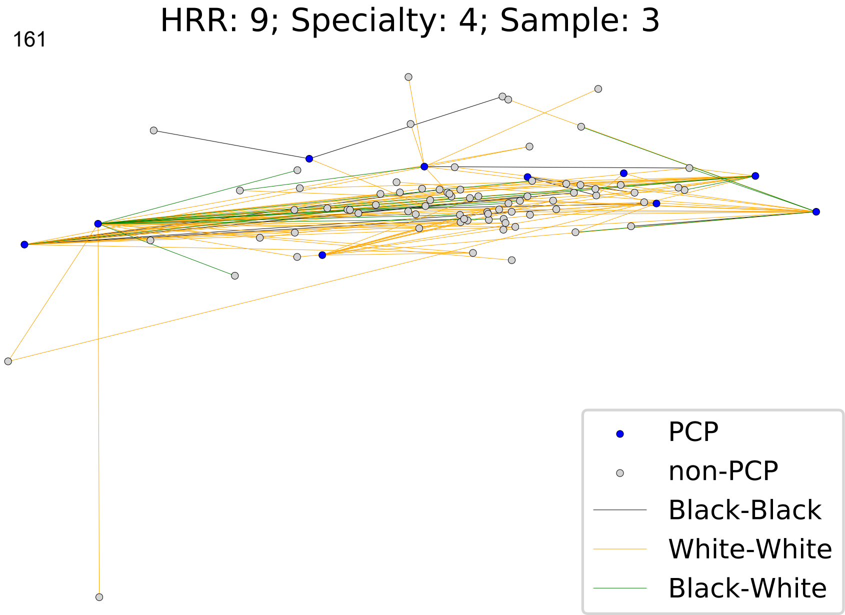

© 2021 Landon BE et al. JAMA Network Open.

HRR: 9; Specialty: 5; Sample: 1

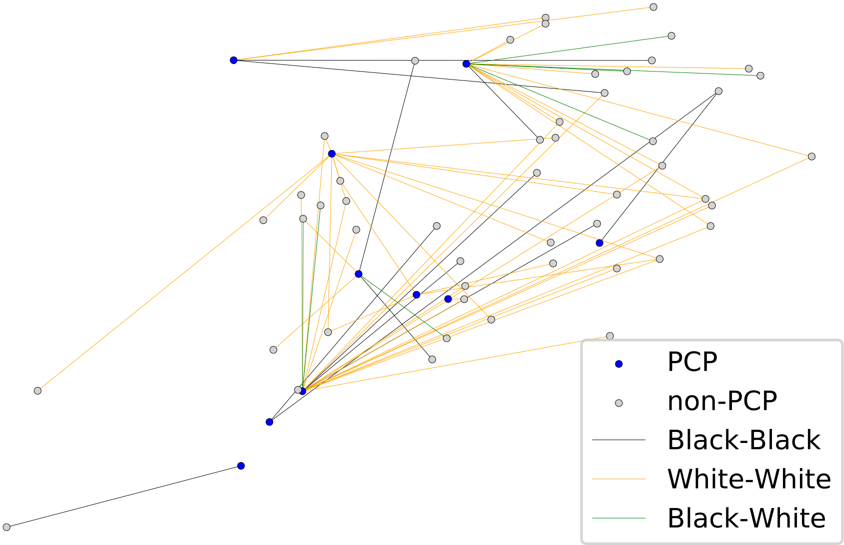

© 2021 Landon BE et al. JAMA Network Open.

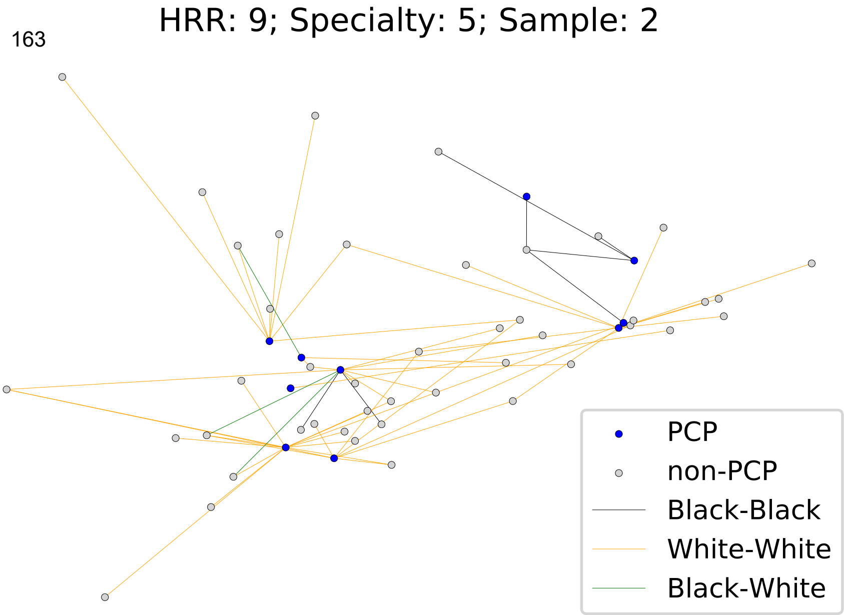

© 2021 Landon BE et al. JAMA Network Open.

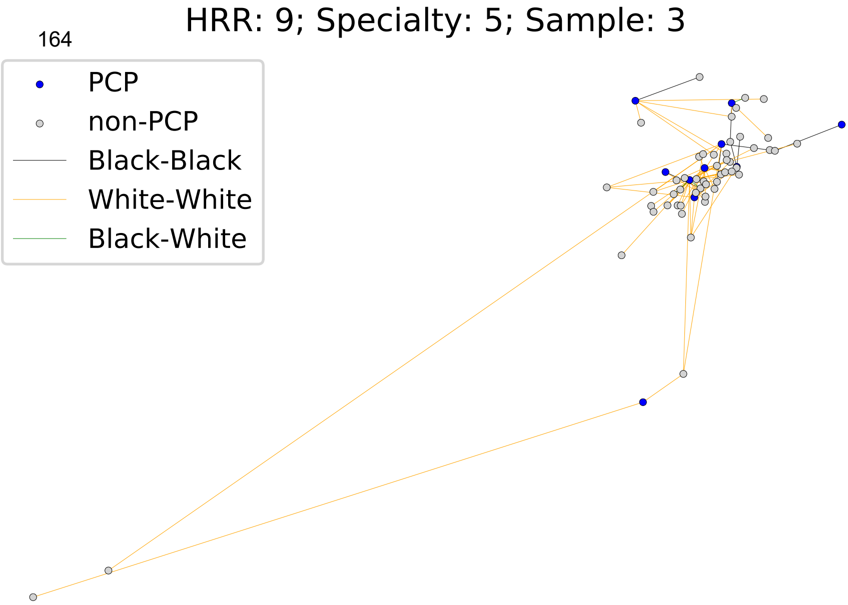

© 2021 Landon BE et al. JAMA Network Open.

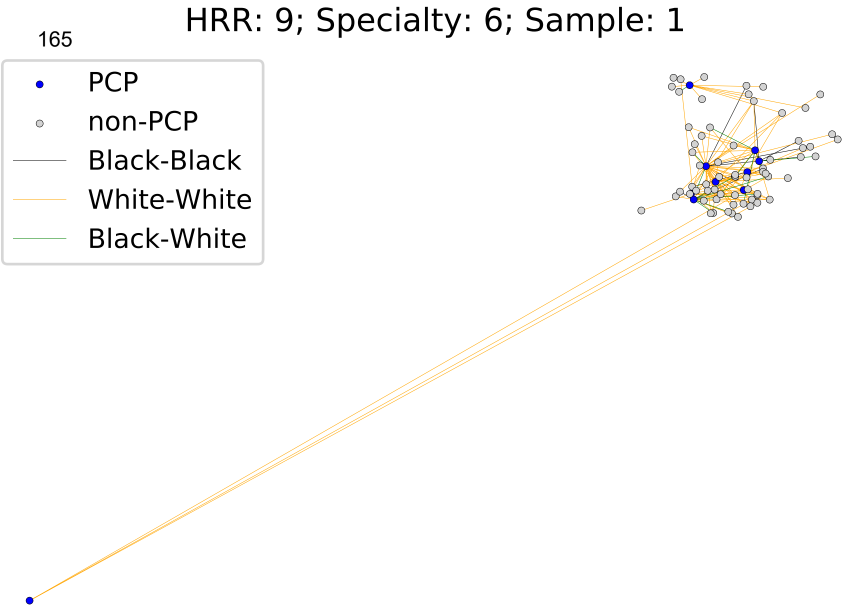

© 2021 Landon BE et al. JAMA Network Open.

HRR: 9; Specialty: 6; Sample: 2

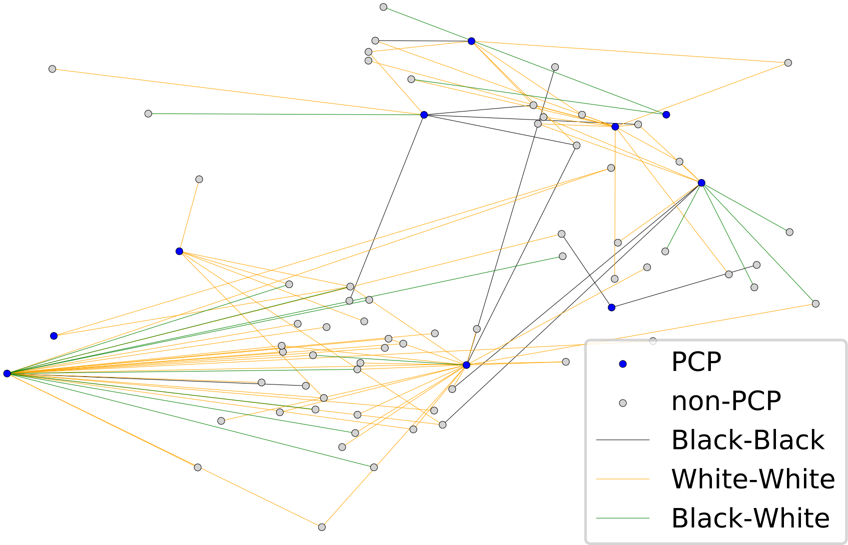

© 2021 Landon BE et al. JAMA Network Open.

HRR: 9; Specialty: 6; Sample: 3

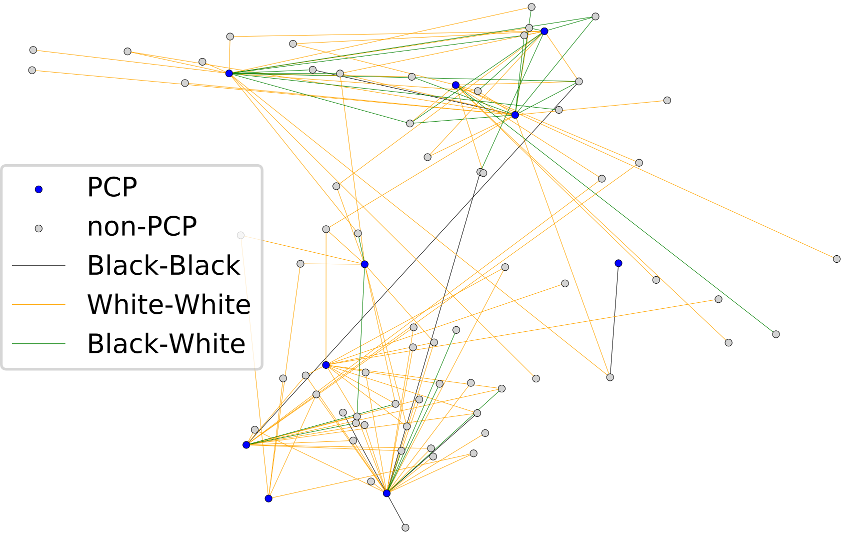

© 2021 Landon BE et al. JAMA Network Open.

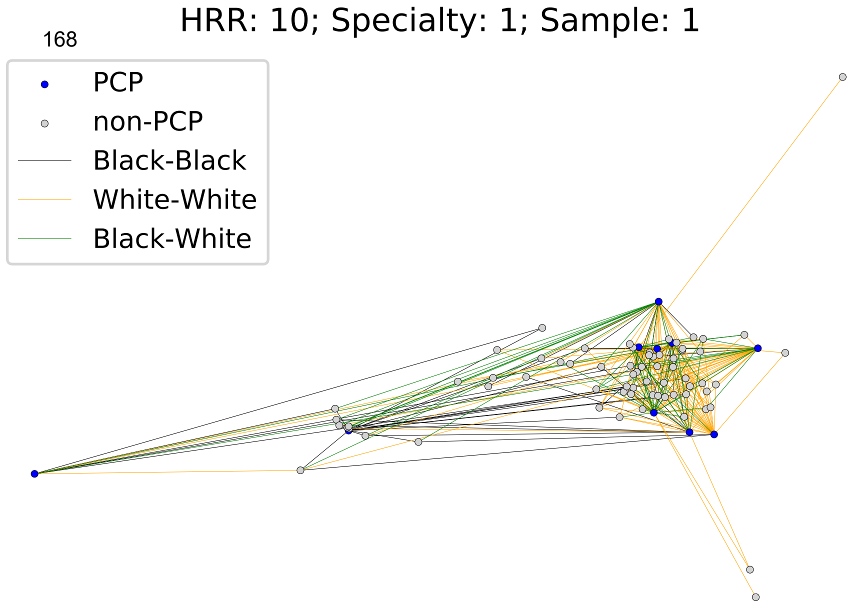

© 2021 Landon BE et al. JAMA Network Open.

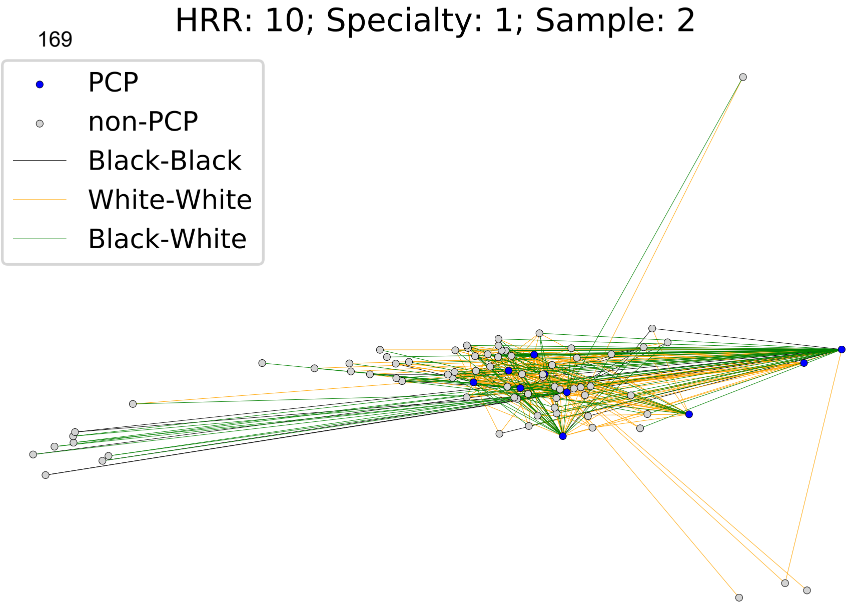

© 2021 Landon BE et al. JAMA Network Open.

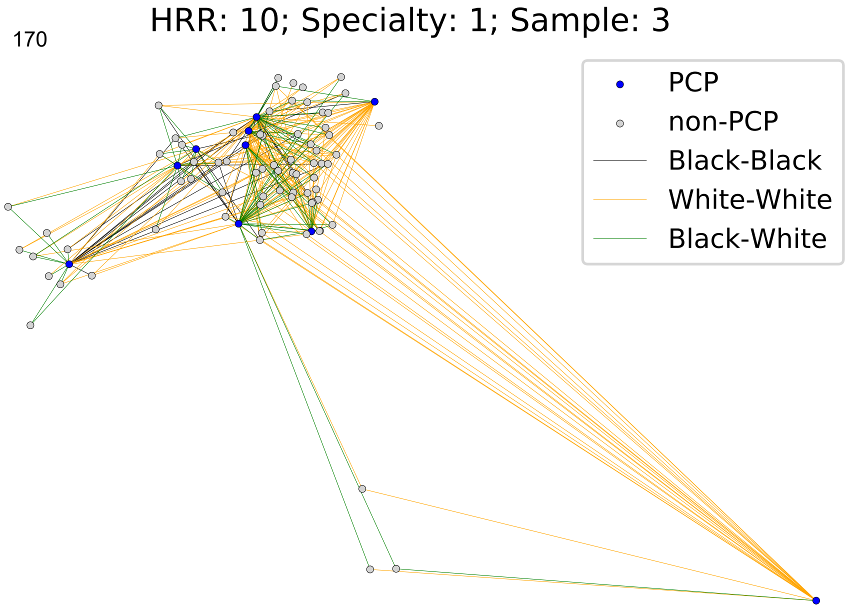

© 2021 Landon BE et al. JAMA Network Open.

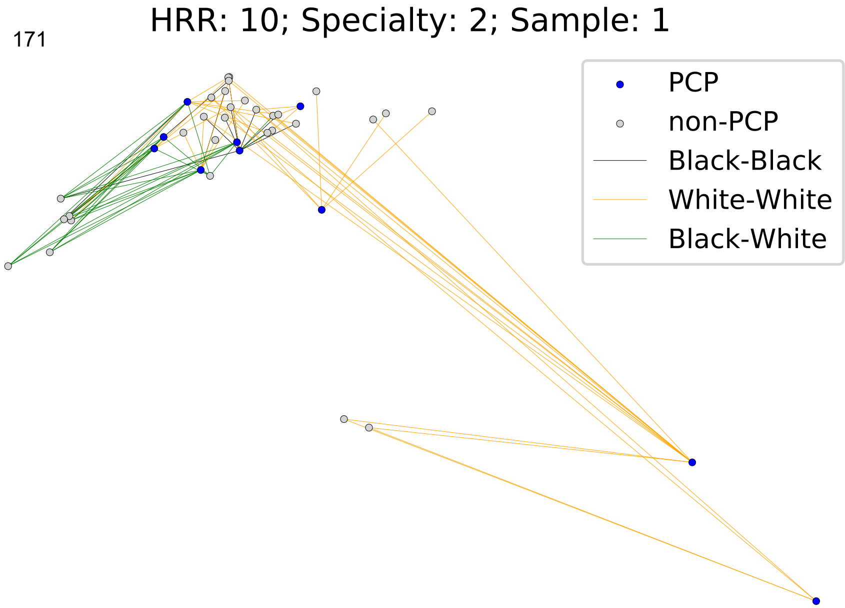

© 2021 Landon BE et al. JAMA Network Open.

HRR: 10; Specialty: 2; Sample: 2

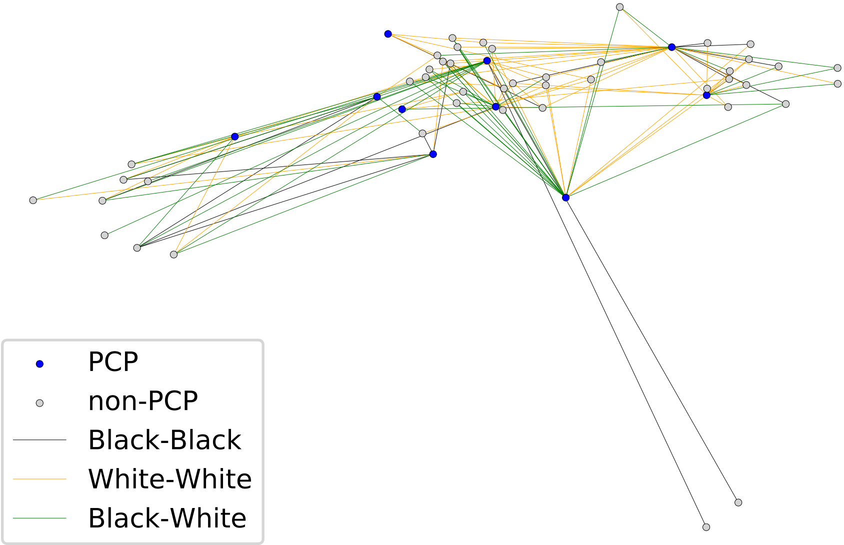

© 2021 Landon BE et al. JAMA Network Open.

HRR: 10; Specialty: 2; Sample: 3

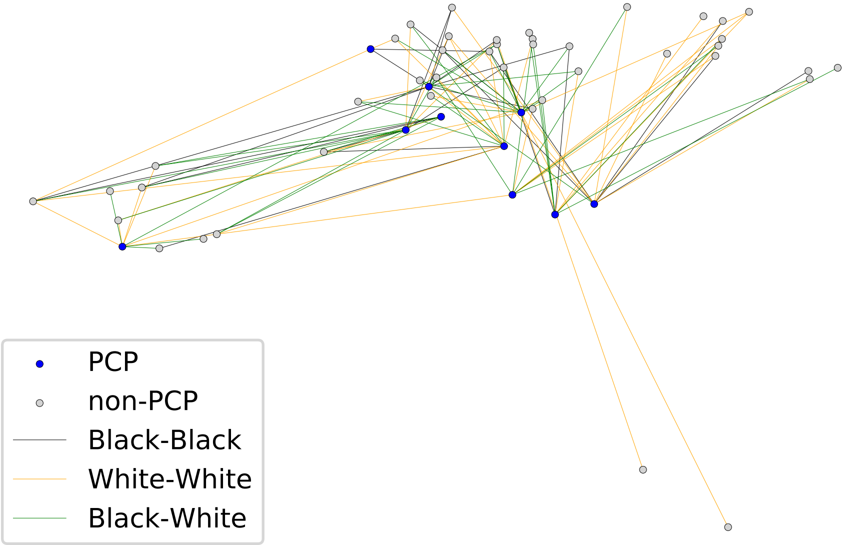

© 2021 Landon BE et al. JAMA Network Open.

HRR: 10; Specialty: 3; Sample: 1

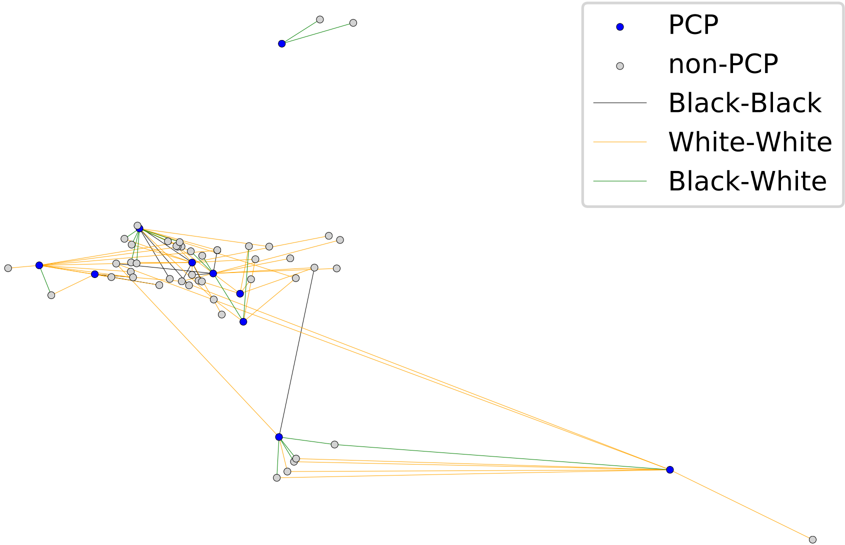

© 2021 Landon BE et al. JAMA Network Open.

HRR: 10; Specialty: 3; Sample: 2

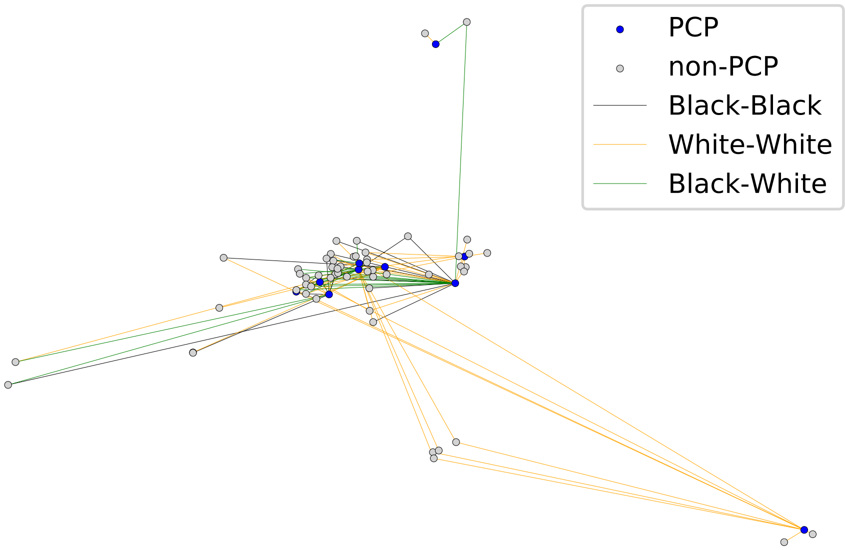

© 2021 Landon BE et al. JAMA Network Open.

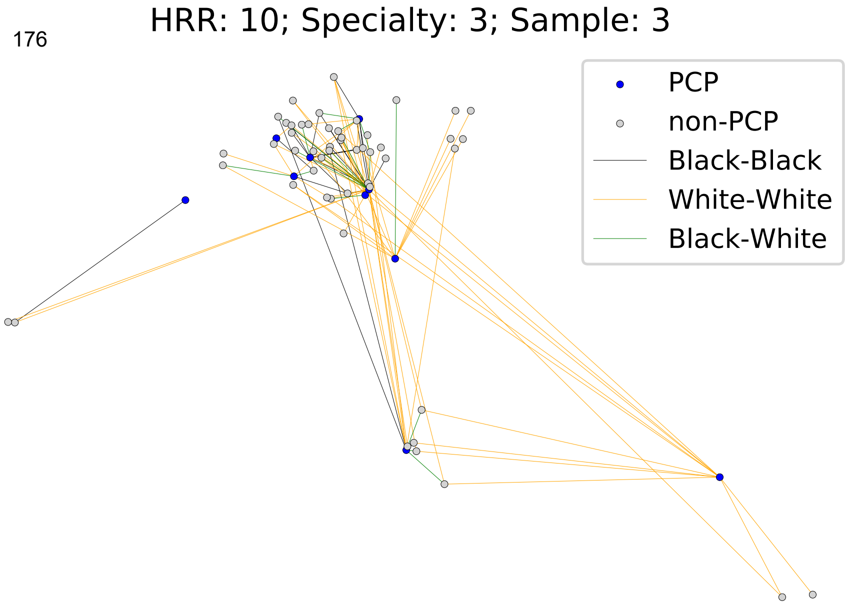

© 2021 Landon BE et al. JAMA Network Open.

HRR: 10; Specialty: 4; Sample: 1

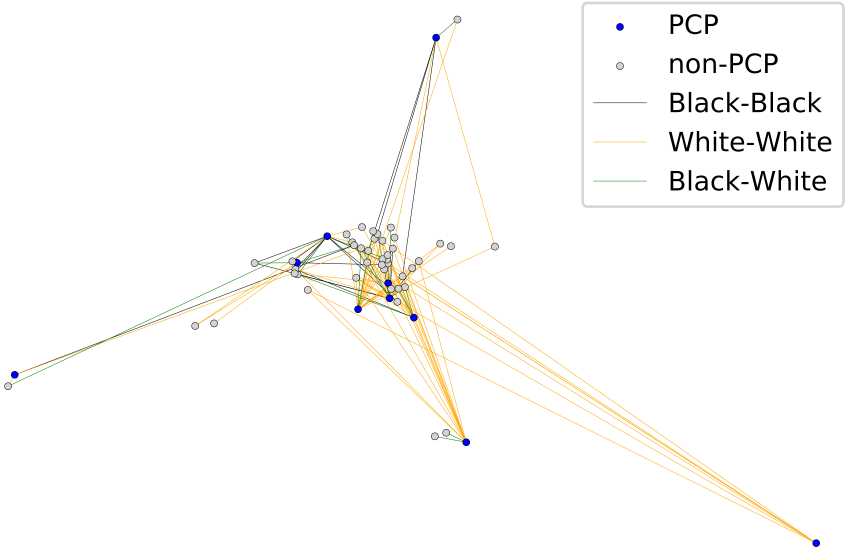

© 2021 Landon BE et al. JAMA Network Open.

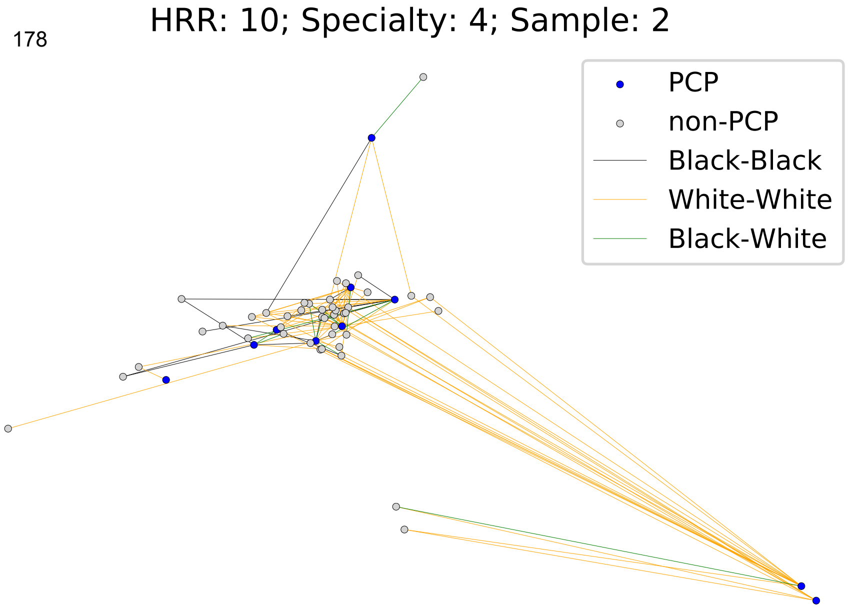

© 2021 Landon BE et al. JAMA Network Open.

HRR: 10; Specialty: 4; Sample: 3

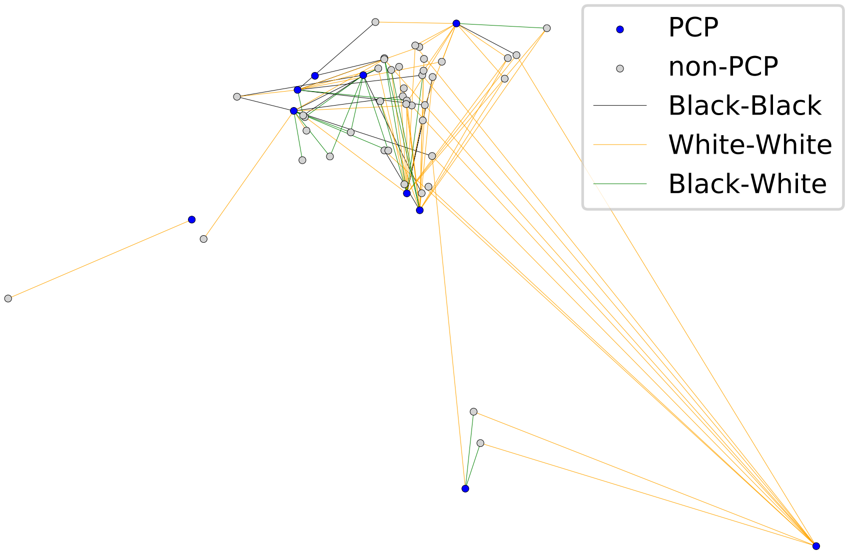

© 2021 Landon BE et al. JAMA Network Open.

HRR: 10; Specialty: 5; Sample: 1

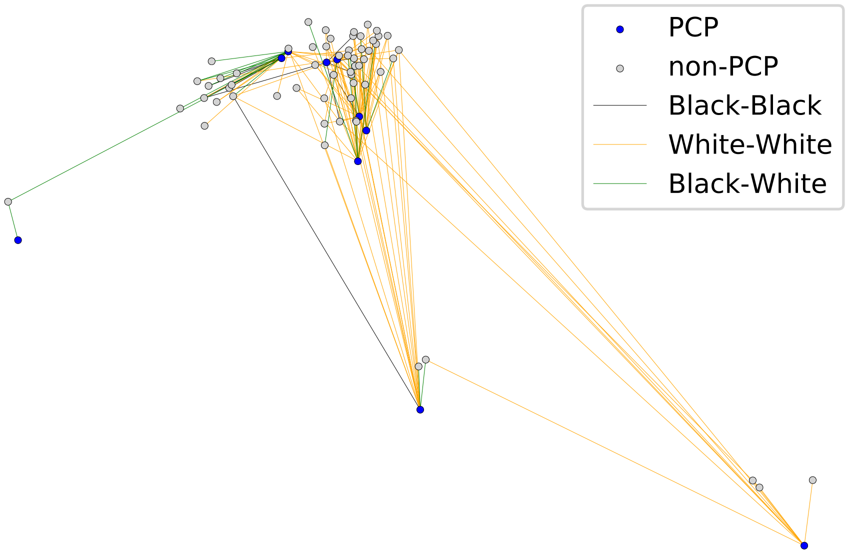

© 2021 Landon BE et al. JAMA Network Open.

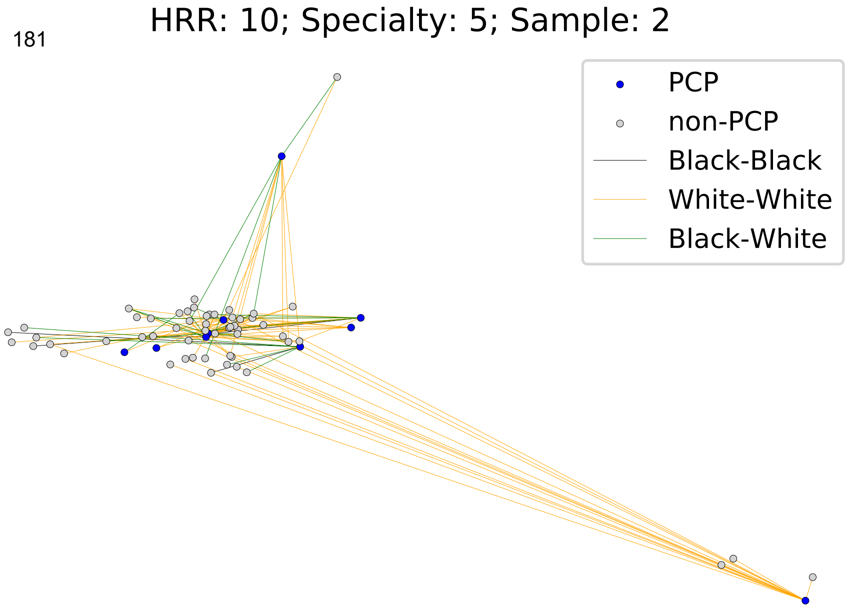

© 2021 Landon BE et al. JAMA Network Open.

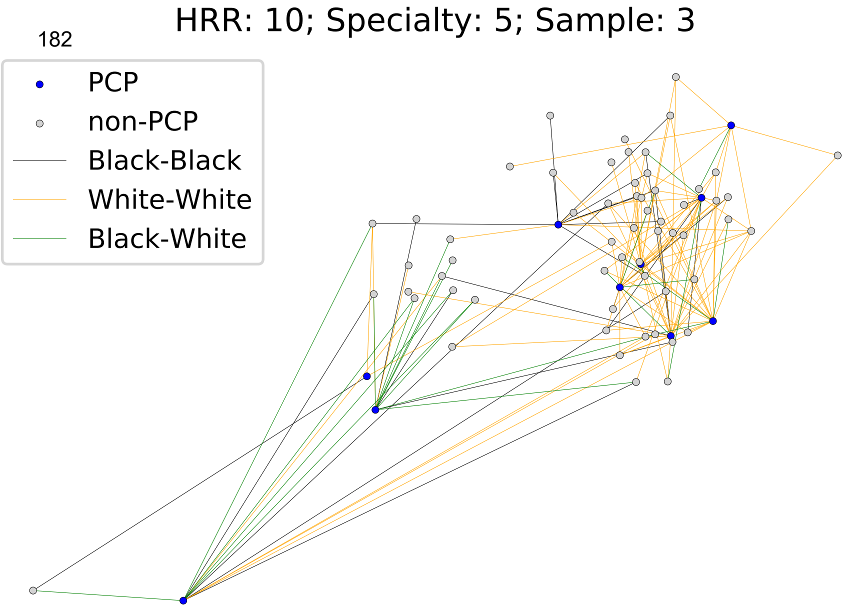

© 2021 Landon BE et al. JAMA Network Open.

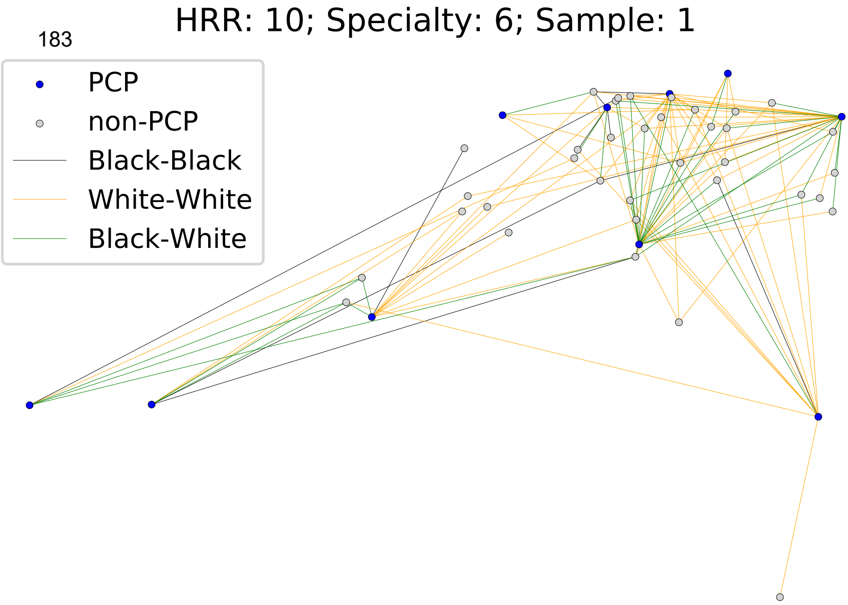

© 2021 Landon BE et al. JAMA Network Open.

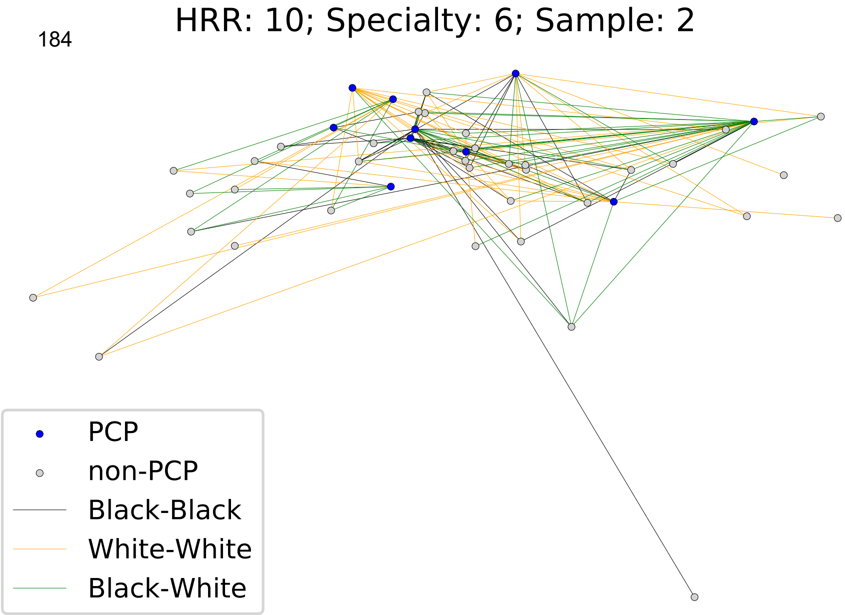

© 2021 Landon BE et al. JAMA Network Open.

HRR: 10; Specialty: 6; Sample: 3

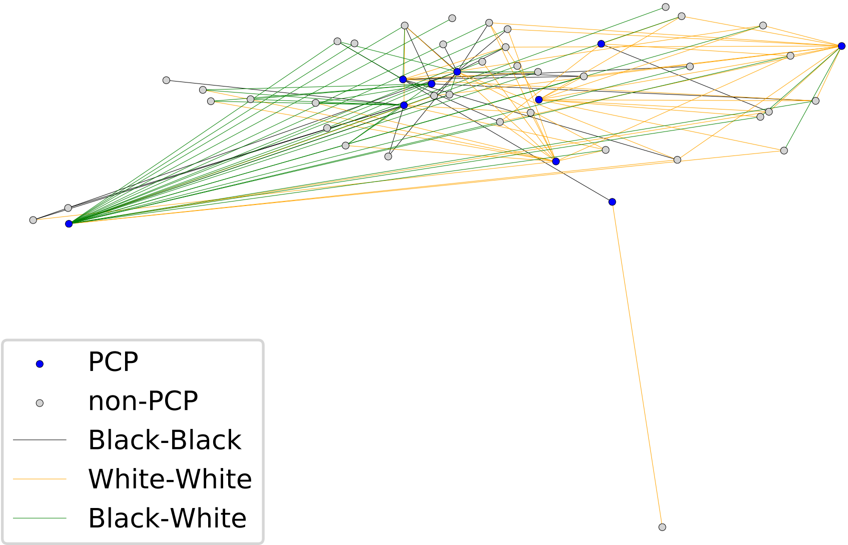

© 2021 Landon BE et al. JAMA Network Open.

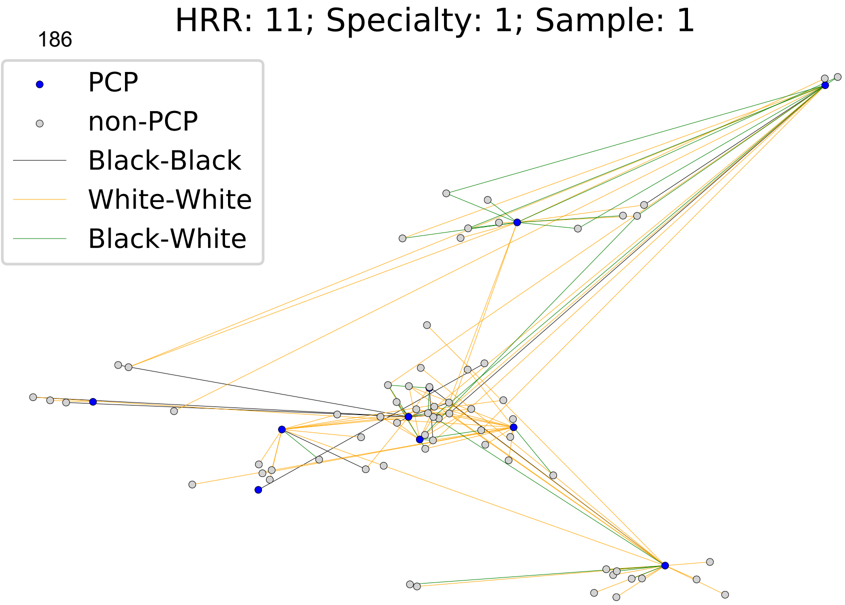

© 2021 Landon BE et al. JAMA Network Open.

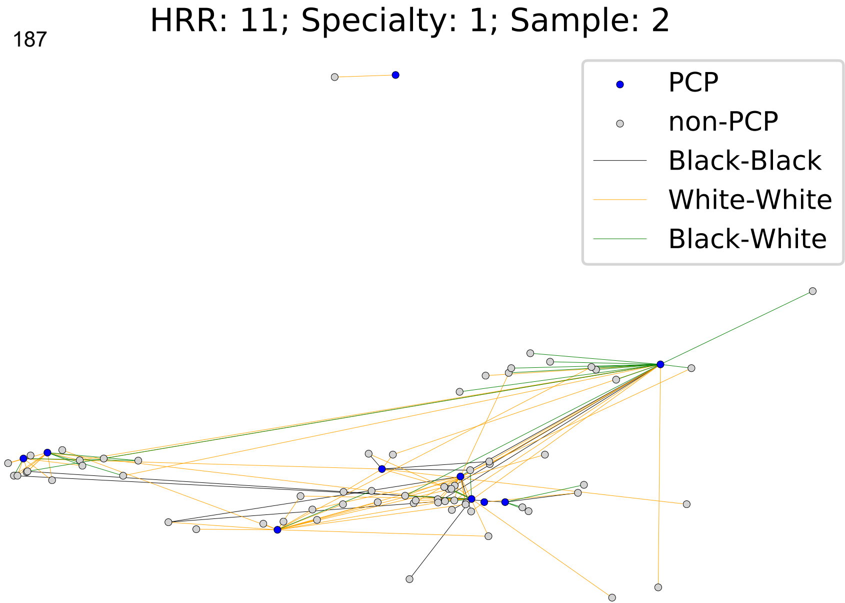

© 2021 Landon BE et al. JAMA Network Open.

HRR: 11; Specialty: 1; Sample: 3

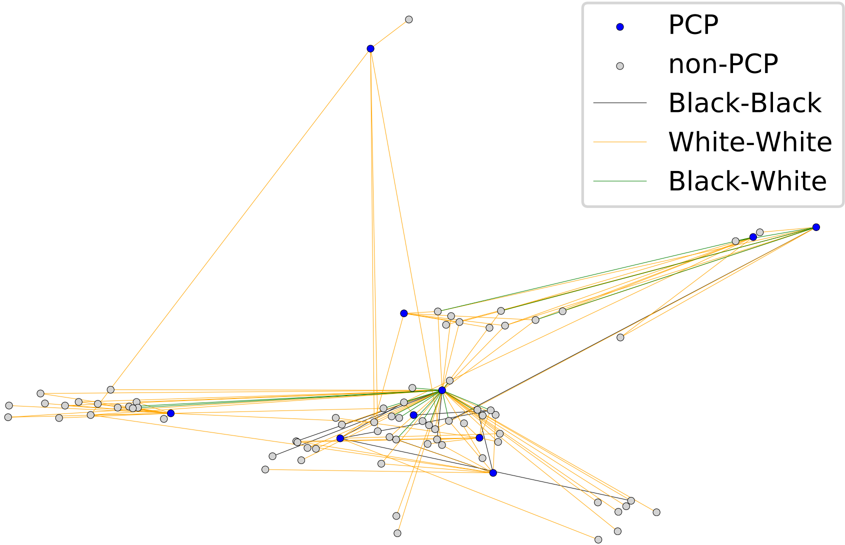

© 2021 Landon BE et al. JAMA Network Open.

HRR: 11; Specialty: 2; Sample: 1

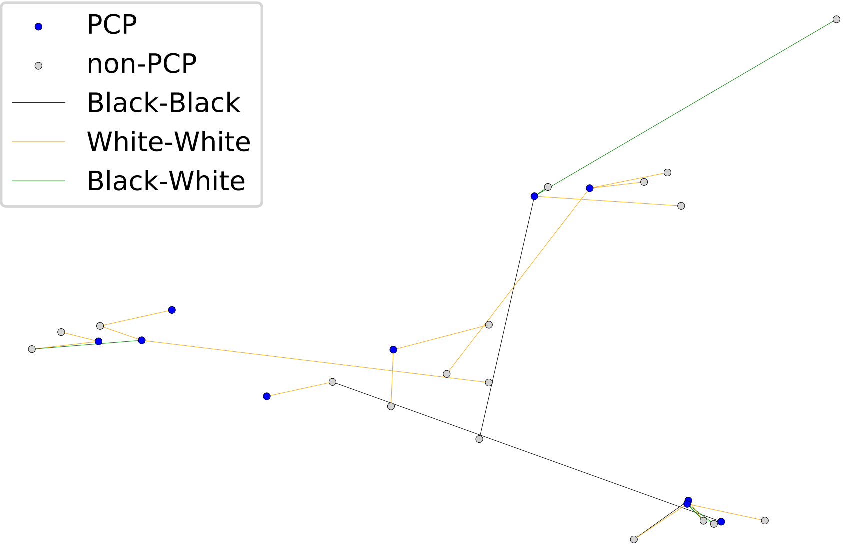

© 2021 Landon BE et al. JAMA Network Open.

HRR: 11; Specialty: 2; Sample: 2

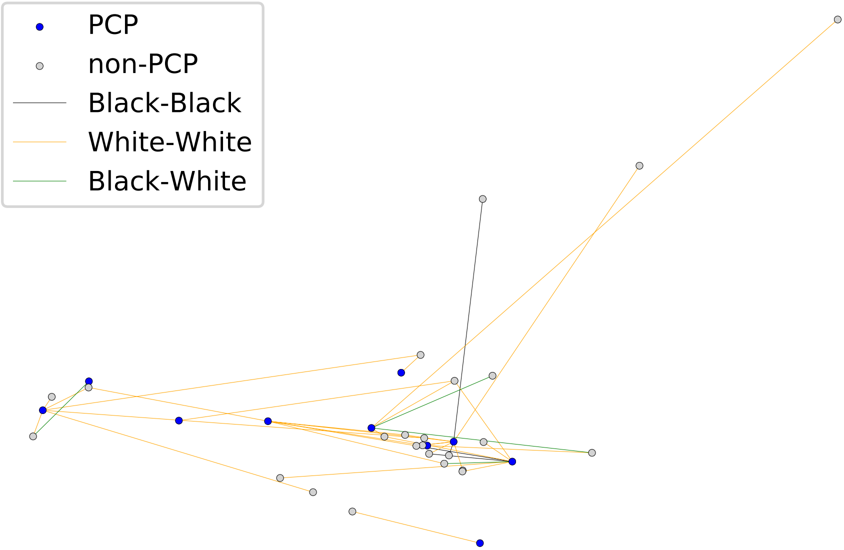

© 2021 Landon BE et al. JAMA Network Open.

HRR: 11; Specialty: 2; Sample: 3

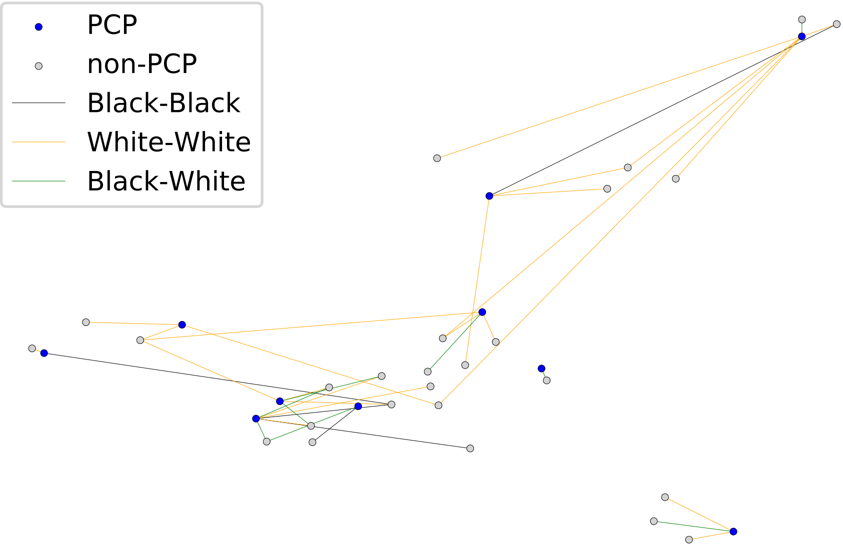

© 2021 Landon BE et al. JAMA Network Open.

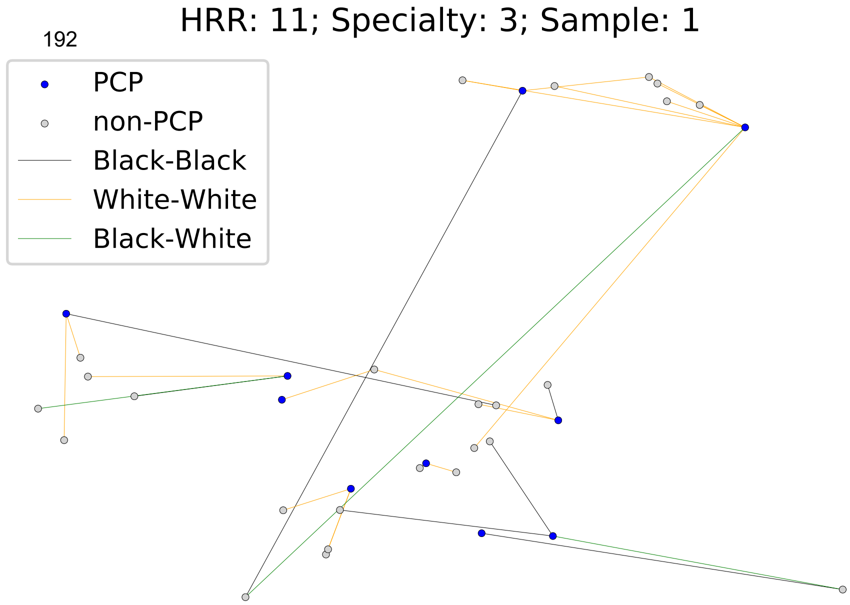

© 2021 Landon BE et al. JAMA Network Open.

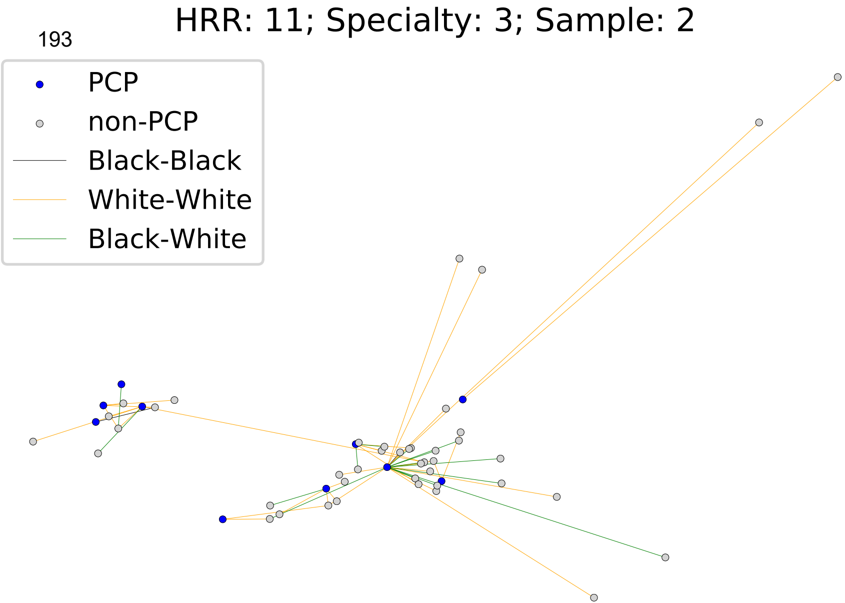

© 2021 Landon BE et al. JAMA Network Open.

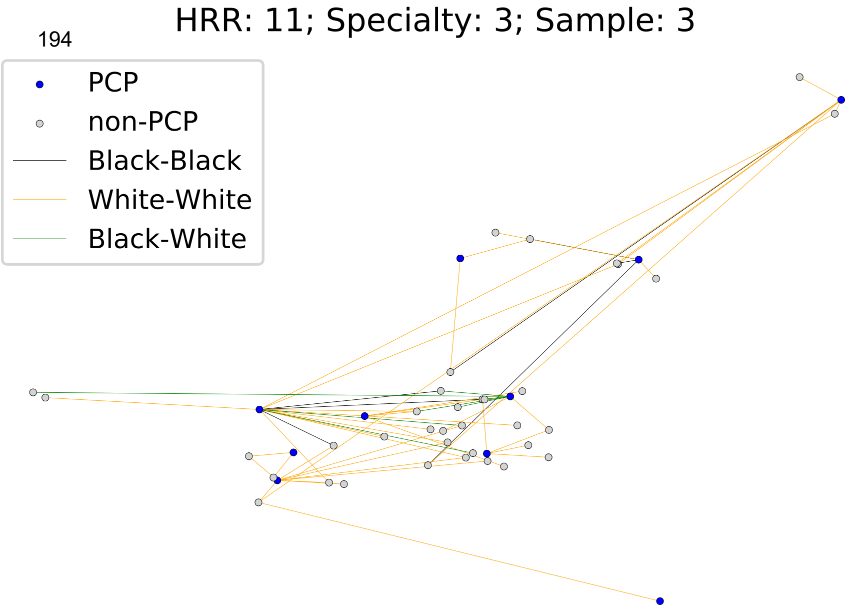

© 2021 Landon BE et al. JAMA Network Open.

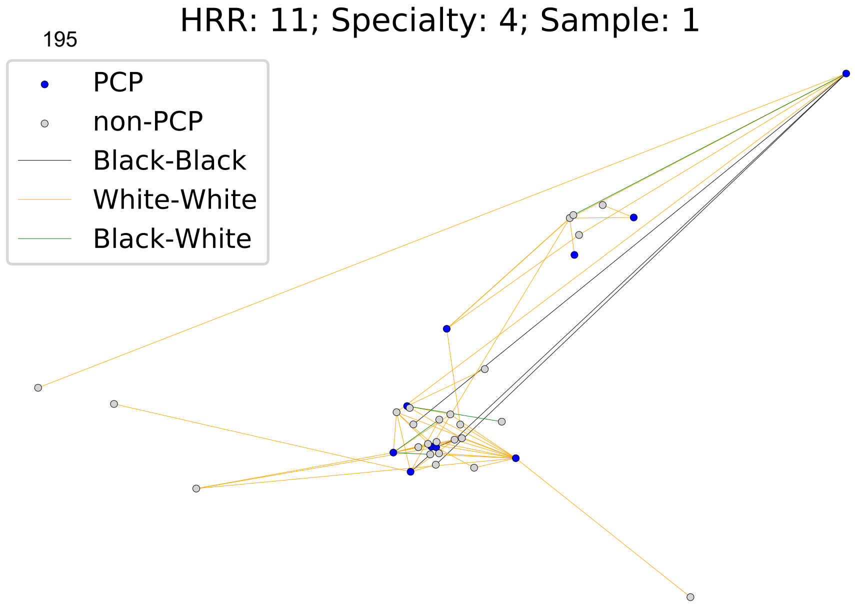

© 2021 Landon BE et al. JAMA Network Open.

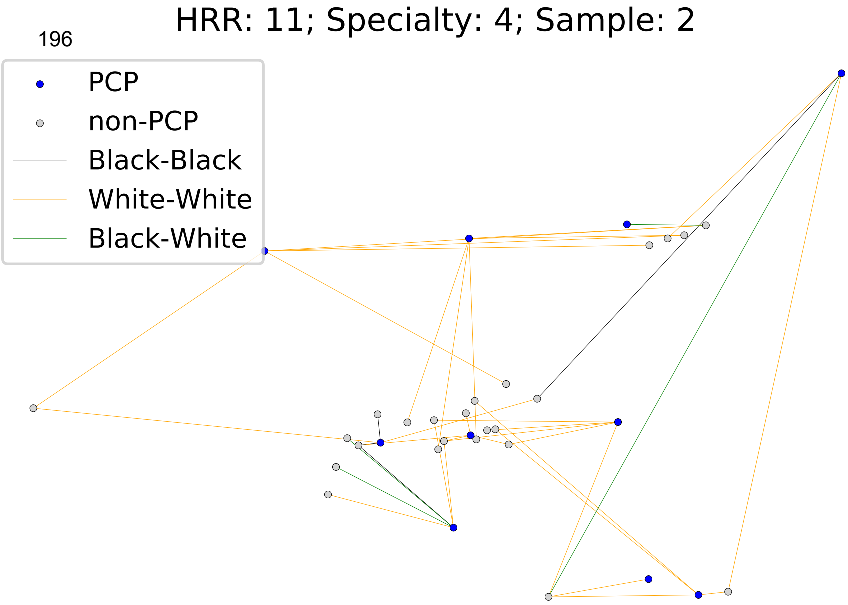

© 2021 Landon BE et al. JAMA Network Open.

HRR: 11; Specialty: 4; Sample: 3

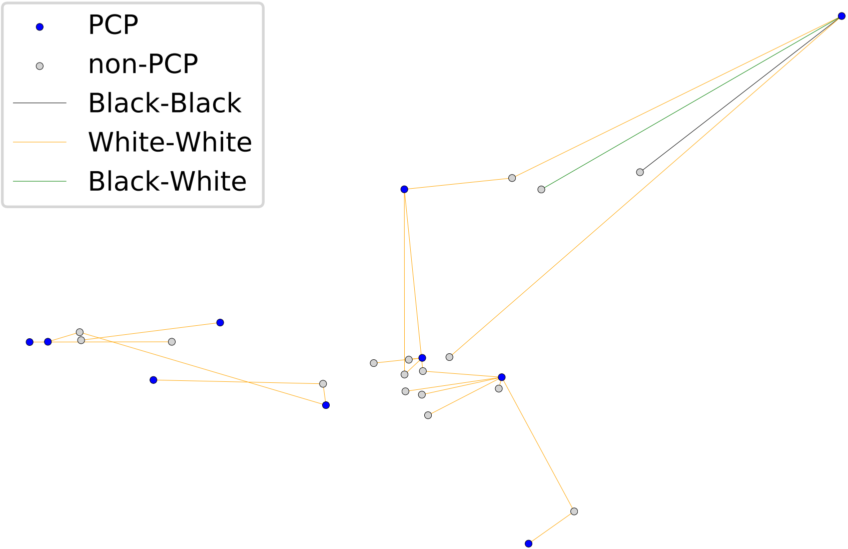

© 2021 Landon BE et al. JAMA Network Open.

HRR: 11; Specialty: 5; Sample: 1

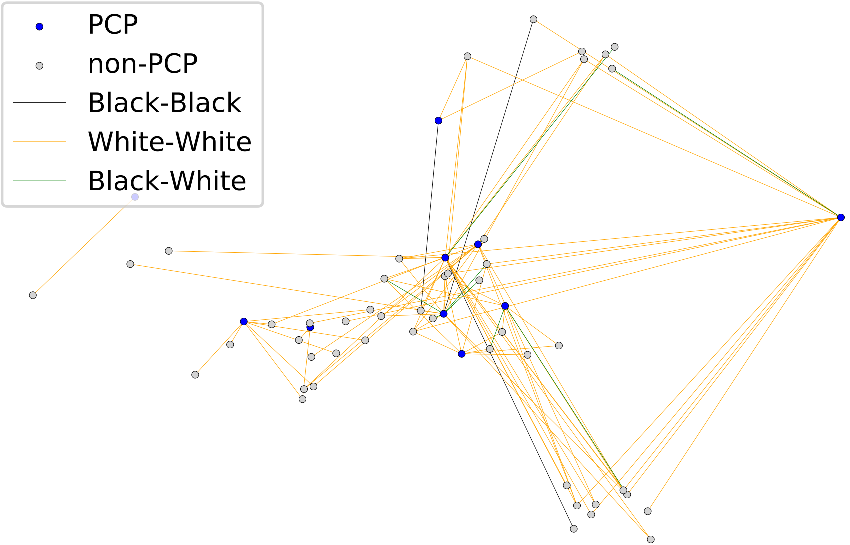

© 2021 Landon BE et al. JAMA Network Open.

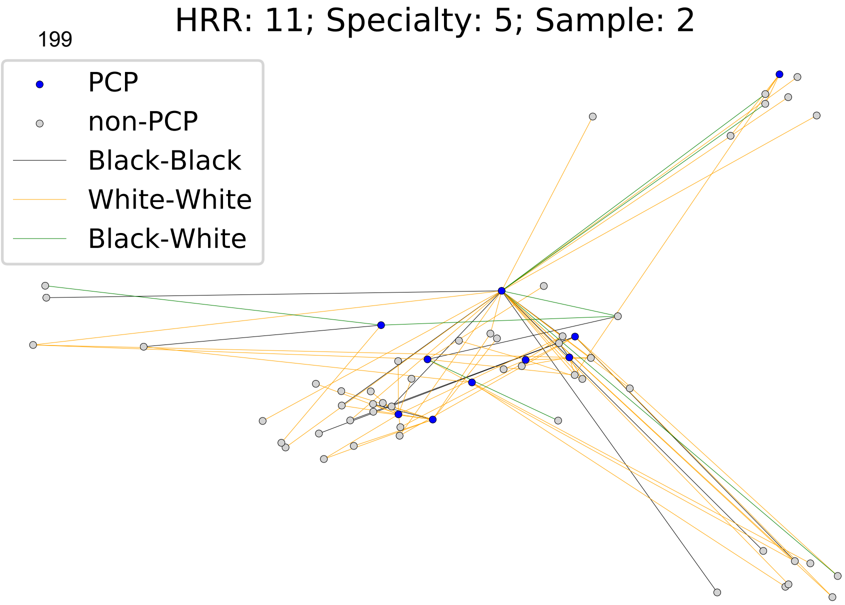

© 2021 Landon BE et al. JAMA Network Open.

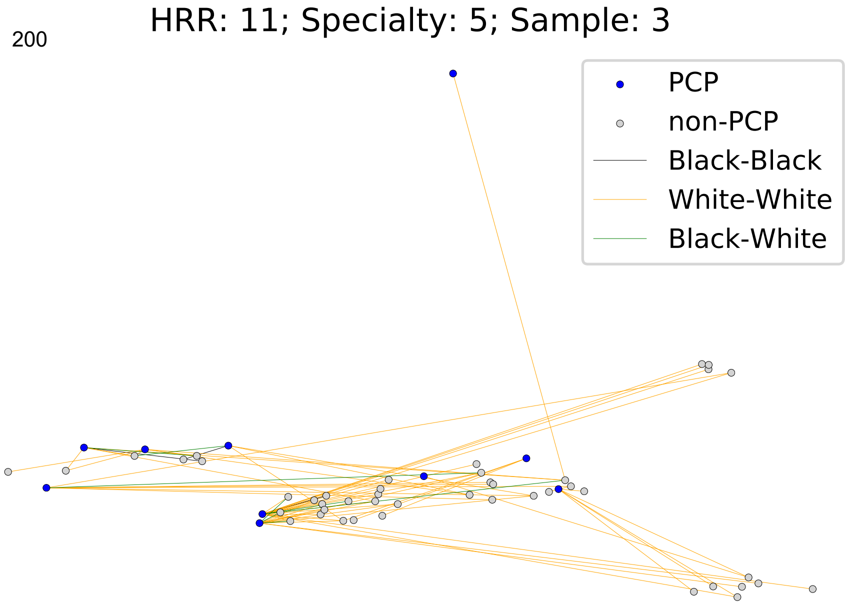

© 2021 Landon BE et al. JAMA Network Open.

HRR: 11; Specialty: 6; Sample: 1

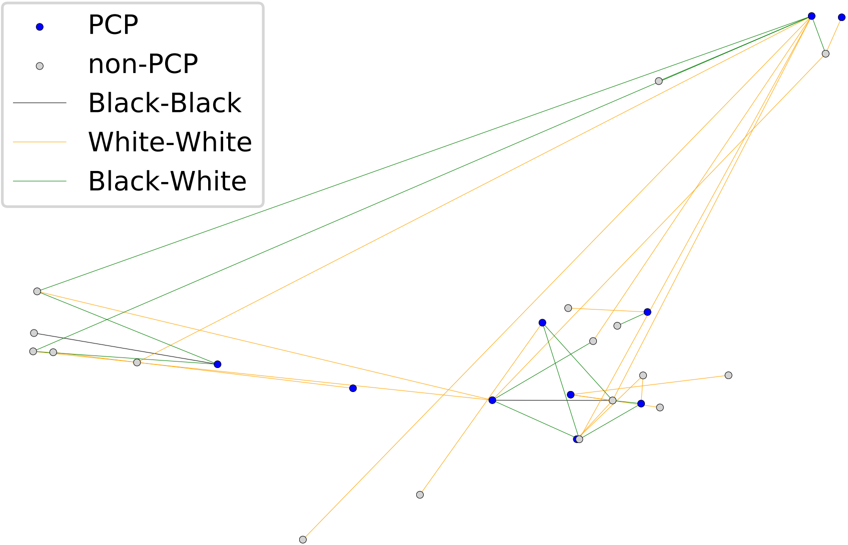

© 2021 Landon BE et al. JAMA Network Open.

HRR: 11; Specialty: 6; Sample: 2

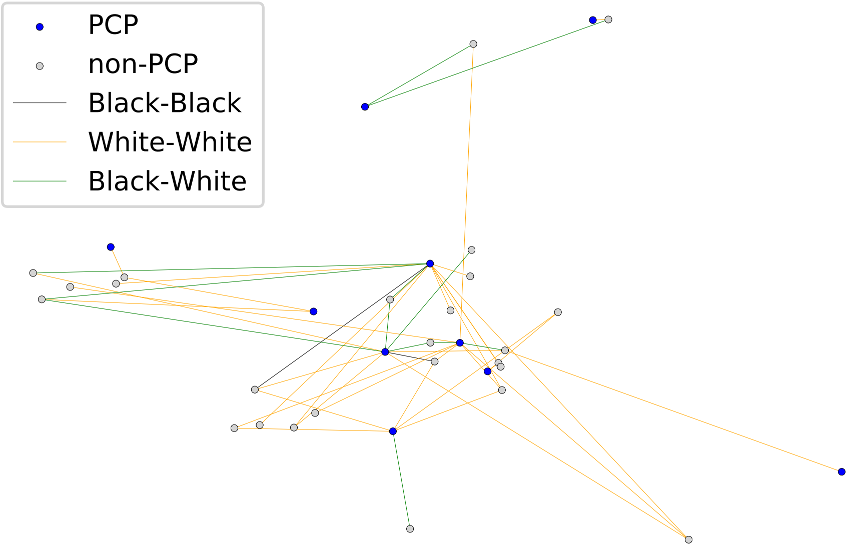

© 2021 Landon BE et al. JAMA Network Open.

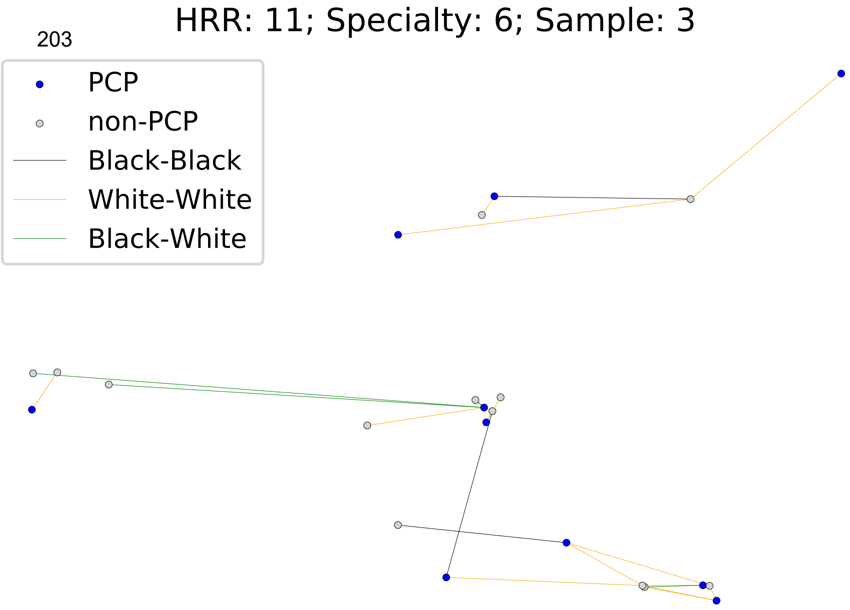

© 2021 Landon BE et al. JAMA Network Open.

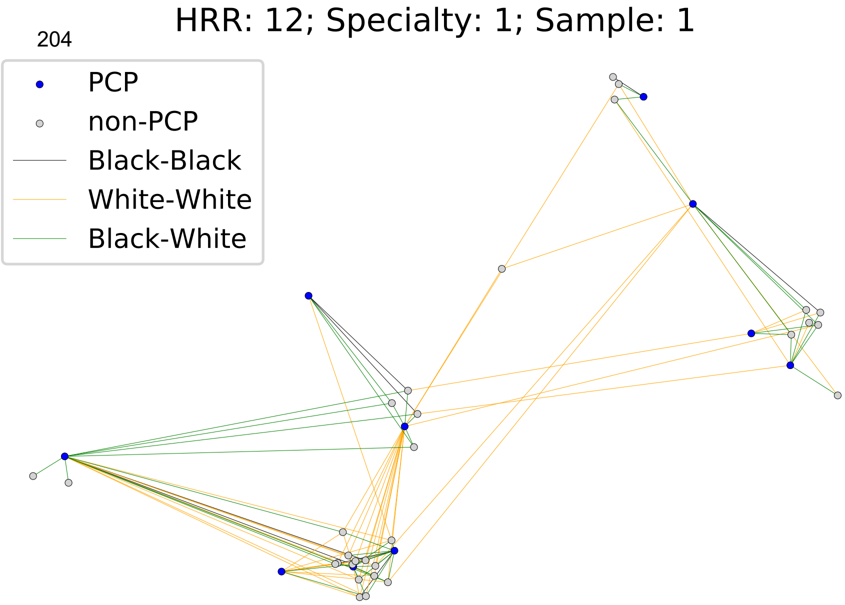

© 2021 Landon BE et al. JAMA Network Open.

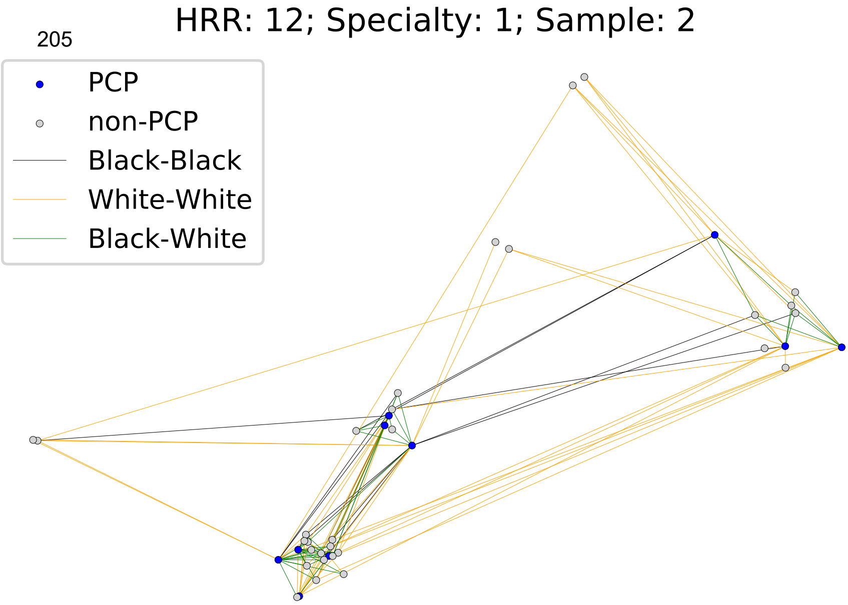

© 2021 Landon BE et al. JAMA Network Open.

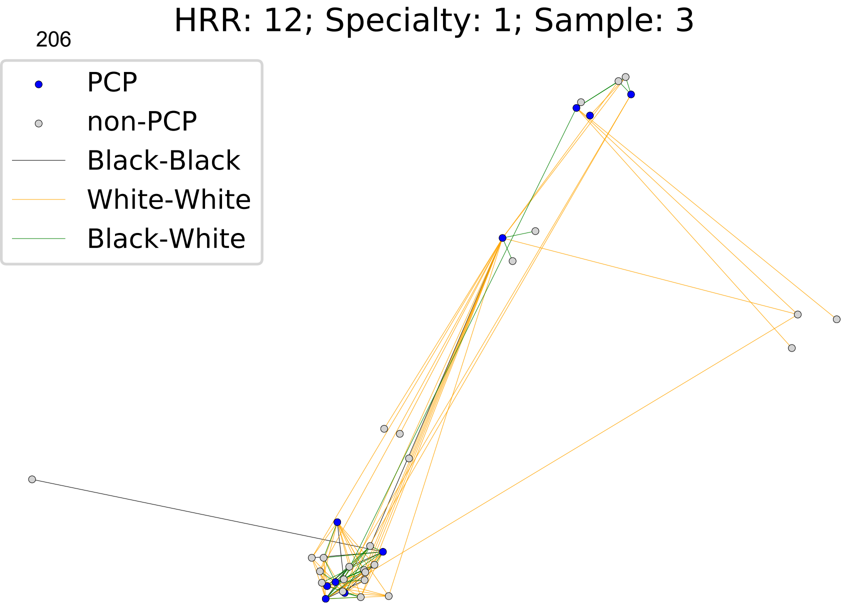

© 2021 Landon BE et al. JAMA Network Open.

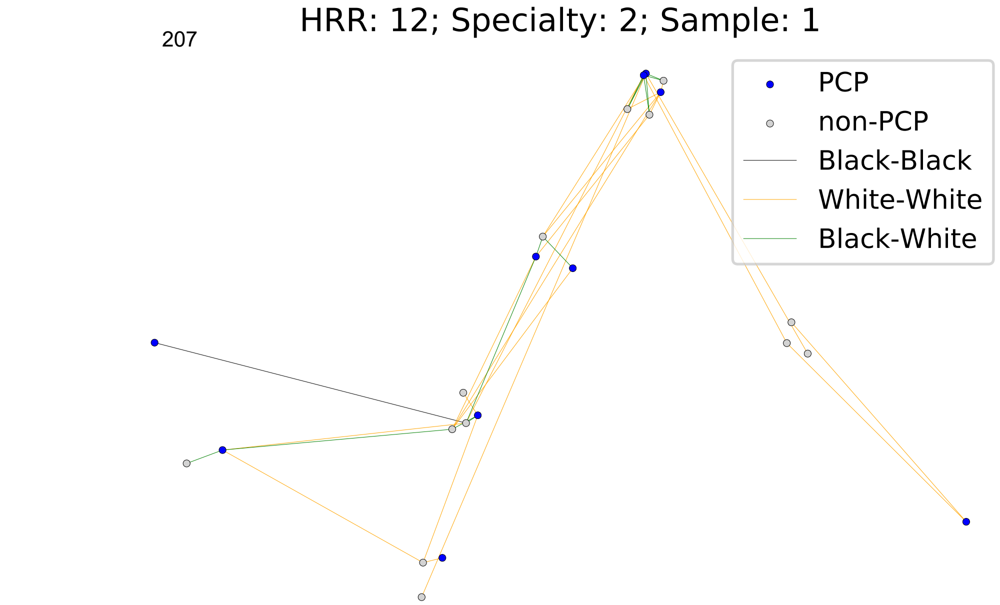

© 2021 Landon BE et al. JAMA Network Open.

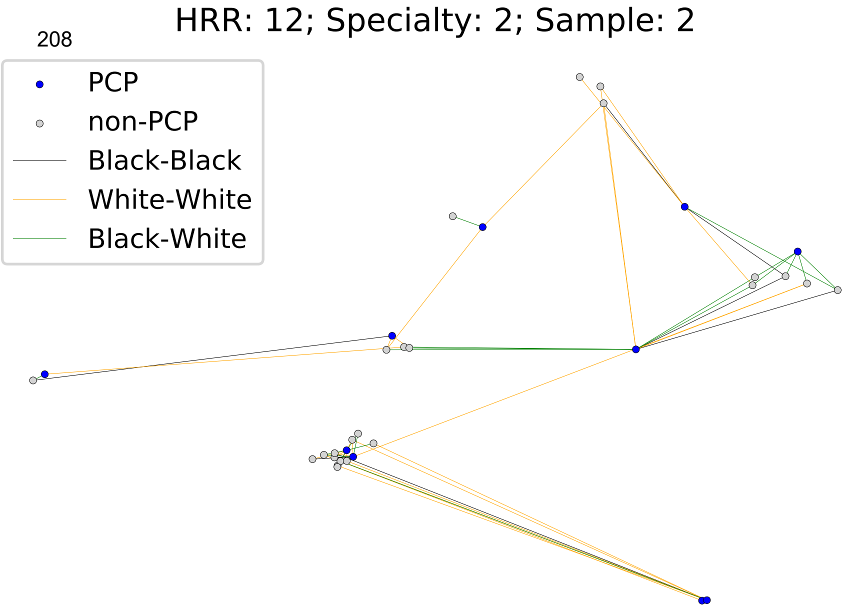

© 2021 Landon BE et al. JAMA Network Open.

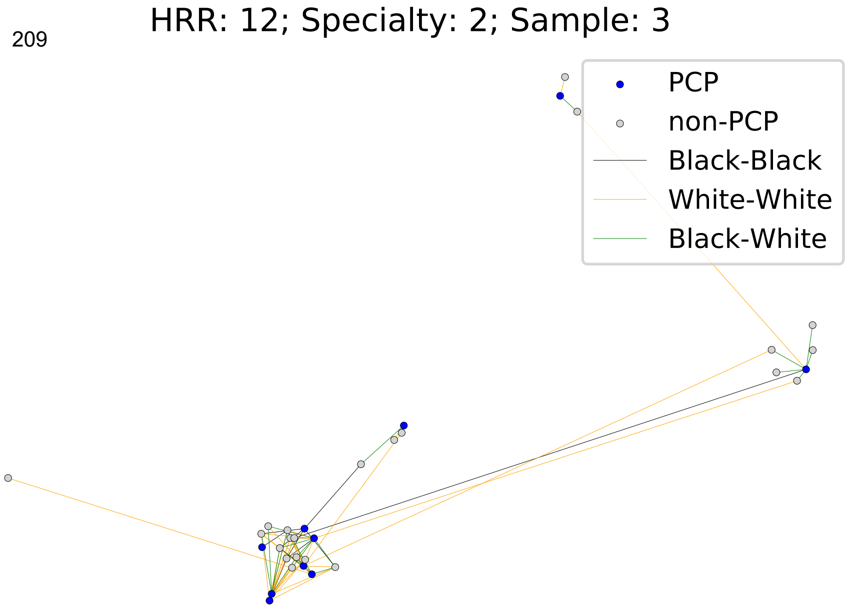

© 2021 Landon BE et al. JAMA Network Open.

HRR: 12; Specialty: 3; Sample: 1

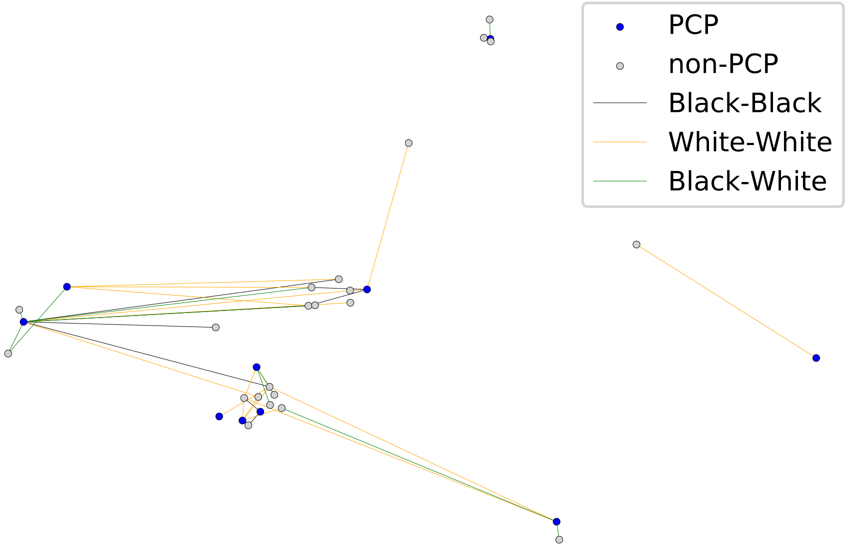

© 2021 Landon BE et al. JAMA Network Open.

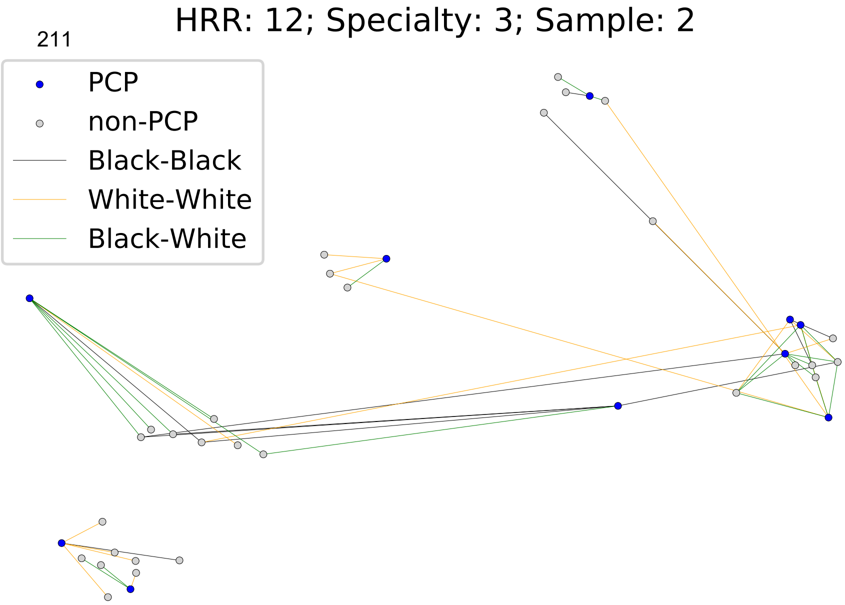

© 2021 Landon BE et al. JAMA Network Open.

HRR: 12; Specialty: 3; Sample: 3

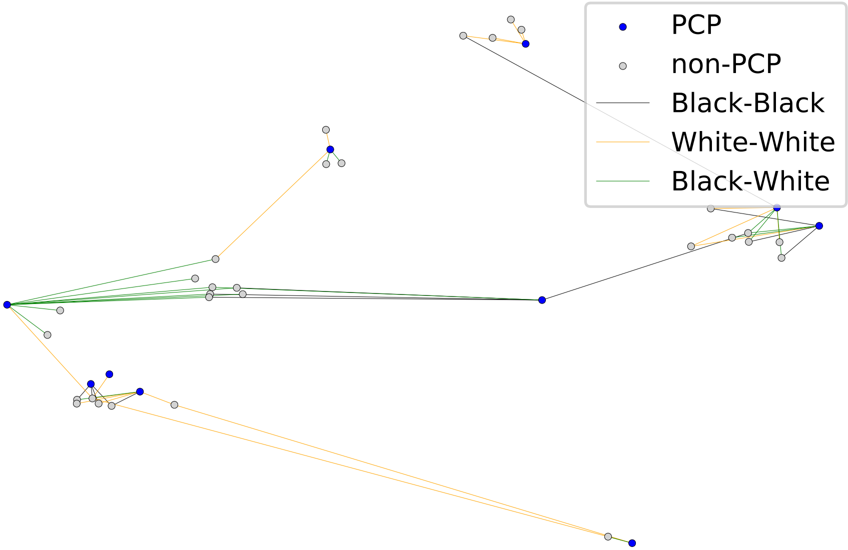

© 2021 Landon BE et al. JAMA Network Open.

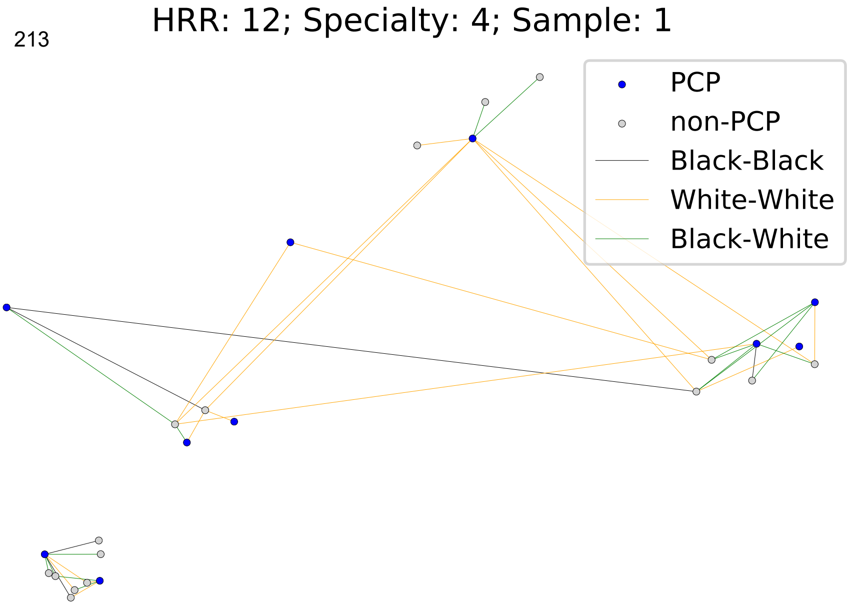

© 2021 Landon BE et al. JAMA Network Open.

HRR: 12; Specialty: 4; Sample: 2

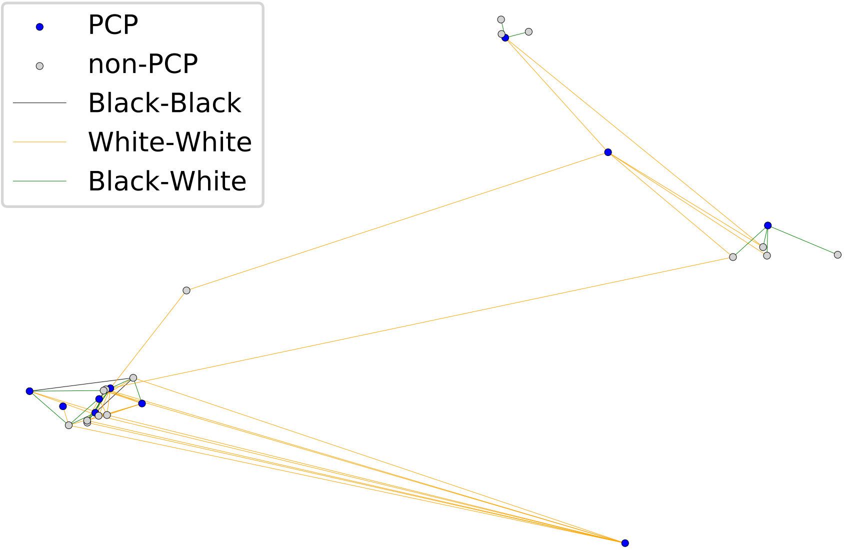

© 2021 Landon BE et al. JAMA Network Open.

HRR: 12; Specialty: 4; Sample: 3

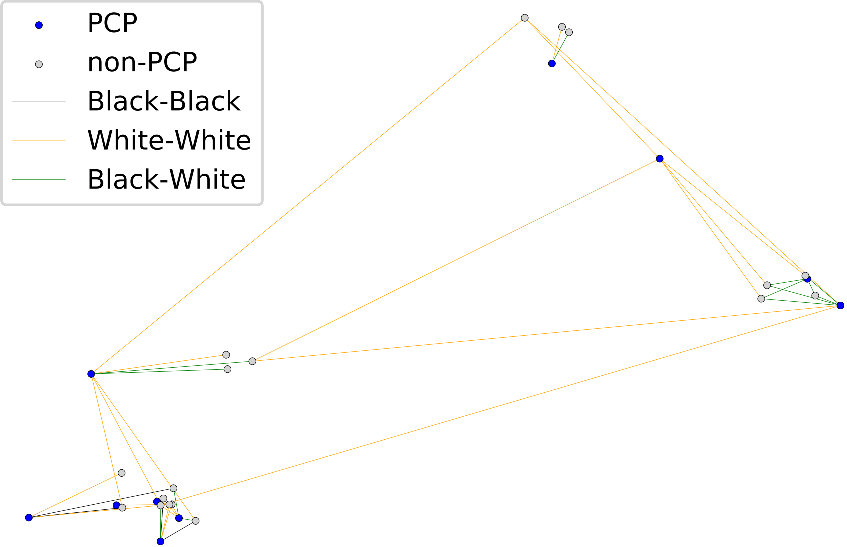

© 2021 Landon BE et al. JAMA Network Open.

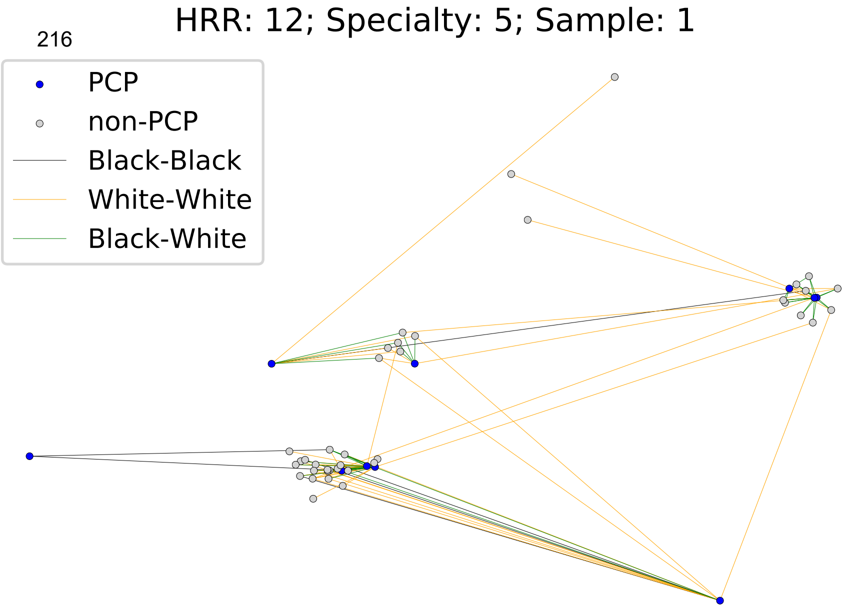

© 2021 Landon BE et al. JAMA Network Open.

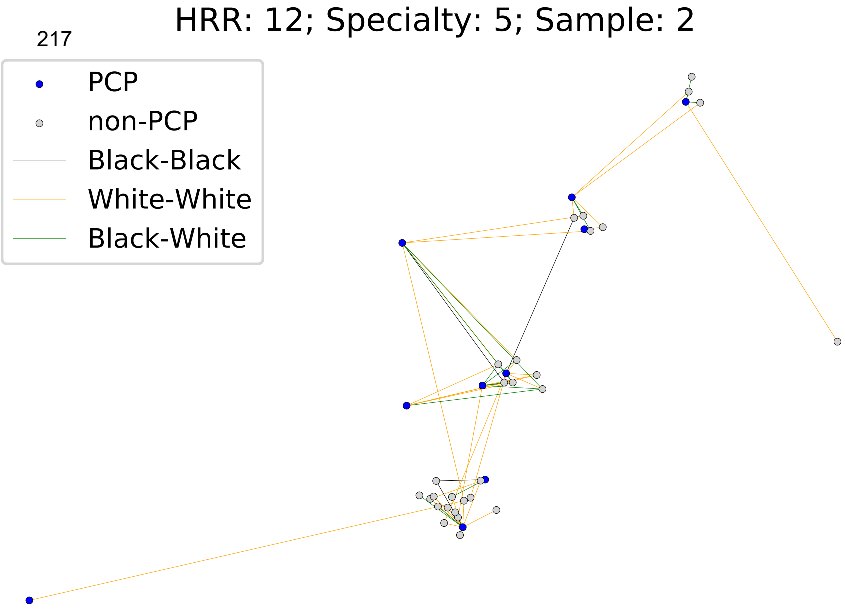

© 2021 Landon BE et al. JAMA Network Open.

HRR: 12; Specialty: 5; Sample: 3

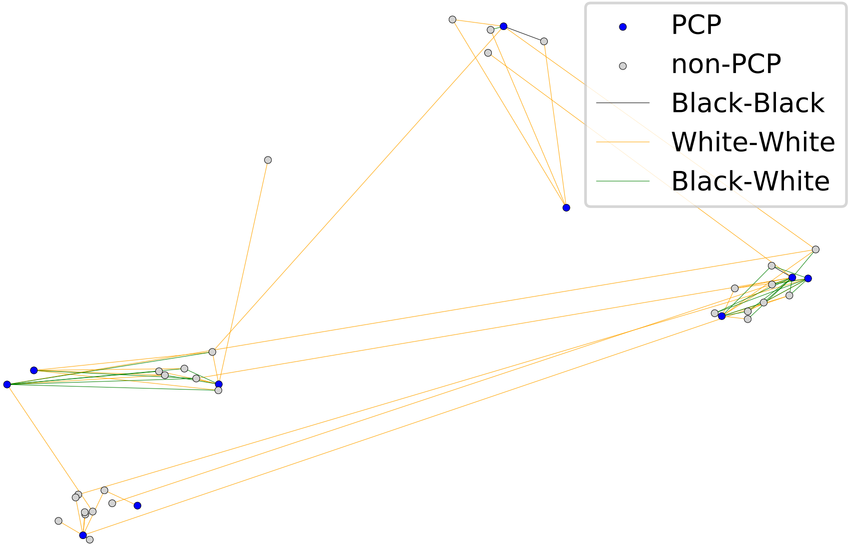

© 2021 Landon BE et al. JAMA Network Open.

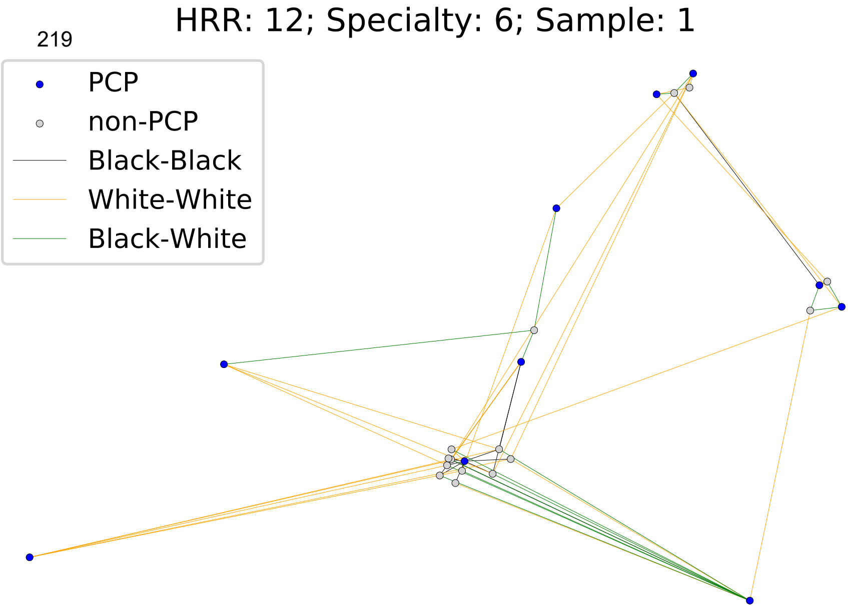

© 2021 Landon BE et al. JAMA Network Open.

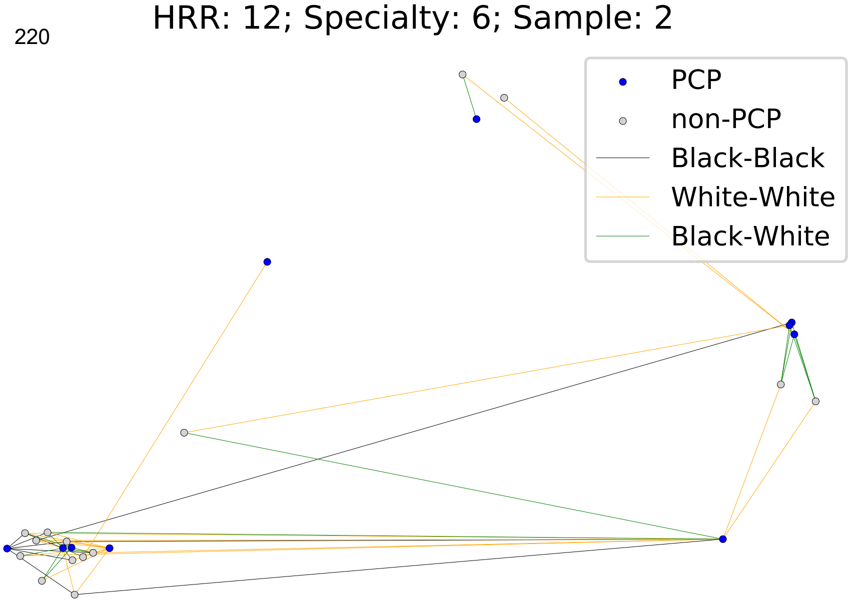

© 2021 Landon BE et al. JAMA Network Open.

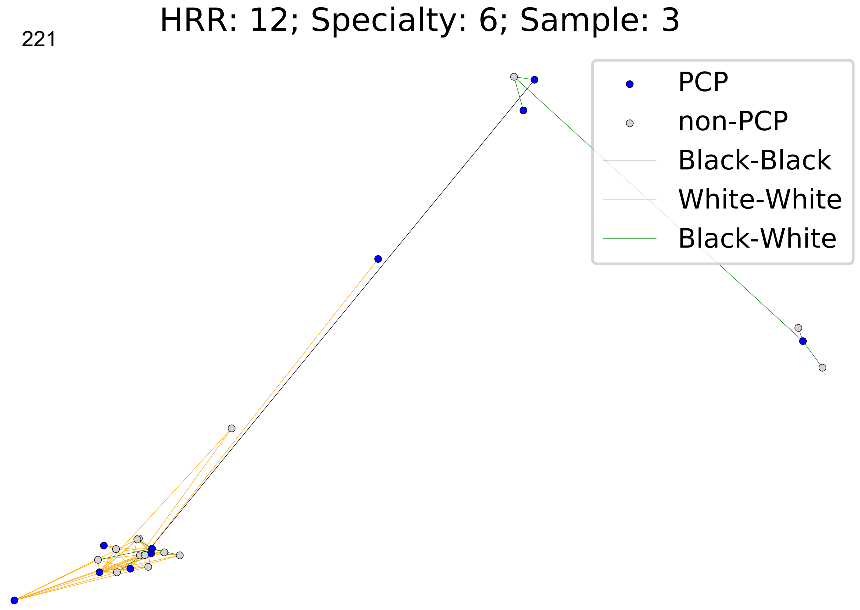

© 2021 Landon BE et al. JAMA Network Open.
